# New insights into tomato microRNAs

**DOI:** 10.1038/s41598-018-34202-3

**Published:** 2018-10-30

**Authors:** Thaís Cunha de Sousa Cardoso, Tamires Caixeta Alves, Carolina Milagres Caneschi, Douglas dos Reis Gomes Santana, Christiane Noronha Fernandes-Brum, Gabriel Lasmar Dos Reis, Matheus Martins Daude, Thales Henrique Cherubino Ribeiro, Miguel Maurício Díaz Gómez, André Almeida Lima, Luiz Antônio Augusto Gomes, Marcos de Souza Gomes, Peterson Elizandro Gandolfi, Laurence Rodrigues do Amaral, Antonio Chalfun-Júnior, Wilson Roberto Maluf, Matheus de Souza Gomes

**Affiliations:** 1 Laboratory of Bioinformatics and Molecular Analysis, Federal University of Uberlandia (UFU), Campus Patos de Minas, 38700-128, Patos de Minas, Brazil; 2 Laboratory of Plant Molecular Physiology, Federal University of Lavras (UFLA), Lavras, 3037 - 37200-000, Brazil; 3 Department of Agriculture, Federal University of Lavras (UFLA), Lavras, 37 - 37200-000, Brazil; 4 Laboratory of Molecular Analysis, Federal University of Tocantins (UFT), Gurupi, 77402-970, Brazil

## Abstract

Cultivated tomato, *Solanum lycopersicum*, is one of the most common fruits in the global food industry. Together with the wild tomato *Solanum pennellii*, it is widely used for developing better cultivars. MicroRNAs affect mRNA regulation, inhibiting its translation and/or promoting its degradation. Important proteins involved in these processes are ARGONAUTE and DICER. This study aimed to identify and characterize the genes involved in the miRNA processing pathway, miRNA molecules and target genes in both species. We validated the presence of pathway genes and miRNA in different NGS libraries and 6 miRNA families using quantitative RT-PCR. We identified 71 putative proteins *in S*. *lycopersicum* and 108 in *S*. *pennellii* likely involved in small RNAs processing. Of these, 29 and 32 participate in miRNA processing pathways, respectively. We identified 343 mature miRNAs, 226 pre-miRNAs in 87 families, including 192 miRNAs, which were not previously identified, belonging to 38 new families in *S*. *lycopersicum*. In *S*. *pennellii*, we found 388 mature miRNAs and 234 pre-miRNAs contained in 85 families. All miRNAs found in *S*. *pennellii* were unpublished, being identified for the first time in our study. Furthermore, we identified 2471 and 3462 different miRNA target in *S. lycopersicum* and *S*. *pennellii*, respectively.

## Introduction

The *Solanaceae* family is one of the largest families in the plant kingdom, including several plants of agronomic and medical importance. It is composed of approximately 100 genera and more than 3000 species that grow in all habitats, ranging from rainforests to deserts. Tomato is one of the most important fruit crops in the global food industry^1–5^. The genus *Solanum* is one of largest genera among the *Angiosperms*, containing approximately 1500 species^6^. *Solanum lycopersicum*, the cultivated tomato is one of the most consumed fruits worldwide^7,8^. In addition to its importance as food, the tomato has important agronomic characteristics, including fleshy fruits and multicellular and glandular trichomes, which model plants, like *Arabidopsis thaliana*, do not have. Thus, the common tomato has been used as a model for species that also have these characteristics^9–13^.

Due to the specific characteristics and growing conditions, tomato varieties are constantly improved^14^. However, this crop is susceptible to insect attacks and more than 200 diseases caused by various types of pathogens, including viruses, bacteria, fungi and nematodes^15^. To aid in the control of these diseases, most producers opt for the use of chemical agents, which are often costly, at times ineffective, and have deleterious effects on the environment^16^. An alternative to these problems is to cross the cultivated tomato with wild species^12,17–20^. There are several known species of wild tomato, consisting of underexploited genetic resources, and of great importance for the improvement, research and development of culture^12,18,20–26^. *Solanum pennellii* is a wild tomato species that has been widely used for the construction and mapping of populations due to its tolerance to environmental stress^18,20,27,28^. In this sense, a better understanding of the tomato molecular basis is required to achieve the efficiency and success in the selection of markers associated with characteristics of interest^4^.

Current progress in sequencing the tomato genome has generated useful information to assist in the study of their genetic diversity^12,23^. The sequencing of the *S*. *lycopersicum* genome was completed in 2012^29^. Furthermore, the availability of a reference genome provided a framework for the genomic analysis of *Solanaceae* family, generating a source of important information for their molecular breeding^5,13,30^. In addition to *S*. *lycopersicum*, the *S*. *pennellii* genome was also sequenced, which provided a valuable resource for the understanding of several prominent features, such as changes in response to water deficit, biotic and abiotic resistance, and metabolism^20,28^. However, various parts of these genomes need to be thoroughly studied and annotated, since the sequencing of the genome and the deposit of the sequences in public databases are only the initial steps for a thorough understanding.

Although much is known about tomato biology, relatively little is known about the regulation of gene expression involved in plant development. Recent studies have shown the importance of gene regulation involving several classes of small RNAs, their processing system and cellular performance in different organisms^31–36^. The main representatives of this class of small RNAs are the microRNAs (miRNAs), whose ways of regulation may involve inhibition of the translation process, degradation of mRNA or gene silencing, either by target complementarity or by signalling DNA modifications in specific regions of genome^37–42^. miRNAs are endogenous small RNAs with approximately 21 nucleotides in length in mature form^37,43,44^.

Different proteins act to generate specific mature miRNAs for post-transcriptional regulation^32,45,46^. In plants, miRNA processing first occurs in the nucleus from the transcription resulting in a long primary miRNA (pri-miRNA) which after cleavage generates the miRNA precursor (pre-miRNA)^43,47–54^. The pre-miRNA has a characteristic secondary structure, forming a hairpin via an imperfect complementarity, containing 200–300 nucleotides in length^55,56^. The pre-miRNA is also cleaved at the end of the hairpin, forming a duplex of mature miRNAs (miRNA/miRNA* double strand). The mature miRNA is exported to the cytoplasm and then loaded in a RISC complex, for specific recognition of the target mRNA to regulate gene expression either by translation inhibition or mRNA degradation^32,48,55,57–59^.

Important biological functions are mediated by miRNAs, which include the adaptation of plants to stress, regulation of leaf and floral development, cell defence, cell proliferation, apoptosis and signal transduction^32,43,47,60–62^. Due to important and complex regulation mediated by the miRNAs, the expression detection and quantification of these molecules in specific tissues is essential for a better understanding of their action. Although miRNAs are transcribed at high expression levels, the latter fluctuate widely between cells and among tissues. A sensitive and rapid method for the detection and quantification of miRNA expression is the quantitative real-time PCR (qRT-PCR)^63–65^. In addition, several computational strategies have been used to identify miRNAs and proteins involved in their pathways in various organisms^66–68^. Computational methods are widely used in gene identification, since some transcripts are expressed only under certain conditions or in specific cells. Thus, the computational techniques aid in the discovery process of new miRNAs, using all the information contained in the genome and/or the transcriptome independently of the sampling. Furthermore, such methods are useful for the prediction of miRNA precursors and their targets.

Previous studies have identified miRNAs and proteins involved in miRNA biogenesis in *S*. *lycopersicum*^7,69–86^. Many of these studies have shown important roles of tomato miRNAs, such as those seen in fruit ripening^77^, response to long-term RPM-treatment^87^, response to curl *New Delhi virus* (ToLCNDV)^75^, responses associated to *Botrytis cinerea* infection^82^, male-sterility^84^, salt-induced stress response^78^, as well as biotic^79^, drought^83^, and high-temperature stress responses^86^. However, many of these genes, miRNAs and their respective targets have not yet been identified and characterized systematically in tomato species, especially in *S*. *pennellii*. Thus, the study of miRNAs in the plant genome and transcriptome has become a very powerful tool to assist in elucidation of biological processes as well as their performances at the cellular level^67,88–90^.

The aims of this study were to identify and characterize by in silico analysis the genes involved in miRNA processing, the miRNA precursors, the miRNA targets, as well as to validate such miRNAs in different tissues of *S*. *lycopersicum* and *S*. *pennellii*, using genomic and transcriptomic sequences obtained from public databases and RT-PCR. This study allows a better understanding of miRNAs, their processing pathways and their role in regulating the gene expression of these important species, as well as providing targets for future investigations.

## Results

### The small RNAs processing pathway in *S*. *lycopersicum* and *S*. *pennellii*: analysis of conserved protein domains, active sites and phylogenetic trees

To understand potential roles of small RNAs in the regulation of gene expression in tomato, we sought to initially identify all possible components of small RNA processing machinery in *S*. *lycopersicum* and *S*. *pennellii* genomes. Using a local alignment, as well as the presence and arrangement of conserved domains, 71 putative proteins were identified in *S*. *lycopersicum* and 108 in *S*. *pennellii* likely involved in the biogenesis of small RNAs (siRNAs, tasiRNAs and miRNAs) (data not shown). Among these proteins, the AGO and DCL families are considered the key proteins in the processing machinery of small RNAs in plants^47,91^. We identified 15 and 16 proteins, which belong to the ARGONAUTE family in *S*. *lycopersicum* and *S*. *pennellii*, *respectively* (SlyAGO1.1, SlyAGO1.2, SlyAGO2.1, SlyAGO2.2, SlyAGO2.3, SlyAGO4.1, SlyAGO4.2, SlyAGO4.3, SlyAGO4.4, SlyAGO4.5, SlyAGO5, SlyAGO6, SlyAGO7, SlyAGO10.1, SlyAGO10.2, SpeAGO1.1, SpeAGO1.2, SpeAGO2.1, SpeAGO2.2, SpeAGO2.3, SpeAGO4.1, SpeAGO4.2, SpeAGO4.3, SpeAGO4.4, SpeAGO4.5, SpeAGO4.6, SpeAGO4.7, SpeAGO5, SpeAGO6, SpeAGO7, SpeAGO10). Eight and 11 DICER family proteins were found in *S*. *lycopersicum* and *S*. *pennellii*, *respectively* (SlyDCL1, SlyDCL2.1, SlyDCL2.2, SlyDCL2.3, SlyDCL2.4, SlyDCL3.1, SlyDCL3.2, SlyDCL4, SpeDCL1, SpeDCL2.1, SpeDCL2.2, SpeDCL2.3, SpeDCL2.4, SpeDCL2.5, SpeDCL2.6, SpeDCL3, SpeDCL4.1, SpeDCL4.2, SpeDCL4.3).

Among the proteins involved in small RNA processing, 29 putative proteins for *S*. *lycopersicum* and 32 for *S*. *pennellii* were identified that participate in the miRNA pathways (Supplementary Tables S1 and S2).

### ARGONAUTE and DICER proteins in *S*. *lycopersicum* and *S*. *pennellii*

#### Analysis of conserved domains, active sites, phylogeny and pathway proteins and pre-miRNA alignment in *S*. *lycopersicum* and *S*. *pennellii*

We found 15 AGO proteins in the genome of *S*. *lycopersicum* and 16 in *S*. *pennellii*. The SlyAGO1.1/1.2 and SpeAGO1.1/1.2 proteins displayed the Gly-richAgo1, ArgoN, ArgoL1, PAZ, ArgoL2, ArgoMid and Piwi domains. Similar results were observed for SlyAGO10.1/10.2 and SpeAGO10, which displayed the ArgoN, ArgoL1, PAZ, ArgoL2, ArgoMid and Piwi domains (Supplementary Fig. S1 and Table S3).

**Figure 1:**
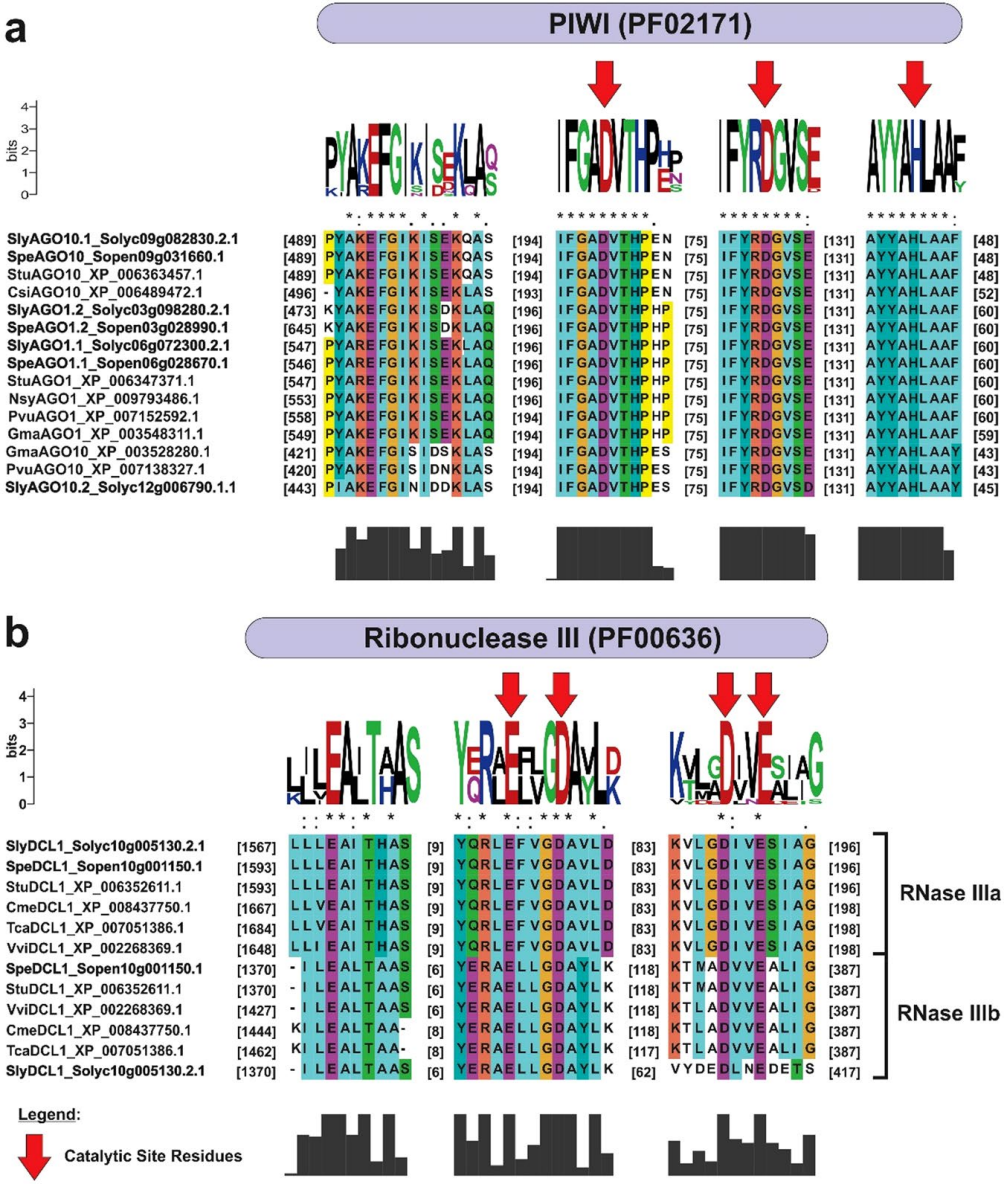
Weblogo analysis and sequence multiple alignment of active site amino acids and their flanking amino acid residues in the conserved domains of *S*. *lycopersicum*, *S*. *pennellii* and their orthologs. (**a**) Piwi domain (PF02171) from AGO proteins and (**b**) Ribonuclease III domain (PF00636) from DCL proteins. The red arrows show the amino acid residues of the active sites (catalytic triad AGO: Asp-Asp-His and DCL: Glu-Asp-Asp-Glu). The height of each amino acid symbol in Weblogo indicates amino acid residue conservation at a given position. The asterisk indicates 100% conserved amino acids at a specific position. Sly - *Solanum lycopersicum*, Spe - *Solanum pennellii*, Csi - *Citrus sinensis*, Gma - *Glycine max*, Pvu - *Phaseolus vulgaris*, Stu - *Solanum tuberosum*, Nsy - *Nicotiana sylvestris*, Cme - *Cucumis melo*, Tca - *Theobroma cacao* and Vvi - *Vitis vinifera*.

The Piwi conserved domain, one of the main domains present in AGO proteins, was identified in SlyAGO1.1, SlyAGO1.2, SpeAGO1.1, SpeAGO1.2, SlyAGO10.1, SlyAGO10.2 and SpeAGO10 proteins. These domains showed active site regions containing an important catalytic triad DDH (aspartate/aspartate/histidine). The DDH catalytic triad of these proteins was found in Asp764-Asp850-His990, Asp690-Asp776-His916, Asp763-Asp849-His989, Asp862-Asp948-His1088, Asp704-Asp790-His930, Asp658-Asp744-His884, Asp704-Asp790-His930 positions, respectively (Fig. 1a).

In *S*. *lycopersicum* and *S*. *pennellii*, we identified 8 and 11 putative proteins, respectively, belonging to the family of DCL proteins involved in the miRNAs processing. We found DCL1 in *S*. *lycopersicum* and *S*. *pennellii*. Both proteins displayed the conserved ResIII (also known as DEAD-like), helicase C, Dicer dimer (also known as DUF283), PAZ, ribonuclease IIIa (RNase IIIa), ribonuclease IIIb (RNase IIIb), dsrm and DND1 DSRM domains (Supplementary Fig. S1 and Table S4).

We found two RNAse III domains in SlyDCL1 and SpeDCL1 proteins. The two domains (RNase IIIa and RNase IIIb) had the same catalytic residues in both tomato species studied: glutamate (E), aspartate (D), aspartate (D), glutamate (E), representing the EDDE active site. These residues were located in RNase IIIa domains at Glu1591-Asp1595-Asp1687-Glu1690 and Glu1617-Asp1621-Asp1713-Glu1716 positions and the RNase IIIb domains at Glu1390-Asp1394-Asp1465-Glu1468 and Glu1390-Asp1394-Asp1521-Glu1524 positions of the SlyDCL1 and SpeDCL1 proteins, respectively (Fig. 1b).

**Figure 2:**
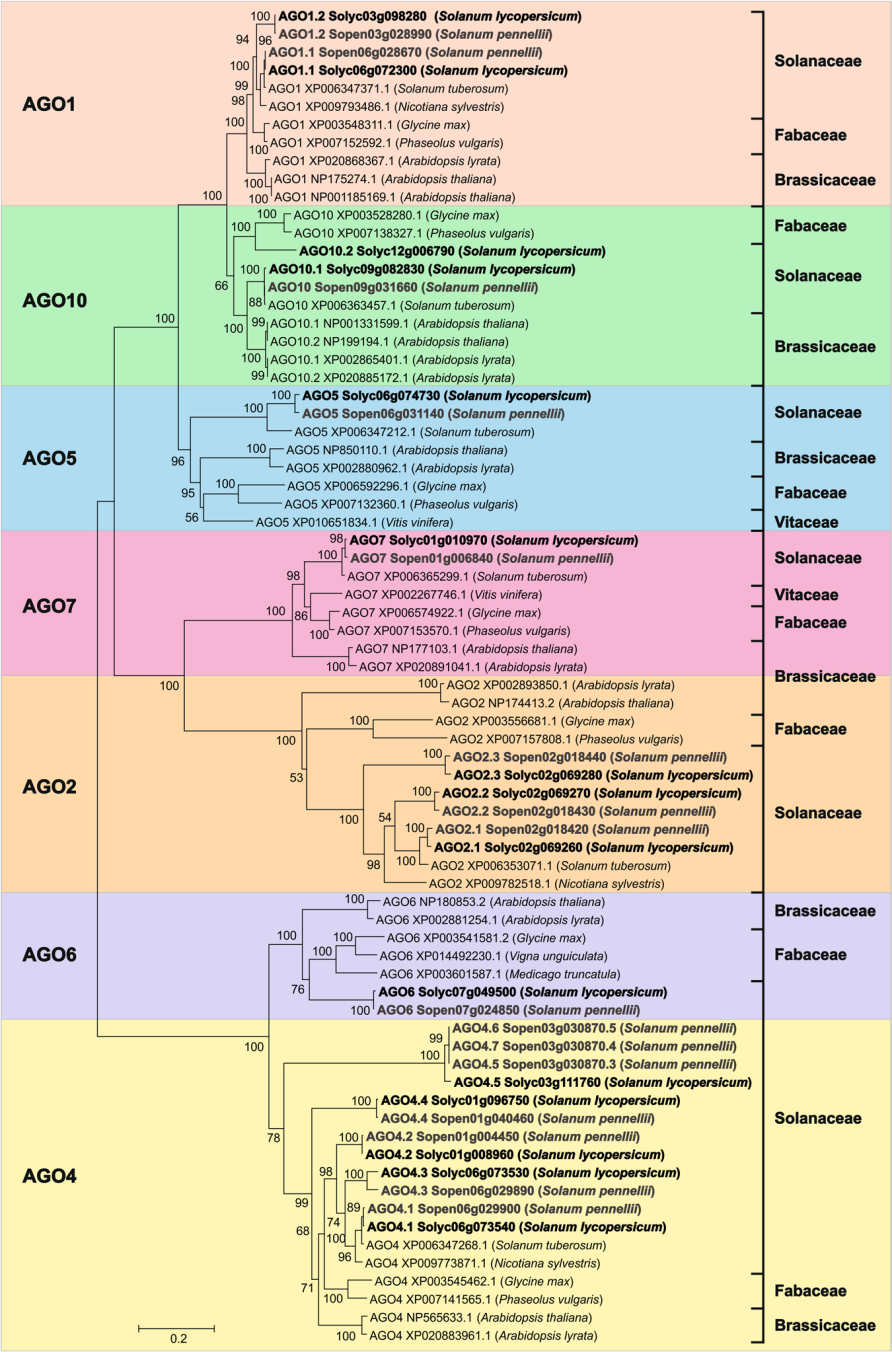
Phylogenetic tree of AGO proteins identified in *S*. *lycopersicum*, *S*. *pennellii* and their orthologs.

**Figure 3:**
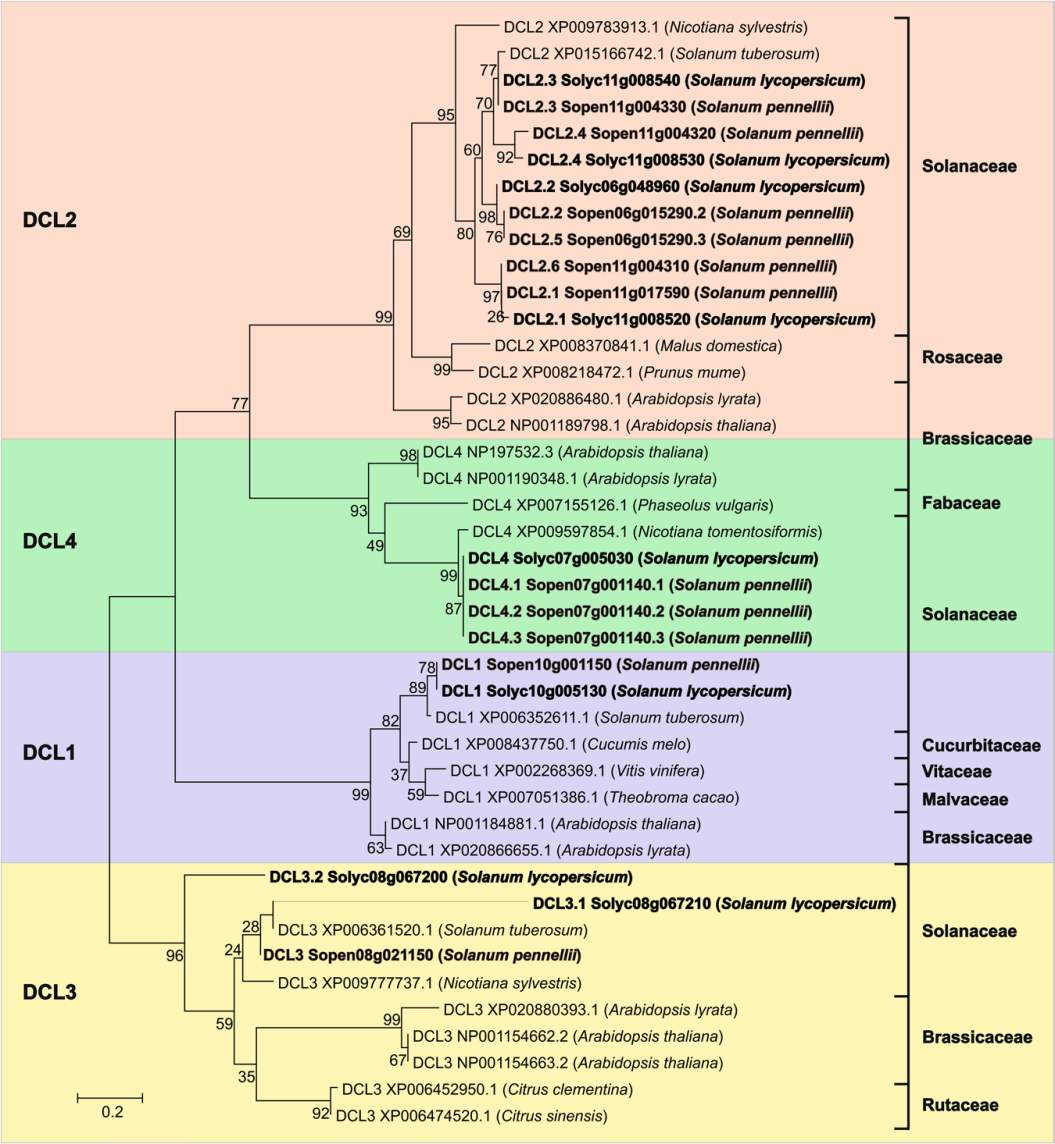
Phylogenetic tree of DCL proteins identified in *S*. *lycopersicum*, *S*. *pennellii* and their orthologs.

To understand the diversification of AGO proteins in *S*. *lycopersicum* and *S*. *pennellii*, a phylogenetic analysis was performed using their amino acid sequences (Fig. 2). The phylogeny of the Sly/SpeAGO1 and Sly/SpeAGO10 proteins showed that they were distributed in the phylogenetic tree closest to their respective orthologous proteins from the plant species *S*. *tuberosum* and *Nicotiniana sylvestris*. The phylogenetic tree was divided into seven distinct clades of paralogous AGOs (AGO1, AGO2, AGO4, AGO5, AGO6, AGO7 and AGO10). In addition, to determine the evolutionary relationship among the putative DCL proteins of *S*. *lycopersicum*, *S*. *pennellii* and their orthologous species, a phylogenetic analysis was also conducted (Fig. 3). The proteins were grouped through global alignment in four distinct clades (DCL1, DCL2, DCL3 and DCL4).

#### Mature miRNAs and their precursors in *S*. *lycopersicum* and *S*. *pennellii*

To show that the miRNA processing machinery is well-conserved in *S*. *lycopersicum* and *S*. *pennellii*, we sought to identify both mature and precursor sequences of miRNAs, in addition to predicting and defining the nature of their putative target genes using available public databases (Phytozome v12.1 and PlaBi). By applying an optimized and specific algorithm (see Materials and Methods), we identified 343 sequences (3p and 5p) of mature miRNAs, out of which 285 were unique miRNA sequences, and 226 precursor miRNAs, distributed in 87 distinct miRNA families in *S*. *lycopersicum* genome (Supplementary Table S5). In *S*. *pennellii*, 388 sequences (3p and 5p) of mature miRNAs were identified, out of which 307 were unique miRNA sequences, and 234 precursor miRNAs were contained in 85 different miRNA families (Supplementary Table S6).

Regarding the miRNA precursor gene localization and distribution in *S*. *lycopersicum* and *S*. *pennellii* genomes, 196 (86.73%) and 195 (81.59%) were found in intergenic regions and 30 (13.27%) and 44 (18.41%) within genes, respectively, whereas in *S*. *pennellii*, 3 pre-miRNA genes are located in more than one position in the genome (Supplementary Table S7). The spe-miR169-1 is located on chromosomes 0 and 7, the spe-miR1919 is in two different positions on chromosome 8 and the spe-miR5368 is on chromosomes 6, 7 and in two different positions on chromosome 9. In addition, it was possible to identify that 72 miRNAs in *S*. *lycopersicum* and 73 in *S*. *pennellii* were organized in clusters (10 kb as the maximum distance between two miRNA genes to consider them clustered, see Materials and Methods). Of these, we identified 50 miRNAs in *S*. *lycopersicum* and 47 miRNAs in *S*. *pennellii* that were organized in clusters, but were located in antiparallel strands (Supplementary Table S7).

All identified miRNAs were analysed for their structural and thermodynamic characteristics. *S*. *lycopersicum* miRNA precursors displayed an MFE with the mean of −56.50 kcal/mol, spanning the values between −98.2 and −21.8 kcal/mol. Such parameters as AMFE and MFEI, had the averages of −39.38 kcal/mol and of 1.03, respectively (Supplementary Table S8). In *S*. *pennellii*, the pre-miRNAs showed an MFE with the mean of −55.60 kcal/mol, with the values between −93.7 and −23.3 kcal/mol, the mean AMFE was −39.13 Kcal/mol and that of MFEI was 1.02 (Supplementary Table S9).

In addition, we performed statistical analyses using thermodynamic and structural characteristics of pre-miRNAs found in both tomato species compared to pre-miRNAs deposited in the miRBase from *Solanaceae*, *Fabaceae* and *Brassicaceae* families. We compared the miRNA precursor characteristics from two tomato species (Supplementary Table S10) and *Solanaceae* family miRNA precursors (Supplementary Table S11), and no differences were observed among the characteristics analysed (*p* < 0.05). In the analysis performed among *S*. *lycopersicum* and *S*. *pennellii* against *Fabaceae* and *Brassicaceae* families, the pre-miRNA characteristics were significantly different (*p* < 0.05).

#### Characterization of tomato miRNAs

Among the 172 miRNA families identified in the two species under study, 71 were found in both tomato species. Of all 101 different miRNA families identified in *S*. *lycopersicum* and *S*. *pennellii*, we highlighted 10 families for more in-depth characterization, since the MIR165/166, MIR167, MIR393, MIR530, MIR827, MIR828 and MIR7983 families are miRNAs conserved in plants, and the MIR7990, MIR8011 and MIR8025 families were identified for the first time in *S*. *lycopersicum* and *S*. *pennellii*. For each of these families, we analysed the conservation of sequences and phylogenetic distributions, as well as their putative target mRNAs.

We identified 8 precursor miRNAs in MIR165/166 family, 6 in MIR167 and MIR7983, 3 in MIR7990, 2 in MIR8011 and 1 in MIR393, MIR530, MIR827, MIR828 and MIR8025 families of *S*. *lycopersicum* (Supplementary Table S5). In turn, in *S*. *pennellii* we identified 7 pre-miRNAs in MIR7983 family, 6 in MIR165/166, 5 in MIR167, 3 in MIR8011, 2 in MIR7990 and 1 precursor sequence in MIR8025, MIR393, MIR530, MIR827 and MIR828 (Supplementary Table S6). We also identified mature miRNAs in these families, including 12 in MIR7983, 10 in MIR167 and MIR165/166, 3 mature sequences in MIR7990 and MIR8011, 2 in MIR393 and MIR827, in addition to 1 in each family of MIR530, MIR828 and MIR8025 of *S*. *lycopersicum*. In *S*. *pennellii*, 14 mature miRNAs were found in MIR7983, 9 in MIR167, 10 in MIR165/166, 5 in MIR8011, 2 in MIR393, MIR828, MIR827, MIR7990, MIR8025 and 1 sequence in MIR530.

**Figure 4:**
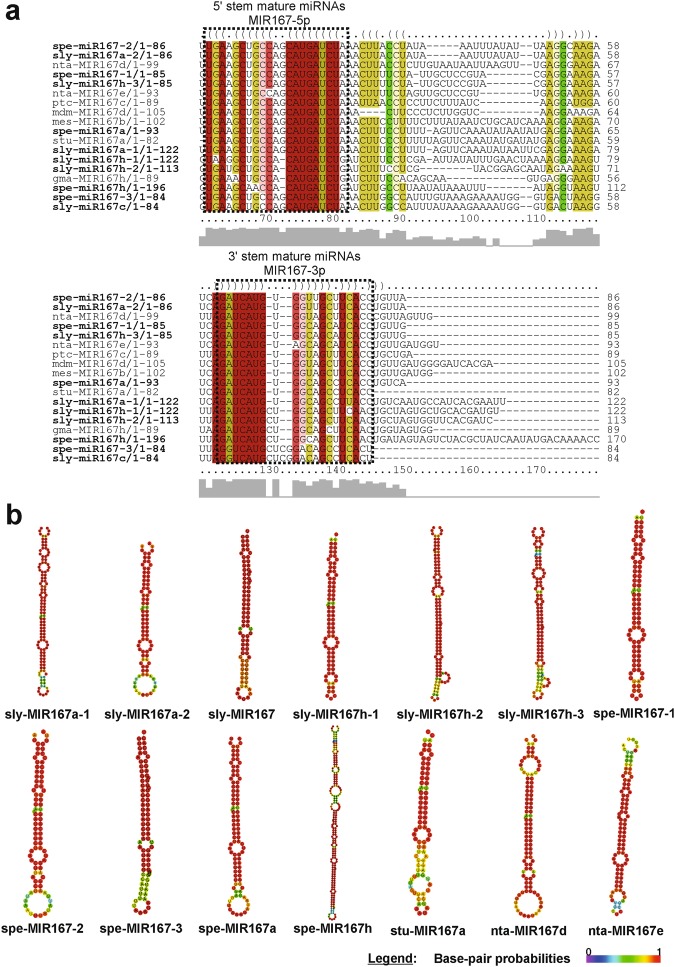
(**a**) The high-confidence RNA alignments between MIR167 families of *S*. *lycopersicum* and *S*. *pennellii* to their orthologues were performed using RNAalifold. Mature miRNAs are shown as boxes. Brackets and colours identify matching residues in 5′ and 3′ stems of hairpin structures. Levels of nucleotide identity are indicated below the alignment. (**b**) Secondary structures of MIR167 family from *S*. *lycopersicum*, *S*. *pennellii* and their orthologues were drawn by RNAfold. Sly - *Solanum lycopersicum*, Spe - *Solanum pennellii*, Nta - *Nicotiana tabacum*, Ptc - *Populus trichocarpa*, Mdm - *Malus domestica*, Mes - *Manihot esculenta*, Gma - *Glycine max*, Stu - *Solanum tuberosum*.

**Figure 5:**
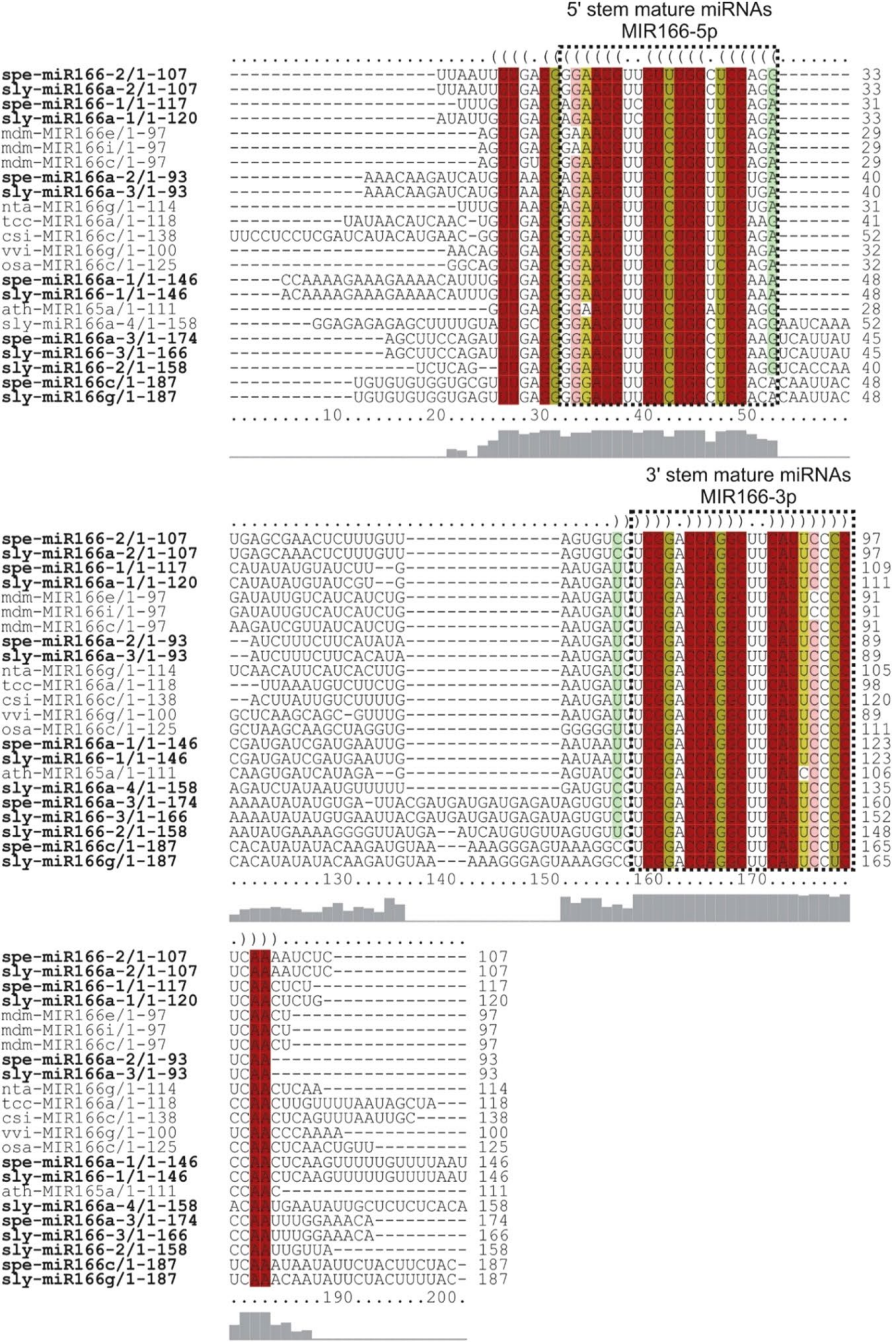
The high-confidence RNA alignments between MIR165/166 family of *S*. *lycopersicum* and *S*. *pennellii* to their orthologues were performed using RNAalifold. Mature miRNAs are shown as boxes. Brackets and colours identify matching residues in 5′ and 3′ stems of hairpin structures. Levels of nucleotide identity are indicated below the alignment. Sly - *Solanum lycopersicum*, Spe - *Solanum pennellii*, Mdm - *Malus domestica*, Nta - *Nicotiana tabacum*, Tcc - *Theobroma cacao*, Csi - *Citrus sinensis*, Vvi - *Vitis vinifera*, Osa - *Oryza sativa*, Ath - *Arabidopsis thaliana*.

The precursor and mature miRNAs identified in our work presented great conservation in their sequences and secondary structures in relation to their orthologues, especially in the miRNA mature region. This was the case for the Sly/SpeMIR167 (Fig. 4), Sly/SpeMIR165/166 (Fig. 5 and Supplementary Fig. S2), Sly/SpeMIR393 (Supplementary Fig. S3), Sly/SpeMIR530 (Supplementary Fig. S4), Sly/SpeMIR827 (Supplementary Fig. S5), Sly/SpeMIR828 (Supplementary Fig. S6), Sly/SpeMIR7983 (Supplementary Fig. S7), Sly/SpeMIR7990 (Supplementary Fig. S8), Sly/SpeMIR8011 (Supplementary Fig. S9) and Sly/SpeMIR8025 families (Supplementary Fig. S10).

**Figure 6:**
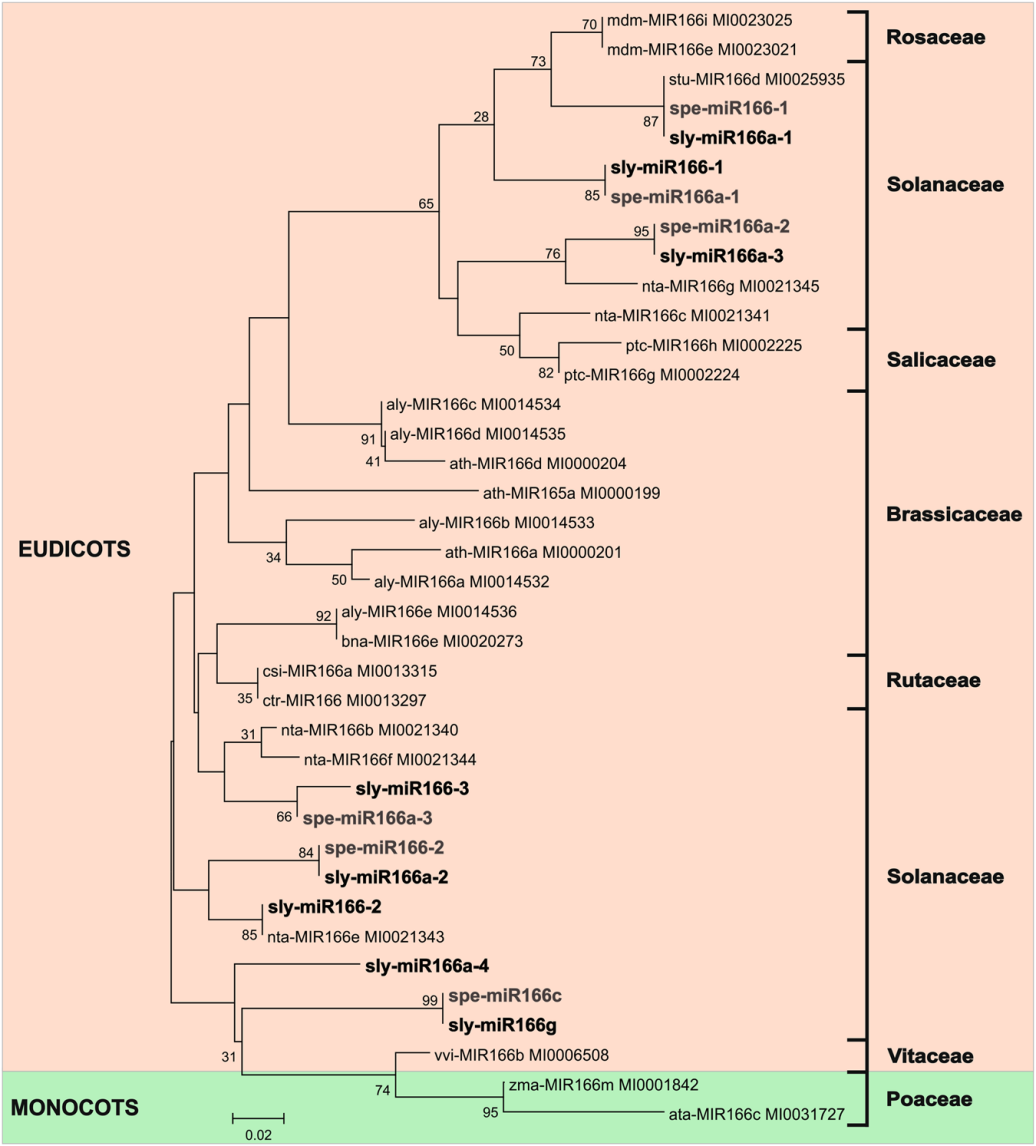
Phylogenetic tree of MIR165/166 family identified in *S. lycopersicum*, *S. pennellii* and their orthologs. Sly - Solanum lycopersicum, Spe - Solanum pennellii, Mdm - Malus domestica, Stu - Solanum tuberosum, Nta - Nicotiana tabacum, Ptc - Populus trichocarpa, Aly - Arabidopsis lyrata, Ath - Arabidopsis thaliana, Bna - Brassica napus, Csi - Citrus sinensis, Ctr - Citrus trifoliate, Vvi - Vitis vinifera, Zma - Zea mays, Ata - Aegilops tauschii.

The analysis of the MIR167 family showed a phylogenetic tree distributed in five distinct clades. The miRNAs from *S*. *lycopersicum* and *S*. *pennellii* were grouped together with miRNAs from *Solanaceae* species (Supplementary Figure S11). The phylogenetic tree of MIR165/166 family displayed two distinct clades of *Eudicot* and *Monocotyledon*. The miRNA sequences from both tomato species were distributed within *Solanaceae* in *Eudicot* clade (Fig. 6). In the phylogenetic trees of Sly/SpeMIR393 (Supplementary Fig. S12), Sly/SpeMIR530 (Supplementary Fig. S13) and Sly/SpeMIR827 (Supplementary Fig. S14), two clades of *Eudicots* and *Monocotyledons* were observed, as were miRNAs from tomato species within *Solanaceae* clade. The phylogenetic tree of Sly/SpeMIR828 family showed ten clades of different families of *Eudicots* (Supplementary Fig. S15).

#### miRNA target genes of *S*. *lycopersicum* and *S*. *pennellii*

To understand the putative roles of miRNAs in tomato, we identified their target genes. We identified 2471 different miRNA target genes from a total of 4197 in *S*. *lycopersicum* for 343 mature miRNAs (Supplementary Table S12) and 3462 different targets from a total of 5768 in *S*. *pennellii* for 388 mature miRNAs (Supplementary Table S13).

Notably, the Sly/SpeMIR167 family miRNAs showed 247 different target transcripts, 37 of which belonged to SlyMIR167 and 210 to SpeMIR167 families. This major difference in target numbers for the same miRNA family in wild tomato compared with *S*. *lycopersicum* can be explained by the domestication and improvement of the cultivars, providing increasing similarities of miRNA–target pairs in cultivated plants compared to those in wild plants, similar to the trend observed in soybeans^92^. Among these targets are: putative zinc-finger in N-recognin (UBR box), retrotransposon gag proteins, magnesium transporter NIPA, annexin p34, BZIP transcription factor, cytochrome P450, RING/U-box superfamily protein, kinetochore protein and a proteins class related to the disease resistance (CC-NBS-LRR). In addition, we identified 74 distinct targets in the Sly/SpeMIR165/166 family, with 35 of the SlyMIR165/166 and 39 of the SpeMIR165/166 families. Their targets constitute the homeobox-leucine zipper family, especially those with the MEKHLA domain, acyl-CoA N-acyltransferases (NAT) superfamily protein and retrotransposon gag protein. Interestingly, the Sly/SpeMIR393 family miRNA targets included 23 distinct transcripts, with 12 of the SlyMIR393 and 11 of SpeMIR393 family. These target genes encode carbohydrate-binding protein of the ER, lipoxygenases and proteins containing the U-box and F-box domains, such as transport inhibitor response 1 (TIR1) and an auxin receptor involved in a hormone-depleting mechanism. The Sly/SpeMIR530 family displayed 31 distinct targets, with 14 of the SlyMIR530 and 17 of SpeMIR530 family. Among the targets were the DEAD/DEAH box helicase, ICP0-binding domain of ubiquitin-specific protease 7, ethylene-responsive transcription factor, adenylate isopentenyltransferase, proteins containing the AP2 domain, NAC domain protein, MULE transposase domain and tyrosine kinase type proteins. The Sly/SpeMIR827 family miRNAs had 50 different mRNAs targets, with 29 of the SlyMIR827 and 21 of SpeMIR827 family. These miRNAs comprise reverse transcriptase (RNA-dependent DNA polymerase), cytochrome P450, OTU-like cysteine protease, no apical meristem (NAM) protein, cell-wall invertase, glyoxylate reductase and SPX domain-containing family protein.

The search for the Sly/SpeMIR828 targets, revealed 31 different genes, with 16 target genes of the SlyMIR828 and 15 of SpeMIR828 family. Among them were MYB-type DNA-binding domain, syntaxin of plants 51, F9H3-4 protein, TCP transcription factor 1 and reverse transcriptase (RNA-dependent DNA polymerase). The tomato Sly/SpeMIR7983 family showed 89 target genes, with 75 of the SlyMIR7983 and 14 of SpeMIR7983 family. The targets contain transcripts encoding 2C phosphatases, serine carboxypeptidase, ribonuclease T2 family, glutathione S-transferase-like protein, the ENTH/ANTH/VHS superfamily protein, alpha/beta-hydrolase superfamily protein, UDP-glucuronate 4-epimerase 4 and Myb family transcription factor family protein. In addition, Sly/SpeMIR7990 miRNAs showed 15 different target transcripts, with 13 of the SlyMIR7990 and 2 of SpeMIR7990 family, such as GDSL esterase/lipase proteins, MADS-box transcription factor family protein, disease resistance protein (TIR-NBS-LRR class), WRKY DNA-binding protein 31, Nop53 protein, bHLH transcription factor GBOF-1 and the permease family. Interestingly, we identified 55 distinct target genes from miRNAs belonging to the Sly/SpeMIR8011 family, with 27 of the SlyMIR8011 and 28 of SpeMIR8011 family. Among them were alpha/beta-hydrolase family proteins, hAT family C-terminal dimerization region, reverse transcriptase (RNA-dependent DNA polymerase), PHD-finger transcription factor, potassium transporter, ripening regulated protein and ARM repeat superfamily protein. Finally, the Sly/SpeMIR8025 family had 11 miRNAs targets, with 5 of the SlyMIR8025 and 6 of SpeMIR8025 family, which encode auxin response factor 9A (ARF9A), kinase protein domain, FKBP-type peptidyl-prolyl cis-trans isomerase, NAD(P)-binding Rossmann-fold superfamily protein and telomerase activating protein Est1 (Supplementary Tables S12 and S13).

#### Expression of tomato miRNAs in *S*. *lycopersicum* and *S*. *pennellii*

**Figure 7:**
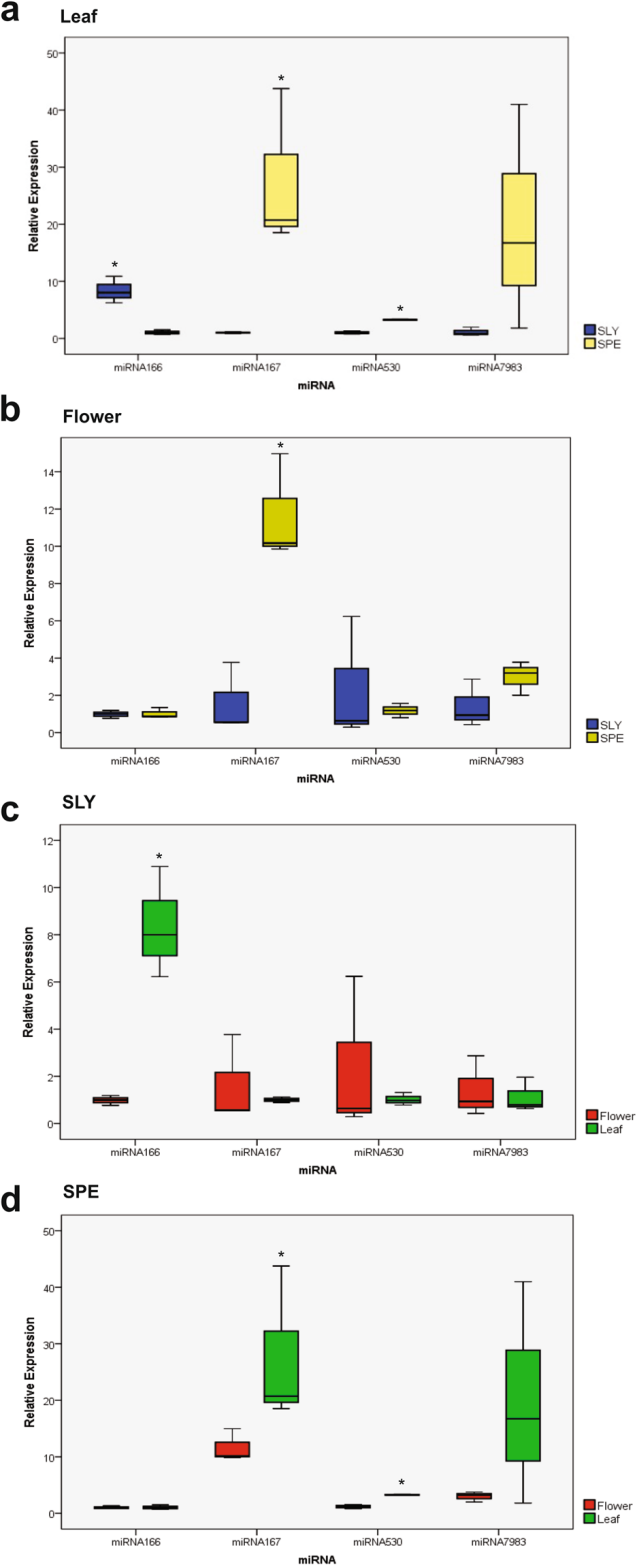
Relative expression profiles of miR165/166, miR167, miR530 and miR7983 in (**a**) leaves and (**b**) flowers of (**c**) *S*. *lycopersicum* (SLY) and (**d**) *S*. *pennellii* (SPE).

We experimentally verified the expression of four miRNA genes (miR165/166, miR167, miR530 and miR7983) in two different tissues: leaves and flowers of each tomato species. In leaves, the miR165/166 expression level was 7.3 times higher in *S*. *lycopersicum* than in *S*. *pennellii*. The miR167 and miR530 were expressed at levels, respectively, 26.7 and 2.2 times higher in leaves of *S*. *pennellii* than in *S*. *lycopersicum* (Fig. 7a). In tomato flowers, miR167 also showed a 10.0 times higher expression in *S*. *pennellii* than in *S*. *lycopersicum*, and miR165/166, miR530 and miR7983 had no significant difference in the expression levels between the two tomato species (Fig. 7b). In addition, the miR165/166 expression level was 7.4 times higher in leaves than in flowers, whereas miR167, miR530 and miR7983 showed no difference in the two *S*. *lycopersicum* tissues (Fig. 7c). Finally, the miR167 and miR530 had, respectively, 16.0 and 2.1 times higher expression levels in leaves than in *S*. *pennellii* flowers, but miR165/166 and miR7983 showed no difference in the two *S*. *pennellii* tissues (Fig. 7d).

#### Transcriptomic changes in tomato leaves and flowers RNA-seq-based

**Figure 8:**
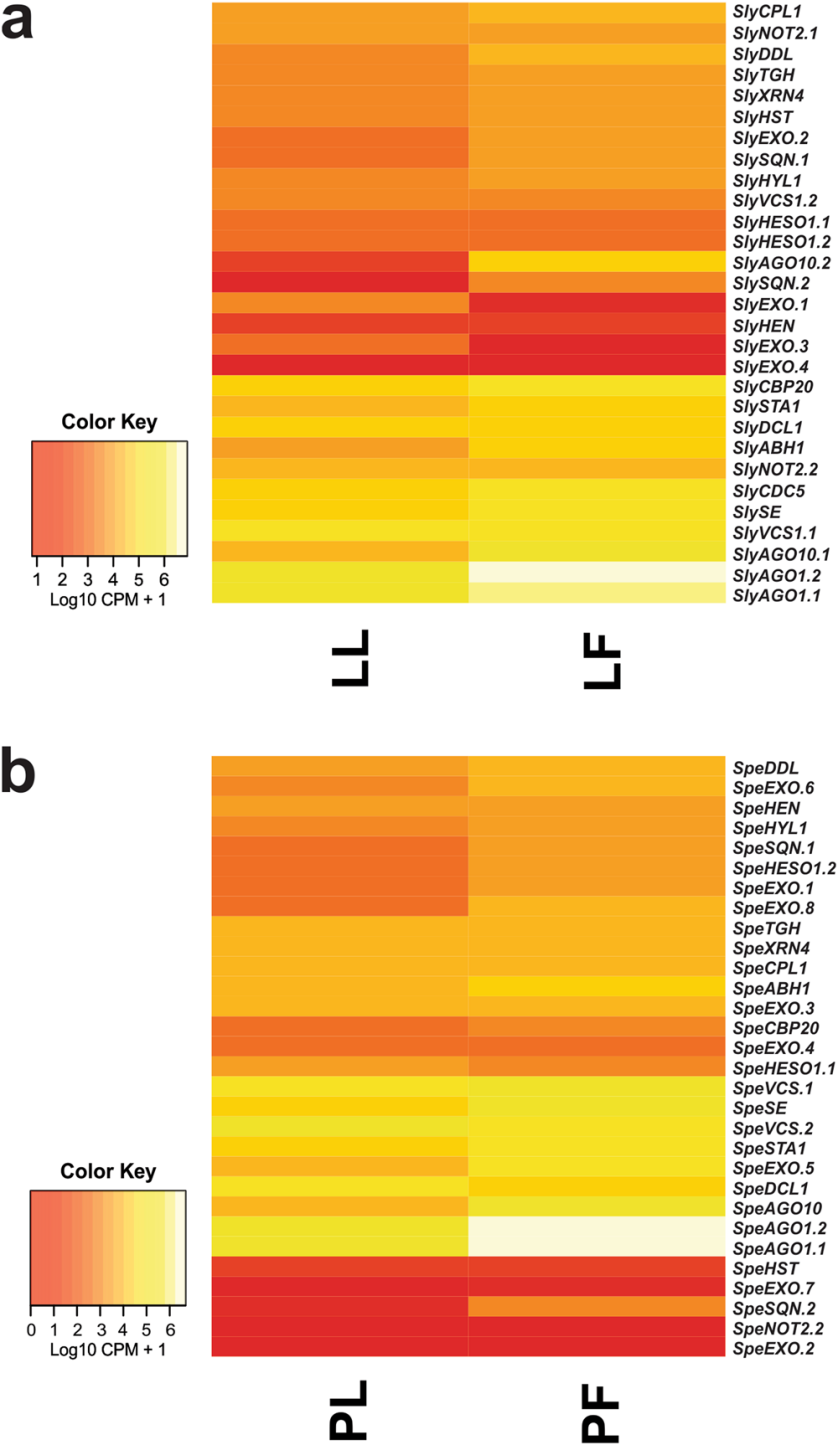
Heatmap analysis of the genes involved in the tomato miRNA pathway from leaves and flowers RNA-seq libraries. Heatmap shows the expression profile of the (**a**) *S*. *lycopersicum* and (**b**) *S*. *pennellii* genes in two tissues, being LL (*S*. *lycopersicum* leaves), LF (*S*. *lycopersicum* flowers), PL (*S*. *pennellii* leaves) and PF (*S*. *pennellii* flowers).

To confirm the expression of the RNA-silencing pathway components, we searched the RNA-seq data of *S*. *lycopersicum* and *S*. *pennellii* publicly available in the Sequence Read Archive (SRA)^93^. The heatmap confirmed expression of all genes identified in the two species in the two analysed tissues, except the *SpeEXO*.*2* gene that did not express in leaves (Fig. 8, Supplementary Figs S16, S17 and Table S14). In *S*. *lycopersicum* 10 of the 29 genes (*ABH1*, *AGO1*.*1*, *AGO1*.*2*, *AGO10*.*1*, *AGO10*.*2*, *EXO*.*1*, *EXO*.*2*, *EXO*.*3*, *SQN*.*1* and *SQN*.*2*) from the miRNA processing machinery were found to be differentially expressed (DE) between flowers and leaves. Among 10 DE genes, 8 were up-regulated in flowers and 2 were up-regulated in leaves, the latter being *SlyEXO*.*1* and *SlyEXO*.*3*. Considering the relative expression between *S*. *lycopersicum* flowers and leaves, *SlyAGO10*.*2* and *SlySQN*.*2* showed higher DE in flowers. When compared by absolute expression values (count per million - CPM), *SlyAGO1*.*2* was the most-expressed gene both in the flowers and leaves (Fig. 8a, Supplementary Fig. S16 and Table S14). Similar results were found in *S*. *pennellii*, wherein 11 of the 30 genes (*AGO1*.*1*, *AGO1*.*2*, *AGO10*, *DDL*, *EXO*.*1*, *EXO*.*5*, *EXO*.*6*, *EXO*.*8*, *HESO1*.*2*, *SE* and *SQN*.*2*) identified from the same pathway were found to be DE between flowers and leaves. In this case, all 11 genes were up-regulated in flowers, with *SpeSQN*.*2* and *SpeAGO10* genes more DE in flowers than in leaves. Regarding the absolute expression values, *SpeAGO1*.*2* and *SpeAGO1*.*1* were the genes most-expressed in flowers and leaves, respectively (Fig. 8b, Supplementary Fig. S17 and Table S14).

#### Validation of the tomato miRNAs in *S*. *lycopersicum* small RNAs libraries

To validate the miRNAs identified in this study, we retrieved 95 small RNA libraries of *S*. *lycopersicum* available in the SRA and verified the presence of mature miRNAs in each library (Supplementary Table S15). A robust analysis of the presence of 285 different sequences of mature miRNAs found in *S*. *lycopersicum* was performed in the 95 different small RNA libraries, in order to validate the miRNAs identified in this study. Among the 285 different mature miRNA sequences, 262 were found in at least one analysed library and 23 mature miRNAs were not found in the searched libraries. Members of the MIR165/166, MIR167, MIR393, MIR530, MIR827, MIR828, MIR7983, MIR7990, MIR8011 and MIR8025 families were found in 69.5%, 64.2%, 43.2%, 95.8%, 27.4%, 14.7%, 62.1%, 31.6%, 54.7% and 1% of the analysed libraries, respectively. Only one mature miRNA of the MIR7983 and MIR8025 families was not found in any studied library.

## Discussion

To better understand the biology, evolution and domestication and accelerate the agricultural applications of the miRNAs, the genes of their biogenesis pathway and their targets in tomato, we undertook the genomic study of these molecules in the cultivated tomato *S*. *Lycopersicum* and the wild tomato *S*. *pennellii*. Our results suggest that, in general, the miRNAs demonstrated similar evolutionary patterns between the two species studied. However, a larger number of molecules (miRNA processing pathways genes, their mature molecules and target genes) and higher levels of miRNA expression were found in the wild tomato than in the cultivated tomato. This finding showed a possible loss of genes along the evolution and domestication, which may be associated with higher biotic and abiotic resistance found in wild tomato, compared to cultivated tomato.

We identified 29 and 32 putative proteins involved in the miRNA pathways in *S*. *lycopersicum* and *S*. *pennellii*, respectively (Supplementary Tables S1 and S2), which is in agreement with the description of the miRNA biogenesis pathway in other plant species, such as in *A*. *thaliana*, *P*. *vulgaris* and *O*. *sativa*^43,91,94,95^. The presence of these proteins in different studies has shown a high conservation rate of the components of the miRNA processing pathways in plants, performing essential functions in the post-transcriptional regulation of mRNAs in different cells^43,91^.

In fact, our analysis revealed that the components of this pathway were highly conserved in the two species at the amino acid level, as well as the distribution of domains, active site location and phylogenetic distribution compared to orthologous proteins in other plant species. This pattern was observed in both protein families, ARGONAUTE and DICER, both considered to be critical in the processing machinery of miRNAs in plants^47,91^.

Among all proteins found, 15 and 16 proteins belong to the AGO family of *S*. *lycopersicum* and *S*. *pennellii*, respectively, compared to 19 identified in *O*. *sativa*^48^, 17 in *P*. *vulgaris*^95^. In *A*. *thaliana* 10 AGO proteins have been found^91,94^. Mirzaei *et al*.^70^ and Bai *et al*.^69^ found also 15 AGO proteins in *S*. *lycopersicum*, all of which were identified in this study^69,70^. However, in the study by Bai *et al*.^69^, two classifications were different. Solyc02g069280 and Solyc03g111760 were considered by the authors to be SlyAGO3 and SlyAGO15, but in our analyses they were classified as SLyAGO2 and SlyAGO4, respectively. Of all the AGO proteins identified in plants, only AGO1 and AGO10 showed activity in the biogenesis of miRNAs^91,94^.

The conserved domains found in SlyAGO1.1/1.2, SpeAGO1.1/1.2, SlyAGO10.1/10.2 and SpeAGO10 (Supplementary Fig. S1 and Table S3) were the same as in proteins from orthologous plant species, and the domain distribution was similar as well^96–99^. The Piwi conserved domains of the AGO proteins had an active site containing a DDH catalytic triad (Fig. 1a), present in the AGO protein family and proteins with RNase H function^48,94^. These results conformed to AGO1 and AGO10 proteins found in *A*. *thaliana* and *P*. *vulgaris*^48,94,95^. The conserved domains and active sites found in Sly/SpeAGO1 and Sly/SpeAGO10 showed high conservation compared to AGO1 and AGO10 proteins in *A*. *thaliana*, *O*. *sativa*, *S*. *tuberosum*, *G*. *Max* and *P*. *vulgaris*^48,95,98,100^. Such high domain conservation may be directly related to the role played by AGO1 and AGO10 proteins in the miRNA processing pathway in different species.

Other important catalytic components of the miRNA processing in plants are the DCL proteins^95,101–107^. In this study, we identified 8 and 11 putative DCL proteins of *S*. *lycopersicum* and *S*. *pennellii*, respectively. The number of DCL proteins found in tomato genome is in agreement with other plant species: 4 DCLs have been found in the *A*. *thaliana* genome^91,105^, 6 in *P*. *vulgaris*^95^ and in *O*. *sativa* at least 5 DCL proteins were identified^48,105,108^. Bai *et al*.^69^ and Wang *et al*.^71^ found only 7 DCL proteins in *S*. *lycopersicum*^69,71^.

The DCL1 protein is the protein of the DCL family involved in the processing of miRNAs^58,109,110^. The conserved domains found in Sly/SpeDCL1 (Supplementary Fig. S1 and Table S4) were very similar to their orthologous proteins from plant species^95,105,108^, suggesting a conserved function of DCL1 proteins in tomato species. According to recent studies, especially in DCL proteins, the distance between the RNAse III and PAZ conserved domains is suggested to be the largest determinant of the resulting length of processed miRNAs^51,105,111^. This demonstrates the importance of the domain distribution and conservation in the performance of these proteins at the cellular level. The two RNAse III domains (RNase IIIa and RNase IIIb) found in Sly/SpeDCL1 proteins displayed an active site with the EDDE catalytic residues (Fig. 1b), also identified in DCL1 proteins from other plant species^112^. These RNAse III domains are required for the cleavage and processing function of dsRNAs (double-stranded RNA) in the miRNA pathway in plants^105,108^.

A phylogenetic analysis of the Sly/SpeAGO (Fig. 2) and Sly/SpeDCL (Fig. 3) proteins was performed from their amino acid sequences, aiming at understanding the evolutionary diversification of AGO and DCL proteins in the two tomato species. The distribution of the seven clades showed a relationship between some paralogous proteins specific to the AGO family, with a great similarity among the AGO1, AGO5 and AGO10 proteins; AGO2 and AGO7; AGO6 and AGO4 conforming to the phylogenetic distribution in A. *thaliana* and *P*. *vulgaris*^91,95,113^. In general, the identified phylogeny distribution of AGO proteins is consistent with current species protein phylogenies^114^, which suggests that the conservation level for the members of the AGO family in *Angiosperms* is high. The phylogenetic tree of the AGO proteins showed a strong correlation between AGO1 and AGO10 proteins, with both AGO proteins participating in the miRNA pathway in plants^91,94^. AGO proteins have a key role in transcriptional and post-transcriptional silencing mediated by small RNAs in plants. The role of these proteins in miRNA regulation has been associated with the RISC complex (RNA-induced silencing complex), allowing degradation or inhibition of mRNA translation derived from the base complementarity between the miRNA specific sequences and the target mRNA^91,94,115^.

The analysis of Sly/SpeDCL proteins showed a great similarity to the evolutionary tree of life of these species within each clade^114^. The distribution among the DCL family paralogs showed a strong correlation between the DCL2 and DCL4 proteins, which were also close to DCL1 but further away from DCL3, consistent with the distribution in DCL families in other plant species^48,105^. Thus, it was observed that DCL proteins of *S*. *lycopersicum* and *S*. *pennellii* are highly conserved in the same way as observed for the AGO proteins, highlighting their great importance in post-transcriptional silencing processes in plants.

The validation of the miRNA pathway genes in the libraries available in the SRA^93^ showed the DE of 10 and 11 of the 29 and 32 genes identified in *S*. *lycopersicum* and *S*. *pennellii*, respectively (Fig. 8, Supplementary Figs S16, S17 and Table S14). Among the DE genes in the two species are the genes of *ABH1*, *AGO1*, *AGO10*, *DDL*, *EXO*, *HESO1*, *SE* and *SQN* families. In *S*. *lycopersicum*, of the 10 DE genes, 8 were up-regulated in flowers and 2 up-regulated in leaves, and in *S*. *pennellii* all 11 DE genes were up-regulated in flowers. This result shows a greater requirement of these genes in flowers, suggesting an essential role of the miRNA in this tissue, and considering that several miRNAs targets are related to different roles in flowers^116–118^. Yamaguchi and Abe (2012) and Wang^117^ showed an important function of miR172, miR156 and miR159 in the flowering time control in *A*. *thaliana*^117,118^. In addition, Aukerman and Sakai (2003) demonstrated that miR172 causes early flowering and disrupts the specification of floral organism when overexpressed in *A*. *thaliana*^116^.

Only the *SlyEXO*.*1* and *SlyEXO*.*3* genes were up-regulated in leaves compared with flowers, whereas the *SpeEXO*.*2* gene was not expressed (Fig. 8, Supplementary Figs S16, S17 and Table S14) because these genes were also involved in other pathways or have other functions, such as delivery of mRNAs, miRNAs and cell-specific proteins, considering that this family had the greatest number of paralogous sequences^119,120^. In both species, *AGO10* and *SQN*.*2* were the most up-regulated genes in flowers, suggesting that the mechanism of using these genes in flowers is conserved in these two species. Ji *et al*.^121^ showed overlapping roles of *AGO10* and *AGO1* in the temporal regulation of floral stem cells^121^. Furthermore, Liu *et al*. (2014) reported that the role of miR168a in sweet orange was the accumulation of *AGO1* in leaves and fruits^122^, which may explain up-regulation of *AGO10* and *SNQ*.*2* in the leaves, considering that *AGO10* has important roles in this tissue, and SQN has a direct relation with *AGO1*, whereas *AGO1* has also important roles in flowers and is down-regulated by miR168 in leaves^121,122^. Considering the absolute expression values (CPM), *Sly/SpeAGO1*.*2* and *SpeAGO1*.*1* were the most-expressed genes in both tissues, showing the great importance of this protein family in plant development and, consequently, in the post-transcriptional silencing machinery using miRNAs^123^.

As for the miRNA sequences, we identified 343 and 388 mature miRNAs, 226 and 234 precursor miRNAs, distributed in 87 and 85 distinct miRNA families, for 4197 and 5768 target genes in the *S*. *lycopersicum* (Supplementary Table S5) and *S*. *pennellii* (Supplementary Table S6) genomes, respectively. In addition to predicting previously reported miRNAs, our study identified many additional novel miRNAs in the genomic sequences of *S*. *lycopersicum*; notably, of the all miRNAs found, only 110 mature and 77 precursors were deposited in the miRBase (version 21)^124^ (Supplementary Table S5). Thus, we identified 233 new mature miRNAs and 149 new precursors contained in 41 miRNAs families in comparison with miRNAs deposited in miRBase.

Some other studies searched for miRNAs in *S*. *lycopersicum*, such as Moxon *et al*.^77^ who found only 30 miRNAs, whereas Yin *et al*.^74^, Kim *et al*.^7^, Din and Barozai^80^, Luan, Wang and Liu^125^ and Fan *et al*.^73^ detected 21, 12, 109, 14 and 218 miRNAs, respectively^7,73,74,80,125^. In addition, other works found only six miRNAs responsive to long-term RPM-treatment^87^, 69 miRNAs families linked with phosphate and mycorrhizal signalling^81^, 112 mature miRNAs responsive to curl *New Delhi virus* (ToLCNDV)^75^, 143 miRNAs associated with *Botrytis cinerea* infection^82^, 95 miRNAs responsive to salt-induced stress^78^, and only 69 putative miRNAs associated with biotic stress^79^. Further, our study identified 114 new mature miRNAs, 88 new precursors contained in 28 miRNAs families of the *S*. *lycopersicum* in comparison with studies published to date^7,73–75,77,80,87,125–133^. Moreover, we identified for the first time mature miRNAs and their precursors in *S*. *pennellii* (Supplementary Table S6).

The miRNA precursor gene localization and distribution in *S*. *lycopersicum* and *S*. *pennellii* genomes showed that 86.73% and 81.59% of the genes were found in intergenic regions, respectively, and that, in *S*. *pennellii*, 3 of them (spe-miR169-1, spe-miR1919 and spe-miR5368) are located in more than one position in the genome (Supplementary Table S7). Such high percentage of intergenic miRNAs was also found in other plants, such as *A*. *thaliana*, *P*. *trichocarpa*, *O*. *sativa*, *P*. *vulgaris*, *Sorghum bicolour* and *Catharanthus roseus*^37,95,96,134–137^. Some of the miRNA genes identified were organized in clusters (72 in *S*. *lycopersicum* and 73 in *S*. *pennellii*). The presence of miRNA genes distributed in clusters in other plants has been reported before, e.g., 54 miRNAs in clusters were found in *A*. *thaliana*, 40 in *P*. *trichocarpa*, 103 in *O*. *sativa*, 32 in *S*. *bicolour*^137^ and 25 in *P*. *vulgaris*^95^. Furthermore, 50 miRNA genes in *S*. *lycopersicum* and 47 in *S*. *pennellii* were also organized in clusters but in antiparallel strands, in agreement with other plant genomes deposited in the miRBase (version 21), such as miR399a and miR399e from *O*. *sativa*, miR169e and miR169d from *A*. *thaliana* and miR167e and miR167i from *G*. *max*^124^.

The structural and thermodynamic characteristics of the *S*. *lycopersicum* and *S*. *pennellii* were similar to those reported in other plant studies^127,138–140^. In plants, the values for MFE in precursor miRNAs are typically smaller than −18 kcal/mol^140^. Furthermore, studies have shown that the sequences of RNAs with mean AMFE −45.93 ± 9.43 kcal/mol are considered real miRNAs, based on the knowledge that AMFEs found in miRNA precursors had higher negative values than in other classes of non-coding RNAs, for example, tRNAs and rRNAs. MFEIs with a mean value of 0.97, above 0.85 were suggested for potential precursors of plant miRNAs, which distinguished them from other RNAs, consistent with our results^138^.

The statistical analyses using thermodynamic and structural characteristics of the tomato pre-miRNAs, when comparing the two tomato species (Supplementary Table S10) and *Solanaceae* family (Supplementary Table S11) showed no differences between the groups (*p* < 0.05). Both displayed high conservation and similarity among pre-miRNAs from *S*. *lycopersicum*, *S*. *pennellii* and also species belonging to the *Solanaceae* family. However, the results for *S*. *lycopersicum* and *S*. *pennellii* were significantly different from results for *Fabaceae* and *Brassicaceae* families (*p* < 0.05), suggesting a great evolutionary conservation of the pre-miRNA characteristics within each plant family and a significant difference among different families (Supplementary Table S11). These differences with respect to pre-miRNA characteristics were also observed for *Monocotyledons* and *Eudicotyledons*^141^.

Several studies have shown that the MIR165/166 and MIR167 families are highly conserved in different plant species, such as *A*. *thaliana*, *O*. *sativa*, *G*. *max*, *P*. *vulgaris* and *S*. *tuberosum*^95,127,142–146^. Members of the MIR393 family were also found in *Ferns*, *Gymnosperms*, *Angiosperms*, *Monocotyledons* and *Eudicots*, such as *A*. *thaliana* and *P*. *vulgaris*^47,56,95,109,127,147–150^. The MIR530 family was also identified in orthologous plants, such as *S*. *tuberosum*, *O*. *sativa*, *C*. *sinensis*, *P*. *vulgaris* and *Z*. *mays*^95,106,127,148,151,152^. Furthermore, *P*. *trichocarpa*, *O*. *sativa*, *Z*. *mays*, *S*. *tuberosum*, *A*. *thaliana* and *Brassica spp*. also possess miRNAs of the MIR827 family in their genomes^127,149,153–155^, and studies have demonstrated the presence of miRNAs of the MIR828 family in other species, such as *A*. *thaliana*, *O*. *sativa*, *P*. *vulgaris*, *V*. *vinifera* and *Z*. *mays*^95,148,150,156–159^. On the other hand, the MIR7990 and MIR8025 families were identified in *S*. *tuberosum*^124,155^, MIR7983 in *Solanum pimpinellifolium*^160^, and to date, the MIR8011 family has been found in *Lycium chinense*^152^. In addition MIR7990, MIR8011 and MIR8025 families were identified for the first time in *S*. *lycopersicum* and *S*. *pennellii* in this study suggesting that these family can be restricted to *Solanaceae* (solanum-specific)^155^.

The phylogenetic analysis performed of the MIR167 (Supplementary Fig. S11), MIR165/166 (Fig. 6), MIR393 (Supplementary Fig. S12), MIR530 (Supplementary Fig. S13), MIR827 (Supplementary Fig. S14) and MIR828 miRNAs families (Supplementary Fig. S15) of *S*. *lycopersicum* and *S*. *pennellii* highlighted an evolutionary distribution that highly conforms to the accepted tree of life for plant species^161^.

Several tomato miRNAs showed functions modulating important biological processes through the gene expression control (Supplementary Tables S12 and S13). The targets of Sly/SpeMIR165/166 family are involved mainly in plant vascular development^162^, including the formation of axillary meristems, root lateral meristems, laminar outgrowth and differentiation in leaves, stems, and roots^143,163–167^. Several studies have also identified targets of miR165/166 in *A*. *thaliana* and *O*. *sativa*, in agreement with our results, also showing a great conservation of function in this family of plant miRNAs, such as in retrotransposon, homeobox-leucine zipper family and kinase family proteins^99,144,163–165,168,169^.

The miR165/166 gene expression by real-time PCR in leaves and flowers of the two studied species showed interesting differences (Fig. 7). The miR165/166 was expressed at a higher level in *S*. *lycopersicum* leaves than in *S*. *pennellii* leaves. Specifically, in *S*. *lycopersicum*, miR165/166 has been expressed at a higher level in leaves than in flowers. The predicted targets for the tomato miR165/166 families have been related to the HD-Zip protein families, which participate in secondary development and vascular differentiation. In this sense, miR165/166 in *S*. *lycopersicum* leaves displayed overexpression compared to *S*. *pennellii* leaves suggesting a higher level of regulation of vascular development in the cultivated tomato species leaves than in the wild species. miR165/166 also showed high expression in rice after a short-term heat stress^170^, potato^155^ and in leaves, stems and roots of *Poncirus trifoliate*^171^. miRNAs from this family were also associated with a phenotype diversity involved in drought tolerance^172–174^. Furthermore, Guo *et al*.^175^ showed that during the establishment of Chinese kale seedlings, the miR165/166 expression was also regulated, showing its relation to the normal leaf development^175^, consistent with the higher expression found in leaves of *S*. *lycopersicum* than in flowers.

Many reports in plants have demonstrated conserved targets for MIR167 family, such as the genes involved in the disease resistance (CC-NBS-LRR) in *O*. *sativa* species and genes of the peptidase family proteins in *A*. *thaliana*^169^ consistent with the Spe/SlyMIR167 targets found in our study. Other important targets of MIR167 family include genes involved in the regulation of auxin response factors (ARF4, ARF6 and ARF8)^176–181^. As for expression, the miR167 was expressed at a higher level in *S*. *pennellii* than in *S*. *lycopersicum*. When comparing the *S*. *pennellii* tissues, leaves showed higher expression than flowers. In rice seeds^170^ and in *S*. *tuberosum* leaves and flowers^182^, miR167 was more abundant than in other tissues. Unlike *Citrus grandis*, which showed low expression of the miR167 family members in the floral developmental stages, indicating an important role in the reproductive development^183^ in agreement with our findings of low expression in flowers. In addition, miR167 was shown to be responsive to heat shock in *Arabidopsis*^184^ and played an important role in nitrogen stress responses, being up-regulated in maize shoots and roots^185^. Furthermore, most of the observed miR167 targets in plants were related to the hormonal pathways regulation, as in *A*. *thaliana*, rice and coffee^154,186,187^. Considering that miR167 plays these important roles, such as responses to heat shock, nitrogen stress and hormonal pathway regulation, its higher expression in *S*. *pennellii* than in *S*. *lycopersicum* suggests a significant influence on the metabolic pathway involved in resistance of wild plants to these abiotic stresses.

Interestingly, the Sly/SpeMIR393 family affects important proteins that regulate hormone-sensitive signalling in plant development^188–192^. The targets found for Sly/SpeMIR393, mainly the TIR1 genes, are in line with the data reported for *A*. *thaliana*, *O*. *sativa*, *Z*. *mays*, *P*. *vulgaris*, *Hordeum vulgare*, *Gossypium hirsutum* and *M*. *truncatula*^43,56,95,127,149,169,188–190,193–195^. In barley, miR393 acts as an integrator of environmental cues in auxin signalling, being able to improve plant resistance to aluminium toxicity^195^. In addition, miR393 exhibited important roles in nitrate response, defence against pathogenic bacteria and plant development^191^.

The Sly/SpeMIR530 family targets the genes encoding plus-3 domain-containing proteins, which was also found in *N*. *tabacum*^196^ and *O*. *sativa*^169^. Additionally, this miRNA family was involved in the nitrogen regulation and high salinity^197,198^, being pivotal in the plant physiological response to stress^199^. The higher miR530 expression in *S*. *pennellii* suggest an important role in the plant physiological control, mainly related to plant response to biotic and abiotic stresses, in which case it may need a higher expression in the wild species, according to their targets^200^.

Other studies also revealed similar targets for Sly/SpeMIR827 family miRNAs, such as SPX domain-containing family protein in *A*. *thaliana*^169^ and *O*. *sativa*^201^. The targets found indicated that miR827 plays a key role in plant stress adaptation, especially in nitrogen and phosphorus deficiency^202–205^. miR827 showed high expression in rice seeds^154^, unlike maize, which showed a suppressed expression in nitrogen deficiency, consistent with other studies that showed an involvement of miR827 in adaptive responses to low nitrogen and phosphorus conditions, showing its important role in stress adaptation^185,197,198,202,203^.

Sly/SpeMIR828 target genes showed its important roles in cell proliferation, control of apical dominance and organ symmetry, as well as senescence, such as found in studies carried out in other plants^159,202,206,207^. In *A*. *thaliana*, the MYB-type DNA-binding domain was also identified as miR828 target^169^. miR828 was expressed at higher levels in active bud in *Camellia sinensis*. The miR828 targets showed a role in regulating transcription and nucleotide metabolism^208^ and were holistically expressed in grapevine^209^. The miR828 presence also showed its likely involvement in phenylpropanoid metabolism, biotic and abiotic stress, cell differentiation and hormone responses^210^. Therefore, the targets found in this study showed the importance of the regulation performed by miRNAs (Supplementary Tables S12 and S13).

The validation of the mature miRNAs identified in this study, used 95 small RNAs libraries of *S*. *lycopersicum* available in the SRA (Supplementary Table S15). By performing a robust analysis, 262 mature miRNAs of *S*. *lycopersicum* were found in at least one library. This result showed that the methods used in this study for the miRNAs prediction are quite effective. Only 23 mature miRNAs were not found in the libraries. Among these miRNAs were sly-miR172d-4-5p, sly-miR172d-9-3p, sly-miR2111a-3p, sly-miR403a-3p, sly-miR7983-6-3p, sly-miR7992-5p, sly-miR8008b-2-3p and sly-miR8025-5p. Yet, other mature miRNA derived from their pre-miRNAs were present in the libraries, suggesting that in these specific libraries only one mature miRNA was necessarily expressed. Moreover, other mature miRNAs might be absent from the analysed libraries because some miRNAs are expressed only under specific conditions and/or tissues, which were not present in these libraries^211,212^. However, such validation analysis of the mature miRNAs could not be performed with *S*. *pennellii* due to the low number of small RNAs libraries available for this species, highlighting the fact that our study was the first to identify miRNAs in *S*. *pennellii*.

## Conclusion

Considering the global importance of the tomato culture and the problems generated by the susceptibility to the pests of the cultivated tomato (*S*. *lycopersicum*) and the differences with the wild tomato (*S*. *pennellii*), the obtained results elucidated several aspects of miRNAs in these two species. Our results expand the study of miRNAs in plants by providing a better understanding of their essential roles in the miRNA-based regulation processes in tomato, their processing pathways and gene expression, as well as providing targets for future investigations.

## Methods

### Identification of putative proteins involved in the miRNA pathway in *S*. *lycopersicum* and *S*. *pennellii*

The sequences of proteins involved in small RNA pathways in *A*. *thaliana* and *Solanum tuberosum* were identified and retrieved from the reference protein database (refseq-protein) available at NCBI (National Center for Biotechnology Information). These sequences were used as queries to search for putative proteins involved in the small RNA biogenesis (including miRNAs) in *S*. *lycopersicum* and *S*. *pennellii* using BLASTp (Basic Local Alignment Search Tool) (http://blast.ncbi.nlm.nih.gov/Blast.cgi)^213,214^. Using the reference sequences, the putative sRNA pathway proteins were searched for in the two tomato species using the *S*. *lycopersicum* (ITAG3.0) and *S*. *pennellii* (Spenn_v2.0) data of the Sol Genomics Network (https://solgenomics.net/)^28^.

### Prediction of mature and precursor miRNAs

Initially, the *S*. *lycopersicum* (ITAG3.0)^29^ and *S*. *pennellii* (Spenn_v2.0)^20^ assembly genome were obtained from Phytozome v12.1 (https://phytozome.jgi.doe.gov/)^215^ and PlaBi dataBase (http://www.plabipd.de/project_spenn/start.ep), respectively. Genome annotation files were also obtained from the databases for the respective species. The putative miRNAs and their precursors were searched applying the method described by De Souza Gomes *et al*.^216^, adapted for both species^216^. First, we obtained the genome sequences and found the potential hairpin sequences or similarity to miRNA precursor structures using Blastn (NCBI) and Einverted (EMBOSS tool) programs^217^ and the parameters of 336-nucleotides maximum repeat and threshold value of 25. Several filters were applied to these sequences to discard undesirable sequences, such as other non-coding RNAs sequences, and to keep those corresponding to putative miRNAs. The applied filters were based on conserved characteristics of precursor miRNA, as well as characteristics of other known regions, which had no potential to originate precursor miRNAs. These filters were: the GC (guanine and cytosine) content between 20% and 65%, minimum free energy (MFE), homology with conserved mature miRNAs, homology with repetitive regions and non-coding RNAs, except miRNAs^216^.

The miRBase database (version 21 - http://www.mirbase.org/)^124,218^ was used for comparisons with the putative hairpin sequences and find novel *S*. *lycopersicum* and *S*. *pennelli* miRNAs by accepting a maximum of 5 mismatches in final mature sequence. Other RNA groups, such as ribosomal RNA (rRNA), transporter RNA (tRNA), small nuclear RNA (snRNA), small nucleolar RNA (snoRNA), SL RNA, SRP, and RNase P were discarded based on the RFAM database version 12.0^219^ and repetitive sequences were discarded using the RepeatMasker 4.0.5 (http://www.repeatmasker.org/). The putative precursor and mature miRNAs identified were used for further analysis.

The pre-miRNA sequences identified in *S*. *lycopersicum* and *S*. *pennellii* were characterized according to their thermodynamic structures and parameters: minimum free energy (MFE), adjusted minimum free energy (AMFE), free minimum energy index (MFEI), size, content of A, content of U, content of C, content of G, of GC and AU, GC ratio, AU ratio, minimum free energy ensemble (MFEE), ensemble diversity (Diversity) and MFE structure frequency in the ensemble (Frequency). The AMFE was determined with MFE of 100 nucleotide-long sequence, and the MFEI was determined from the equation: MFEI = [(AMFE) × 100]/(G% + C%)]^129^. The pre-miRNA secondary structure prediction, in addition to the diversity calculation, MFE, ensemble frequency and MFE of the secondary structures were performed using the RNAfold (http://rna.tbi.univie.ac.at/cgi-bin/RNAWebSuite/RNAfold.cgi). The GC content and other structural properties were defined using Perl scripts.

### Analysis of conserved domains, active sites, phylogeny, primary and secondary alignments

For the conservation analyses of domains, active sites and structure evaluation, putative protein sequences and precursor miRNAs, we used the multiple alignment by ClustalX 2.1 and ClustalW^220^. In addition, we used RNAalifold (http://rna.tbi.univie.ac.at/cgi-bin/RNAWebSuite/RNAalifold.cgi) to analyse the primary and secondary structure alignments of precursor miRNAs with their respective orthologues^220^. Adjusted parameters (gap opening: 22.50; gap extension: 0.83) were used for the multiple alignments of precursor miRNA sequences^221^. The pre-miRNAs and proteins sequences logos were originated from WebLogo 2.8.2 (http://weblogo.berkeley.edu/logo.cgi)^222^.

The conserved protein domains were recovered separately to verify, through multiple alignment, the presence of important amino acid residues. In addition, the PFAM family protein database (http://pfam.xfam.org/)^223^ was used to find conserved domains and their putative functions. The Conserved Domain Database (CDD) (http://www.ncbi.nlm.nih.gov/Structure/cdd/wrpsb.cgi)^224^ was used to identify amino acids from the active sites of putative proteins.

The phylogenetic analysis was performed using MEGA5.2 program and the neighbour-joining method for miRNA precursor sequences and also putative proteins^225,226^. The miRNA evolutionary distance was calculated using the Kimura-2-parameter in site-replaced base units. For the putative proteins, the Jones-Taylor-Thornton (JTT) model was used^227^. A consensus tree was obtained using a bootstrap of 5000 replicates for the precursor miRNAs and 1000 replicates for the proteins, representing the evolutionary history of the analysed sequence group.

### miRNA target prediction

The miRNA targets identified in *S*. *lycopersicum* and *S*. *pennellii* were predicted using the psRNATarget tool (2017 Update) (http://plantgrn.noble.org/psRNATarget/)^228,229^. In order to obtain the lowest false-positive prediction rate for miRNA target genes, the threshold was strict, being 3.0 for “Maximum expectation (Exp)”. Other parameters were standard: “Length for complementarity scoring (HSP size)” −19 bp; “Top target genes for each small RNA” −200; “Target accessibility - allowed maximum energy to unpair target site (UPE)” −25; “Flanking length around target site for target accessibility analysis” −17 bp before/13 bp after; “Range of central mismatch leading to translational inhibition” −10 – 11 nucleotides. To analyse the miRNA target genes predicted in tomato, candidate targets were retrieved by Phytozome v12.1 and Sol Genomics Network databases.

### miRNA expression by real-time PCR

#### Plant material

The plant material was harvested at the “HortiAgro Sementes S/A” Experimental Station of the Center of Development and Transfer of Technology of the Federal University of Lavras (UFLA), at the municipality of Ijaci, MG. Leaves, flowers and fruits of *Solanum lycopersicum* and *Solanum pennellii* were harvested from three plants as biological replicates. The material was immediately frozen in liquid N_2_ and stored in 50-ml tubes in a −80 °C freezer until RNA extraction.

#### RNA extraction

Total RNA was extracted using the QIAzol^®^ Lysis Reagent (QIAGEN). Approximately 100 mg of tissue was ground in liquid N_2_ and transferred to a tube, to which 1 ml of QIAzol was added and homogenized by vortexing. The tubes were left at room temperature for five minutes. Afterwards, 300 µl of chloroform was added and homogenized by vortexing. The mixture was left at room temperature for five minutes. Afterwards, the tubes were centrifuged at 12000 g at 4 °C for 15 min. Three phases were formed, and 400 µl of the upper phase was collected and transferred to a new tube, to which 500 µl of isopropanol was added and homogenized by vortexing, and the tube was left at room temperature for 10 min. The tubes were then centrifuged at 12000 g at 4 °C for 10 min for precipitation of nucleic acids. The liquid was discarded, and the pellet was washed with 1 ml of 75% ethanol and centrifuged at 7500 g at 4 °C for 10 min, and the liquid was discarded carefully to preserve the pellet. Then, 500 µl of isopropanol was added again and the steps were repeated. The pellet was dried at room temperature and then resuspended in 20 µl of autoclaved mili-Q water.

The RNA integrity was visualized in 0.8% agarose gel, and the quantity and quality (ratio 260/280 and 260/230 between 1.8 and 2.2) were measured on Nanovue spectrophotometer.

#### DNAse treatment

Total extracted RNA was treated with the Turbo DNA-free^TM^ kit (Life Technologies^TM^). Five µg of RNA was used, and the corresponding volume was adjusted to 22 µl with autoclaved milli-Q water. Then, 2.5 µl of the 10x Turbo DNAse Buffer and 0.5 µl of the Turbo DNAse were added to each sample. The reaction was incubated for 30 min at 37 °C. Afterwards, to stop the DNAse enzyme activity, 2.5 µl of the DNAse Inactivation reagent was added. The samples were left at room temperature for 5 min, with occasional mixing, and then centrifuged at 10000 RPM for 2 min. After centrifugation, 15 µl were collected and transferred to a new tube. The samples were stored at −20 °C until cDNA synthesis.

#### cDNA synthesis and relative expression

The stem-loop method^63,230^ was used for cDNA synthesis and expression analysis, following the previously described steps^63,187^. In this method, three primers are needed – the stem-loop RT primer, the forward and the reverse primers. The stem-loop RT primers were designed according to Chen (2004)^230^. The sequence data are presented in Supplementary Table S15. The stem-loop primers contain 50 nucleotides, of which 44 nucleotides correspond to a universal sequence that forms a stable stem-loop structure at low temperatures. The last 6 nucleotides at the 3′ end of the stem-loop RT primer is a reverse complement of the last 6 nucleotides at the 3′ end of the specific miRNA. The forward primers were designed with the exact sequence of the miRNA, excluding the last 6 nucleotides at the 3′ end. To increase the melting temperature, nucleotides were randomly added (5–7 nucleotides) to the 5′ end of the primer. Primer design software was used to calculate the melting temperature and verify the quality of the forward primers. The third, reverse primer is a reverse complement of the universal sequence of the stem-loop RT primer, and, therefore, is used for every miRNA.

The cDNA synthesis from total RNA (DNA-free) was performed using the *ImProm-II*^*TM*^ *Reverse Transcriptase* (Promega). For each miRNA, 500 ng of RNA was used. The volume was adjusted to 7 µl with autoclaved mili-Q water. To the RNA, 1 µl of oligo-dT primer; 2 µl of specific stem-loop RT primer (1 µM) and 1 µl of the dNTP mix were added. The samples were incubated at 70 °C for 10 min for denaturation of the secondary structures and later incubated at 4 °C for 10 min. Then, 5 μl of Improm-II 5 × reaction buffer, 2.4 μl of 25 mM MgCl_2_, 0.6 μl of RNaseOut (Invitrogen), and 1 μl of the Improm-II Reverse Transcriptase were added. The reactions were incubated in a thermocycler at 16 °C for 30 min, followed by reverse transcription of 60 cycles at 30 °C for 30 s, 42 °C for 30 s, and 50 °C for 1 s. For inactivation of the Improm-II Reverse Transcriptase, the reaction was incubated at 70 °C for 15 min. Subsequently, the reactions were stored at −20 °C.

Quantitative real-time PCR (RT-qPCR) was performed using a standard SYBR Green PCR kit protocol on a Rotor-Gene Q (QIAGEN). The reactions were performed in a final volume of 15 μl, using 1.5 μl cDNA, 1.5 μl of each primer at a final concentration of 1 µM, 7.5 μl of SYBR Green PCR Master Mix (QIAGEN), and 3.0 μl of water, for each reaction. The reaction was incubated at 95 °C for 5 min, followed by 40 cycles at 95 °C for 5 s and 60 °C for 10 s. Then, the samples were heated from 55 to 95 °C with an increase of 1 °C to acquire the melting curve of the amplified products. All reactions were run in triplicate.

The primer efficiency was calculated for each miRNA and the reference genes by a standard curve of a 1:5 serial dilution of a pool of cDNA of the respective miRNA. The primer efficiencies were: miR165/166 (87%), sly-miR167 (97%), spe-miR167 (116%), miR7983 (97%) and miR530 (98%).

For the relative expression experiment of miRNAs from selected tissues, the 1:25 dilution was chosen. For the calculation of relative expression, the normalized comparative Cq (quantitative Cycle) method was used^64^, which takes into account the primer efficiency in the calculation. The normalization factor was the geometric mean of the U6 and 5.8S gene expression.

### Expression of miRNA pathway genes in tomato RNA-seq libraries

Paired-end reads from leaves and flowers of both wild (*Solanum pennellii*) and cultivated (*Solanum lycopersicum*) tomato were retrieved from the Sequence Read Archive (SRA) under accessions numbers SRR786556, SRR786557, SRR786570, SRR786571, SRR786552, SRR786553, SRR786566, SRR786567, SRR786524, SRR786525, SRR786540, SRR786541, SRR786542, SRR786520, SRR786521, SRR786535, SRR786536, SRR786537^93^. In this way, 10 libraries for *S*. *lycopersicum* and 8 libraries for *S*. *pennellii*, totalling 77354968 paired-end reads, were used for differential expression analyses.

The quality of the libraries was evaluated using FastQC software^231^. Adapters were identified using minion^232^ and removed with Trimmomatic^233^ as well as were the reads with the quality score and length below 20 and 35 bp, respectively. After the quality control, 71703462 paired-end reads were used as inputs to STAR mapper tool (version 2.5.3a)^234^ with the default parameters for alignment against their respective genomes (*S*. *lycopersicum* version 3.2 and *S*. *pennellii* version 2.0) retrieved from the Sol Genomics Network^28^. Approximately 88% and 87% of the *S*. *lycopersicum* and *S*. *pennellii* reads were uniquely mapped to their genomes, respectively.

The libraries were then sorted by query name using picard tools (version 2.18.0), and for each of them, raw read counts were obtained using the python script htseq-count (version 0.7.2)^235^. Differentially expressed (DE) genes were identified using the Bioconductor R package edgeR by comparing the normalized number of reads aligned to each gene in different tissues^236,237^. The Benjamini and Hochberg’s false-discovery rate (FDR) below 0.05 and minimum fold-change of two were the parameters used to consider a gene DE^238^.

### miRNAs in *S. lycopersicum* small RNA-seq libraries

#### Sequencing analysis

Ninety-five raw small RNA data files were retrieved from the NCBI Sequence Read Archive (SRA) with different accession numbers (Supplementary Table S15). The library qualities were evaluated using FastQC software^231^, adapters were removed with Trimmomatic^233^ discarding reads with quality score below 20 and length less than 17 nucleotides and longer than 30 nucleotides. The filtered sequences were mapped and quantified using miRDeep2^239^. miRDeep2 and perl scripts were used on each sequence separately to generate the numbers of the reads for each miRNAs identified.

### Statistical analysis

For the statistical comparisons among the structural and thermodynamic variables of each category (species and/or families), a basic descriptive analysis was performed followed by non-parametric tests (Wilcoxon-Mann-Whitney). Median values were used to perform the statistical comparisons^240^. Statistical significance was set at *p* < 0.1.

## Electronic supplementary material

Supplementary Figures and Tables

Supplementary Table S3

Supplementary Table S4

Supplementary Table S5

Supplementary Table S6

Supplementary Table S7

Supplementary Table S8

Supplementary Table S9

Supplementary Table S12

Supplementary Table S13

Supplementary Table S15

## References

[1] Knapp S (2002). Tobacco to tomatoes: a phylogenetic perspective on fruit diversity in the Solanaceae. J. Exp. Bot..

[2] Peralta IE, Peralta IE, Spooner DM, Spooner DM (2005). Morphological Characterization and Relationships of Wild Tomatoes (Solanum L. sect. Lycopersicon). Monogr. Syst. Bot..

[3] Bai Y, Lindhout P (2007). Domestication and breeding of tomatoes: What have we gained and what can we gain in the future?. Ann. Bot..

[4] Gerszberg A, Hnatuszko-Konka K (2015). Tomato (*Solanum Lycopersicum*) in the service of biotechnology. Plant Cell. Tissue Organ Cult..

[5] Sato, S. & Tabata, S. Tomato Genome Sequence. In *Biotechnology in Agriculture and Forestry* **70**, 175–197 (2016).

[6] Bauchet, G. & Causse, M. Genetic Diversity in Tomato (Solanum lycopersicum) and Its Wild Relatives. *Genet*. *Divers*. *plants* 133–162 (2012).

[7] Kim H-J, Baek K-H, Lee B-W, Choi D, Hur C-G (2011). In silico identification and characterization of microRNAs and their putative target genes in Solanaceae plants. Genome.

[8] Perez-Fons L (2014). A genome-wide metabolomic resource for tomato fruit from Solanum pennellii. Sci. Rep..

[9] Giovannoni JJ (2004). Genetic regulation of fruit development and ripening. Plant Cell.

[10] Campos ML, Carvalho RF, Benedito VA, Peres LE (2010). Small and remarkable: The Micro-Tom model system as a tool to discover novel hormonal functions and interactions. Plant Signal Behav.

[11] Bedinger PA (2011). Interspecific reproductive barriers in the tomato clade: Opportunities to decipher mechanisms of reproductive isolation. Sex. Plant Reprod..

[12] Kimura, S. & Sinha, N. Tomato (Solanum lycopersicum): A model fruit-bearing crop. *Cold Spring Harb*. *Protoc*. **3**, (2008). 10.1101/pdb.emo105 21356708

[13] Kobayashi M (2014). Genome-wide analysis of intraspecific dna polymorphism in ‘micro-tom’, a model cultivar of tomato (solanum lycopersicum). Plant Cell Physiol..

[14] Carvalho, C. R. F. Sutentabilidade E Análise Econômica Da Tomaticultura De Cambuci-Rj Universidade Estadual Do Norte Fluminense Tomaticultura De Cambuci-Rj (2014).

[15] Lukyanenko AN (1991). Disease Resistance in Tomato. Monogr. Theor. Appl. Genet..

[16] Ercolano MR, Sanseverino W, Carli P, Ferriello F, Frusciante L (2012). Genetic and genomic approaches for R-gene mediated disease resistance in tomato: Retrospects and prospects. Plant Cell Rep..

[17] Stevens MA, Rick (1986). C. M. Genetics and Breeding. Science.

[18] Kamenetzky L (2010). Genomic Analysis of Wild Tomato Introgressions Determining Metabolism- and Yield-Associated Traits. Plant Physiol..

[19] Alseekh S (2013). Resolution by recombination: Breaking up Solanum pennellii introgressions. Trends Plant Sci..

[20] Bolger A (2014). The genome of the stress-tolerant wild tomato species Solanum pennellii. Nat. Genet..

[21] Peralta IE, Knapp S, Spooner DM (2005). New Species of Wild Tomatoes (Solanum Section Lycopersicon: Solanaceae) from Northern Peru. Syst. Bot..

[22] Spooner D, Peralta IE, Knapp S (2005). Comparisonof AFLPs with other markers for phylogenetic inference in wild tomatoes. Taxon.

[23] Aflitos S (2014). Exploring genetic variation in the tomato (Solanum section Lycopersicon) clade by whole-genome sequencing. Plant J..

[24] McDaniel T (2016). Novel resistance mechanisms of a wild tomato against the glasshouse whitefly. Agron. Sustain. Dev..

[25] Nosenko Tetyana, Böndel Katharina B., Kumpfmüller Gabriele, Stephan Wolfgang (2016). Adaptation to low temperatures in the wild tomato speciesSolanum chilense. Molecular Ecology.

[26] Mittova, V., Volokita, M. & Guy, M. Antioxidative Systems and Stress Tolerance: Insight from Wild and Cultivated Tomato Species. *Signal*. *Commun*. *Plants* **23**, (2015).

[27] Rick CM, Tanksley SD (1981). Genetic variation in Solanum pennellii: Comparisons with two other sympatric tomato species. Plant Syst. Evol..

[28] Fernandez-Pozo N (2015). The Sol Genomics Network (SGN)-from genotype to phenotype to breeding. Nucleic Acids Res..

[29] Sato S (2012). The tomato genome sequence provides insights into fleshy fruit evolution. Nature.

[30] Hirakawa H (2013). Genome-wide SNP genotyping to infer the effects on gene functions in tomato. DNA Res..

[31] Moazed D (2009). Small RNAs in transcriptional gene silencing and genome defence. Nature.

[32] Axtell MJ (2013). Classification and comparison of small RNAs from plants. Annu. Rev. Plant Biol..

[33] Kang Z (2014). Small RNA regulators in bacteria: Powerful tools for metabolic engineering and synthetic biology. Appl. Microbiol. Biotechnol..

[34] Wen J (2014). Diversity of miRNAs, siRNAs, and piRNAs across 25 Drosophila cell lines. Genome Res..

[35] Vidigal JA, Ventura A (2015). The biological functions of miRNAs: Lessons from *in vivo* studies. Trends Cell Biol..

[36] Fouracre JP, Poethig RS (2016). The role of small RNAs in vegetative shoot development. Curr. Opin. Plant Biol..

[37] Bartel DP (2004). MicroRNAs: Genomics, Biogenesis, Mechanism, and Function Genomics: The miRNA Genes. Cell.

[38] Gomes MS (2009). Preliminary analysis of miRNA pathway in Schistosoma mansoni. Parasitol. Int..

[39] Rao DD, Vorhies JS, Senzer N, Nemunaitis J (2009). siRNA vs. shRNA: similarities and differences. Adv. Drug Deliv. Rev..

[40] Agrawal N (2003). RNA Interference: Biology, Mechanism, and Applications. Microbiol. Mol. Biol. Rev..

[41] Doench JG, Petersen CP, Sharp P (2003). a. siRNAs can function as miRNAs. Genes Dev..

[42] Bentwich I (2005). Prediction and validation of microRNAs and their targets. FEBS Lett..

[43] Wahid F, Shehzad A, Khan T, Kim YY (2010). MicroRNAs: synthesis, mechanism, function, and recent clinical trials. Biochim. Biophys. Acta.

[44] Carthew RW, Sontheimer EJ (2009). Origins and Mechanisms of miRNAs and siRNAs. Cell.

[45] Dhir A, Proudfoot NJ (2013). Feed backwards model for microRNA processing and splicing in plants. Eur. Mol. Biol. Organ. EMBO reports.

[46] Kim VN, Han J, Siomi MC (2009). Biogenesis of small RNAs in animals. Nat. Rev. Mol. Cell Biol..

[47] Jones-Rhoades MW, Bartel DP, Bartel B (2006). MicroRNAs and their regulatory roles in plants. Annu. Rev. Plant Biol..

[48] Kapoor M (2008). Genome-wide identification, organization and phylogenetic analysis of Dicer-like, Argonaute and RNA-dependent RNA Polymerase gene families and their expression analysis during reproductive development and stress in rice. BMC Genomics.

[49] Axtell MJ, Westholm JO, Lai EC (2011). Vive la différence: biogenesis and evolution of microRNAs in plants and animals. Genome Biol..

[50] Waterhouse PM, Hellens RP (2015). Plant biology: Coding in non-coding RNAs. Nature.

[51] Budak H, Akpinar BA (2015). Plant miRNAs: biogenesis, organization and origins. Funct. Integr. Genomics.

[52] Ren G (2012). Regulation of miRNA abundance by RNA binding protein TOUGH in Arabidopsis. Proc. Natl. Acad. Sci..

[53] Wang L (2013). NOT2 Proteins Promote Polymerase II-Dependent Transcription and Interact with Multiple MicroRNA Biogenesis Factors in Arabidopsis. Plant Cell.

[54] Manavella PA (2012). Fast-forward genetics identifies plant CPL phosphatases as regulators of miRNA processing factor HYL1. Cell.

[55] Winter J, Jung S, Keller S, Gregory RI, Diederichs S (2009). Many roads to maturity: microRNA biogenesis pathways and their regulation. Nat. Cell Biol..

[56] Kidner C (2005). a & Martienssen, R. a. The developmental role of microRNA in plants. Curr. Opin. Plant Biol..

[57] Peters L, Meister G (2007). Argonaute proteins: mediators of RNA silencing. Mol. Cell.

[58] Xie M, Zhang S, Yu B (2015). microRNA biogenesis, degradation and activity in plants. Cell. Mol. Life Sci..

[59] Smith MR (2009). Cyclophilin 40 is required for microRNA activity in Arabidopsis. Proc. Natl. Acad. Sci..

[60] Li A, Mao L (2007). Evolution of plant microRNA gene families. Cell Res..

[61] Mallory AC, Vaucheret H (2006). Functions of microRNAs and related small RNAs in plants. Nat. Genet..

[62] Martínez de Alba AE, Elvira-Matelot E, Vaucheret H (2013). Gene silencing in plants: a diversity of pathways. Biochim. Biophys. Acta.

[63] Varkonyi-Gasic E, Wu R, Wood M, Walton EF, Hellens RP (2007). Protocol: a highly sensitive RT-PCR method for detection and quantification of microRNAs. Plant Methods.

[64] Pfaffl MW (2001). A new mathematical model for relative quantification in real-time RT-PCR. Nucleic Acids Res..

[65] Morrison TB, Weis JJ, Wittwer CT (1998). Quantification of lowcopy transcripts by continuous SYBR Green I monitoring during amplification. Biotechniques.

[66] Huang Y (2011). The discovery approaches and detection methods of microRNAs. Mol. Biol. Rep..

[67] Zhang BH, Pan XP, Wang QL, Cobb GP, Anderson TA (2005). Identification and characterization of new plant microRNAs using EST analysis. Cell Res..

[68] Li L, Xu J, Yang D, Tan X, Wang H (2010). Computational approaches for microRNA studies: a review. Mamm. Genome.

[69] Bai M (2012). Genome-wide identification of Dicer-like, Argonaute and RNA-dependent RNA polymerase gene families and their expression analyses in response to viral infection and abiotic stresses in Solanum lycopersicum. Gene.

[70] Mirzaei K, Bahramnejad B, Shamsifard MH, Zamani W (2014). In Silico Identification, Phylogenetic and Bioinformatic Analysis of Argonaute Genes inPlants. Int. J. Genomics.

[71] Wang T (2016). Cloning, identification, and expression analysis of a Dicer-Like gene family from Solanum lycopersicum. Biol. Plant..

[72] Xian Zhiqiang, Yang Yingwu, Huang Wei, Tang Ning, Wang Xinyu, Li Zhengguo (2013). Molecular cloning and characterisation of SlAGO family in tomato. BMC Plant Biology.

[73] Fan S (2015). shan *et al*. Identification of microRNAs in two species of tomato, Solanum lycopersicum and Solanum habrochaites, by deep sequencing. J. Integr. Agric..

[74] Yin Z, Li C, Han X, Shen F (2008). Identification of conserved microRNAs and their target genes in tomato (Lycopersicon esculentum). Gene.

[75] Pradhan B, Naqvi AR, Saraf S, Mukherjee SK, Dey N (2015). Prediction and characterization of Tomato leaf curl New Delhi virus (ToLCNDV) responsive novel microRNAs in Solanum lycopersicum. Virus Res..

[76] Meng J, Liu D, Sun C, Luan Y (2014). Prediction of plant pre-microRNAs and their microRNAs in genome-scale sequences using structure-sequence features and support vector machine. BMC Bioinformatics.

[77] Moxon S (2008). Deep sequencing of tomato short RNAs identifies microRNAs targeting genes involved in fruit ripening. Genome Res..

[78] Zhao G, Yu H, Liu M, Lu Y, Ouyang B (2017). Identification of salt-stress responsive microRNAs from Solanum lycopersicum and Solanum pimpinellifolium. Plant Growth Regul..

[79] Tandon G (2017). Computational deciphering of biotic stress associated genes in tomato (Solanum lycopersicum). Genomics Data.

[80] Din M, Barozai MYK (2014). Profiling microRNAs and their targets in an important fleshy fruit: Tomato (Solanum lycopersicum). Gene.

[81] Gu M (2014). Identification of microRNAs in six solanaceous plants and their potential link with phosphate and mycorrhizal signaling. J. Integr. Plant Biol..

[82] Jin W, Wu F (2015). Characterization of miRNAs associated with Botrytis cinerea infection of tomato leaves. BMC Plant Biol..

[83] Liu M (2018). Identification of drought-responsive microRNAs in tomato using high-throughput sequencing. Funct. Integr. Genomics.

[84] Omidvar V, Mohorianu I, Dalmay T, Fellner M (2015). Identification of miRNAs with potential roles in regulation of anther development and male-sterility in 7B-1 male-sterile tomato mutant. BMC Genomics.

[85] Sarkar D (2017). Integrated miRNA and mRNA expression profiling reveals the response regulators of a susceptible tomato cultivar to early blight disease. DNA Res..

[86] Pan C (2017). Identification and expression profiling of microRNAs involved in the stigma exsertion under high-temperature stress in tomato. BMC Genomics.

[87] Xu D, Guo S, Dongqian X (2013). Identification of Conserved miRNAs in Solanum Lycopersicum Response to Long-term RPM-treatment. J. Life Sci. Technol..

[88] Srivastava PK, Moturu TR, Pandey P, Baldwin IT, Pandey SP (2014). A comparison of performance of plant miRNA target prediction tools and the characterization of features for genome-wide target prediction. BMC Genomics.

[89] Zhou X, Wang G, Sutoh K, Zhu J-K, Zhang W (2008). Identification of cold-inducible microRNAs in plants by transcriptome analysis. Biochim. Biophys. Acta.

[90] Ambros V (2003). A uniform system for microRNA annotation A uniform system for microRNA annotation. RNA - A Publ. RNA Soc..

[91] Bologna NG, Voinnet O (2014). The diversity, biogenesis, and activities of endogenous silencing small RNAs in Arabidopsis. Annu. Rev. Plant Biol..

[92] Liu T (2016). Global investigation of the co-evolution of MIRNA genes and microRNA targets during soybean domestication. Plant J..

[93] Dai Q (2017). Comparative transcriptome analysis of the different tissues between the cultivated and wild tomato. PLoS One.

[94] Mallory A, Vaucheret H (2010). Form, function, and regulation of ARGONAUTE proteins. Plant Cell.

[95] de Sousa Cardoso TC (2015). Genome-wide identification and *in silico* characterisation of microRNAs, their targets and processing pathway genes in *Phaseolus vulgaris* L. Plant Biol..

[96] Chen X (2008). MicroRNA Metabolism in Plants. Curr. Top. Microbiol. Immunol..

[97] Till S (2007). A conserved motif in Argonaute-interacting proteins mediates functional interactions through the Argonaute PIWI domain. Nat. Struct. Mol. Biol..

[98] Baumberger N, Baulcombe DC (2005). Arabidopsis ARGONAUTE1 is an RNA Slicer that selectively recruits microRNAs and short interfering RNAs. Proc. Natl. Acad. Sci. USA.

[99] Zhu H (2011). Arabidopsis argonaute10 specifically sequesters miR166/165 to regulate shoot apical meristem development. Cell.

[100] Contreras-Cubas C, Palomar M, Arteaga-Vázquez M, Reyes JL, Covarrubias AA (2012). Non-coding RNAs in the plant response to abiotic stress. Planta.

[101] Gasciolli V, Mallory AC, Bartel DP, Vaucheret H (2005). Partially redundant functions of Arabidopsis DICER-like enzymes and a role for DCL4 in producing trans-acting siRNAs. Curr. Biol..

[102] Xie Z (2004). Genetic and functional diversification of small RNA pathways in plants. PLoS Biol..

[103] Brodersen P, Voinnet O (2006). The diversity of RNA silencing pathways in plants. Trends Genet..

[104] Song L, Han M, Lesicka J, Fedoroff N (2007). Arabidopsis primary microRNA processing proteins HYL1 and DCL1 define a nuclear body distinct from the Cajal body. Proc. Natl. Acad. Sci..

[105] Liu Qingpo, Feng Ying, Zhu Zhujun (2009). Dicer-like (DCL) proteins in plants. Functional & Integrative Genomics.

[106] Liu B (2005). Loss of Function of OsDCL1 Affects MicroRNA Accumulation and Causes Developmental. Plant Physiol..

[107] Finnegan EJ, Margis R, Waterhouse PM (2003). Posttranscriptional gene silencing is not compromised in the Arabidopsis CARPEL FACTORY (DICER-LIKE1) mutant, a homolog of Dicer-1 from Drosophila. Curr. Biol..

[108] Margis R (2006). The evolution and diversification of Dicers in plants. FEBS Lett..

[109] Allen E, Xie Z, Gustafson AM, Carrington J (2005). C. microRNA-directed phasing during trans-acting siRNA biogenesis in plants. Cell.

[110] Reinhart BJ, Weinstein EG, Rhoades MW, Bartel B, Bartel DP (2002). MicroRNAs in plants. Genes Dev..

[111] Rogers K, Chen X (2013). Biogenesis, turnover, and mode of action of plant microRNAs. Plant Cell.

[112] Ji X (2008). The Mechanism of RNase III Action: How Dicer Dices. Curr. Top. Microbiol. Immunol..

[113] Höck J, Meister G (2008). The Argonaute protein family. Genome Biol..

[114] Stevens, P. F. Angiosperm Phylogeny Website. *Version 12*, *July 2012* Available at, http://www.mobot.org/MOBOT/research/APweb/ (2001).

[115] Mallory AC, Vaucheret H (2006). Functions of microRNAs and related small RNAs in plants. Nat. Genet..

[116] Aukerman MJ, Sakai H (2003). Regulation of Flowering Time and Floral Organ Identity by a MicroRNA and Its APETALA2 -Like Target Genes. Plant Cell.

[117] Wang JW (2014). Regulation of flowering time by the miR156-mediated age pathway. J. Exp. Bot..

[118] Yamaguchi A, Abe M (2012). Regulation of reproductive development by non-coding RNA in Arabidopsis: To flower or not to flower. J. Plant Res..

[119] Araújo DS (2016). Exossomos: estruturas promissoras para o diagnóstico e tratamento de doenças e regulação dos processos de interação parasito-hospedeiro. Refacer.

[120] Sikorska, N., Zuber, H., Gobert, A., Lange, H. & Gagliardi, D. RNA degradation by the plant RNA exosome involves both phosphorolytic and hydrolytic activities. *Nat*. *Commun*. **8**, (2017). 10.1038/s41467-017-02066-2 PMC5735172 29255150

[121] Ji L (2011). ARGONAUTE10 and ARGONAUTE1 regulate the termination of floral stem cells through two microRNAs in Arabidopsis. PLoS Genet..

[122] Liu Y (2014). Genome-wide comparison of microRNAs and their targeted transcripts among leaf, flower and fruit of sweet orange. BMC Genomics.

[123] Vaucheret H, Vazquez F, Crété P, Bartel DP (2004). The action of ARGONAUTE1 in the miRNA pathway and its regulation by the miRNA pathway are crucial for plant development. Genes Dev..

[124] Kozomara A, Griffiths-Jones S (2014). MiRBase: Annotating high confidence microRNAs using deep sequencing data. Nucleic Acids Res..

[125] Luan Y, Wang W, Liu P (2014). Identification and functional analysis of novel and conserved microRNAs in tomato. Mol. Biol. Rep..

[126] Zhang J, Zeng R, Chen J, Liu X, Liao Q (2008). Identification of conserved microRNAs and their targets from Solanum lycopersicum Mill. Gene.

[127] Sunkar R, Jagadeeswaran G (2008). In silico identification of conserved microRNAs in large number of diverse plant species. BMC Plant Biol..

[128] Itaya A (2008). Small RNAs in tomato fruit and leaf development. Biochim. Biophys. Acta.

[129] Zhang B, Pan X, Cannon CH, Cobb GP, Anderson TA (2006). Conservation and divergence of plant microRNA genes. Plant J..

[130] Lang Q (2011). Microarray-based identification of tomato microRNAs and time course analysis of their response to Cucumber mosaic virus infection. J. Zhejiang Univ. Sci. B.

[131] Zuo J (2012). Sculpting the maturation, softening and ethylene pathway: the influences of microRNAs on tomato fruits. BMC Genomics.

[132] Xu D, Guo S, Liu M (2013). Identification of miRNAs involved in long-term simulated microgravity response in Solanum lycopersicum. Plant Physiol. Biochem..

[133] Valiollahi E, Farsi M, Fevereiro P, Kakhki AM (2014). Bioinformatic characterization and expression analysis of miRNAs in Solanum lycopersicum. Plant Omics.

[134] Prakash P, Ghosliya D, Gupta V (2014). Identification of conserved and novel microRNAs in Catharanthus roseus by deep sequencing and computational prediction of their potential targets. Gene.

[135] Sun Jie, Zhou Meng, Mao Zhitao, Li Chuanxing (2012). Characterization and Evolution of microRNA Genes Derived from Repetitive Elements and Duplication Events in Plants. PLoS ONE.

[136] Liu J (2012). Genome-wide analysis uncovers regulation of long intergenic noncoding RNAs in Arabidopsis. Plant Cell.

[137] Zhou M (2011). Genome-wide analysis of clustering patterns and flanking characteristics for plant microRNA genes. FEBS J..

[138] Zhang BH, Pan XP, Cox SB, Cobb GP, Anderson T (2006). a. Evidence that miRNAs are different from other RNAs. Cell. Mol. Life Sci..

[139] Zhang B, Pan X, Stellwag EJ (2008). Identification of soybean microRNAs and their targets. Planta.

[140] Nageshbabu R, Jyothi MN, Sharadamma N, Rai DV, Devaraj VR (2012). Computational Identification of conserved miRNAs and their potential targets in French bean (Phaseolus vulgaris). Res. J. Pharm., Biol. Chem. Sci..

[141] Thakur, V. *et al*. Characterization of statistical features for plant microRNA prediction. *BMC Genomics* **12**, (2011). 10.1186/1471-2164-12-108 PMC3053258 21324149

[142] Zhan Shuhua, Lukens Lewis (2010). Identification of Novel miRNAs and miRNA Dependent Developmental Shifts of Gene Expression in Arabidopsis thaliana. PLoS ONE.

[143] Zhou L (2010). Genome-wide identification and analysis of drought-responsive microRNAs in Oryza sativa. J. Exp. Bot..

[144] Zhi-hao W, Hui-hui J, Qing-shan C, Rong-sheng Z (2015). Evolution Analysis About Soybean MIR166 Family. J. Northeast Agric. Univ. (English Ed..

[145] Jones-Rhoades MW, Bartel DP (2004). Computational identification of plant MicroRNAs and their targets, including a stress-induced miRNA. Mol. Cell.

[146] Bonnet E, Wuyts J, Rouzé P, Van de Peer Y (2004). Detection of 91 potential conserved plant microRNAs in Arabidopsis thaliana and Oryza sativa identifies important target genes. Proc. Natl. Acad. Sci. USA.

[147] Barozai Muhammad Younas Khan, Baloch Iftikhar Ahmed, Din Muhammad (2011). Identification of MicroRNAs and their targets in Helianthus. Molecular Biology Reports.

[148] Montes RaC (2014). Sample sequencing of vascular plants demonstrates widespread conservation and divergence of microRNAs. Nat. Commun..

[149] Zhang L (2009). A genome-wide characterization of microRNA genes in maize. PLoS Genet..

[150] Jagadeeswaran G (2009). Cloning and characterization of small RNAs from Medicago truncatula reveals four novel legume-specific microRNA families. New Phytol..

[151] Lakhotia N (2014). Identification and characterization of miRNAome in root, stem, leaf and tuber developmental stages of potato (Solanum tuberosum L.) by high-throughput sequencing. BMC Plant Biol..

[152] Khaldun ABM, Huang W, Liao S, Lv H, Wang Y (2015). Identification of MicroRNAs and target genes in the fruit and shoot tip of Lycium chinense: A traditional chinese medicinal plant. PLoS One.

[153] Lu S, Sun YH, Chiang VL (2008). Stress-responsive microRNAs in Populus. Plant J..

[154] Xue L-J, Zhang J-J, Xue H-W (2009). Characterization and expression profiles of miRNAs in rice seeds. Nucleic Acids Res..

[155] Zhang Runxuan, Marshall David, Bryan Glenn J., Hornyik Csaba (2013). Identification and Characterization of miRNA Transcriptome in Potato by High-Throughput Sequencing. PLoS ONE.

[156] Wang L, Liu H, Li D, Chen H (2011). Identification and characterization of maize microRNAs involved in the very early stage of seed germination. BMC Genomics.

[157] Zheng Y, Li Y-F, Sunkar R, Zhang W (2012). SeqTar: an effective method for identifying microRNA guided cleavage sites from degradome of polyadenylated transcripts in plants. Nucleic Acids Res..

[158] Rosas-Cárdenas Flor de Fátima, Caballero-Pérez Juan, Gutiérrez-Ramos Ximena, Marsch-Martínez Nayelli, Cruz-Hernández Andrés, de Folter Stefan (2014). miRNA expression during prickly pear cactus fruit development. Planta.

[159] Rajagopalan R, Vaucheret H, Trejo J, Bartel DP (2006). A diverse and evolutionarily fluid set of microRNAs in Arabidopsis thaliana. Genes Dev..

[160] Zhou R (2016). Identification of miRNAs and their targets in wild tomato at moderately and acutely elevated temperatures by high-throughput sequencing and degradome analysis. Sci. Rep..

[161] Letunic I, Bork P (2016). Interactive tree of life (iTOL)v3: an online tool for the display and annotation of phylogenetic and other trees. Nucleic Acids Res..

[162] Du Qian, Wang Huanzhong (2015). The role of HD-ZIP III transcription factors and miR165/166 in vascular development and secondary cell wall formation. Plant Signaling & Behavior.

[163] Ko JH, Prassinos C, Han KH (2006). Developmental and seasonal expression of PtaHB1, a Populus gene encoding a class III HD-Zip protein, is closely associated with secondary growth and inversely correlated with the level of microRNA (miR166). New Phytol..

[164] Barik S (2014). Phylogenetic analysis reveals conservation and diversification of micro RNA166 genes among diverse plant species. Genomics.

[165] Turchi L, Baima S, Morelli G, Ruberti I (2015). Interplay of HD-Zip II and III transcription factors in auxin-regulated plant development. J. Exp. Bot..

[166] Itoh J-I, Hibara K-I, Sato Y, Nagato Y (2008). Developmental role and auxin responsiveness of Class III homeodomain leucine zipper gene family members in rice. Plant Physiol..

[167] Sakaguchi J, Watanabe Y (2012). miR165/166 and the development of land plants. Dev. Growth Differ..

[168] Sunkar R, Zhou X, Zheng Y, Zhang W, Zhu J-K (2008). Identification of novel and candidate miRNAs in rice by high throughput sequencing. BMC Plant Biol..

[169] Sun X (2013). PMTED: A plant microRNA target expression database. BMC Bioinformatics.

[170] Mangrauthia Satendra K., Bhogireddy Sailaja, Agarwal Surekha, Prasanth Vishnu V., Voleti S. R., Neelamraju Sarla, Subrahmanyam Desiraju (2017). Genome-wide changes in microRNA expression during short and prolonged heat stress and recovery in contrasting rice cultivars. Journal of Experimental Botany.

[171] Song C, Fang J, Li X, Liu H, Thomas Chao C (2009). Identification and characterization of 27 conserved microRNAs in citrus. Planta.

[172] Fileccia Veronica, Bertolini Edoardo, Ruisi Paolo, Giambalvo Dario, Frenda Alfonso Salvatore, Cannarozzi Gina, Tadele Zerihun, Crosatti Cristina, Martinelli Federico (2017). Identification and characterization of durum wheat microRNAs in leaf and root tissues. Functional & Integrative Genomics.

[173] Li S (2013). MicroRNAs inhibit the translation of target mRNAs on the endoplasmic reticulum in Arabidopsis. Cell.

[174] Li H (2011). Characterization of the stress associated microRNAs in Glycine max by deep sequencing. BMC Plant Biol..

[175] Guo R (2016). Identification of miRNAs Affecting the Establishment of Brassica Alboglabra Seedling. Front. Plant Sci..

[176] Ru P, Xu L, Ma H, Huang H (2006). Plant fertility defects induced by the enhanced expression of microRNA167. Cell Res..

[177] Li Y (2010). Identification of MicroRNAs Involved in Pathogen-Associated Molecular Pattern-Triggered Plant Innate Immunity. Plant Physiol..

[178] Kinoshita N (2012). IAA-Ala Resistant3, an Evolutionarily Conserved Target of miR167, Mediates Arabidopsis Root Architecture Changes during High Osmotic Stress. Plant Cell.

[179] Liu W (2014). Analysis of miRNAs and their targets during adventitious shoot organogenesis of Acacia crassicarpa. PLoS One.

[180] Baldrich P (2015). MicroRNA-mediated regulation of gene expression in the response of rice plants to fungal elicitors. RNA Biol..

[181] Pashkovskiy PP, Kartashov AV, Zlobin IE, Pogosyan SI, Kuznetsov VV (2016). Blue light alters miR167 expression and microRNA-targeted auxin response factor genes in Arabidopsis thaliana plants. Plant Physiol. Biochem..

[182] Xie F, Frazier TP, Zhang B (2011). Identification, characterization and expression analysis of MicroRNAs and their targets in the potato (Solanum tuberosum). Gene.

[183] Fang Y-N (2016). High-throughput sequencing and degradome analysis reveal altered expression of miRNAs and their targets in a male-sterile cybrid pummelo (Citrus grandis). BMC Genomics.

[184] Barciszewska-Pacak M (2015). Arabidopsis microRNA expression regulation in a wide range of abiotic stress responses. Front. Plant Sci..

[185] Zhao M (2012). Cloning and characterization of maize miRNAs involved in responses to nitrogen deficiency. PLoS One.

[186] Li ZF, Zhang YC, Chen YQ (2015). MiRNAs and lncRNAs in reproductive development. Plant Sci..

[187] Chaves SS (2015). New Insights on Coffea miRNAs: Features and Evolutionary Conservation. Appl. Biochem. Biotechnol..

[188] Kwak PB, Wang QQ, Chen XS, Qiu CX, Yang ZM (2009). Enrichment of a set of microRNAs during the cotton fiber development. BMC Genomics.

[189] Chen ZH (2011). Regulation of auxin response by miR393-targeted transport inhibitor response protein 1 is involved in normal development in Arabidopsis. Plant Mol. Biol..

[190] Robert-Seilaniantz A (2011). The microRNA miR393 re-directs secondary metabolite biosynthesis away from camalexin and towards glucosinolates. Plant J..

[191] Windels D, Vazquez F (2011). miR393: Integrator of environmental cues in auxin signaling?. Plant Signal. Behav..

[192] Si-Ammour A (2011). miR393 and Secondary siRNAs Regulate Expression of the TIR1/AFB2 Auxin Receptor Clade and Auxin-Related Development of Arabidopsis Leaves. Plant Physiol..

[193] Feng J, Liu S, Wang M, Lang Q, Jin C (2014). Identification of microRNAs and their targets in tomato infected with Cucumber mosaic virus based on deep sequencing. Planta.

[194] Gao P (2011). Osa-MIR393: A salinity- and alkaline stress-related microRNA gene. Mol. Biol. Rep..

[195] Bai B (2017). MiR393-Mediated Auxin Signaling Regulation is Involved in Root Elongation Inhibition in Response to Toxic Aluminum Stress in Barley. Plant Cell Physiol..

[196] Lang Q (2011). Tobacco microRNAs prediction and their expression infected with Cucumber mosaic virus and Potato virus X. Mol. Biol. Rep..

[197] Ouyang Shouqiang, Park Gyungsoon, Atamian Hagop S., Han Cliff S., Stajich Jason E., Kaloshian Isgouhi, Borkovich Katherine A. (2014). MicroRNAs Suppress NB Domain Genes in Tomato That Confer Resistance to Fusarium oxysporum. PLoS Pathogens.

[198] Shen J, Xie K, Xiong L (2010). Global expression profiling of rice microRNAs by one-tube stem-loop reverse transcription quantitative PCR revealed important roles of microRNAs in abiotic stress responses. Mol. Genet. Genomics.

[199] Kohli Deshika, Joshi Gopal, Deokar Amit Atmaram, Bhardwaj Ankur R., Agarwal Manu, Katiyar-Agarwal Surekha, Srinivasan Ramamurthy, Jain Pradeep Kumar (2014). Identification and Characterization of Wilt and Salt Stress-Responsive MicroRNAs in Chickpea through High-Throughput Sequencing. PLoS ONE.

[200] Nuruzzaman M, Sharoni AM, Kikuchi S (2013). Roles of NAC transcription factors in the regulation of biotic and abiotic stress responses in plants. Front. Microbiol..

[201] Lin SI (2010). Complex regulation of two target genes encoding SPX-MFS proteins by rice miR827 in response to phosphate starvation. Plant Cell Physiol..

[202] Kuo H-F, Chiou T-J (2011). The role of microRNAs in phosphorus deficiency signaling. Plant Physiol..

[203] Pant BD (2009). Identification of nutrient-responsive Arabidopsis and rapeseed microRNAs by comprehensive real-time polymerase chain reaction profiling and small RNA sequencing. Plant Physiol..

[204] Xu Zhenhua, Zhong Sihui, Li Xinhai, Li Wenxue, Rothstein Steven J., Zhang Shihuang, Bi Yongmei, Xie Chuanxiao (2011). Genome-Wide Identification of MicroRNAs in Response to Low Nitrate Availability in Maize Leaves and Roots. PLoS ONE.

[205] Lundmark M, Kørner CJ, Nielsen TH (2010). Global analysis of microRNA in Arabidopsis in response to phosphate starvation as studied by locked nucleic acid-based microarrays. Physiol. Plant..

[206] Guan X (2014). miR828 and miR858 regulate homoeologous MYB2 gene functions in Arabidopsis trichome and cotton fibre development. Nat. Commun..

[207] Lin JS, Lin CC, Lin HH, Chen YC, Jeng ST (2012). MicroR828 regulates lignin and h2O2 accumulation in sweet potato on wounding. New Phytol..

[208] Jeyaraj A, Chandran V, Gajjeraman P (2014). Differential expression of microRNAs in dormant bud of tea [Camellia sinensis (L.) O. Kuntze]. Plant Cell Rep..

[209] Wang C (2011). Characterization of microRNAs identified in a table grapevine cultivar with validation of computationally predicted grapevine miRNAs by miR-RACE. PLoS One.

[210] Ambawat S, Sharma P, Yadav NR, Yadav RC (2013). MYB transcription factor genes as regulators for plant responses: An overview. Physiol. Mol. Biol. Plants.

[211] Sood P, Krek A, Zavolan M, Macino G, Rajewsky N (2006). Cell-type-specific signatures of microRNAs on target mRNA expression. Proc. Natl. Acad. Sci..

[212] Budak H, Khan Z, Kantar M (2014). History and current status of wheat miRNAs using next-generation sequencing and their roles in development and stress. Brief. Funct. Genomics.

[213] Altschul SF (1997). Gapped BLAST and PSI-BLAST:a new generation of protein database search programs. Nucleic Acids Res..

[214] Altschul SF, Gish W, Miller W, Myers EW, Lipman DJ (1990). Basic Local Alignment Search Tool. J. Mol. Biol..

[215] Goodstein DM (2012). Phytozome: A comparative platform for green plant genomics. Nucleic Acids Res..

[216] de Souza Gomes M, Muniyappa MK, Carvalho SG, Guerra-Sá R, Spillane C (2011). Genome-wide identification of novel microRNAs and their target genes in the human parasite Schistosoma mansoni. Genomics.

[217] Li Weizhong, Cowley Andrew, Uludag Mahmut, Gur Tamer, McWilliam Hamish, Squizzato Silvano, Park Young Mi, Buso Nicola, Lopez Rodrigo (2015). The EMBL-EBI bioinformatics web and programmatic tools framework. Nucleic Acids Research.

[218] Griffiths-Jones S, Grocock RJ, van Dongen S, Bateman A, Enright AJ (2006). miRBase: microRNA sequences, targets and gene nomenclature. Nucleic Acids Res..

[219] Gardner PP (2009). Rfam: updates to the RNA families database. Nucleic Acids Res..

[220] Larkin MA (2007). Clustal W and Clustal X version 2.0. Bioinformatics.

[221] Takane, K. *et al*. Computational prediction and experimental validation of evolutionarily conserved microRNA target genes in bilaterian animals. *BMC Genomics* 1–13 (2010). 10.1186/1471-2164-11-101 PMC2833159 20144220

[222] Crooks GE, Hon G, Chandonia J, Brenner SE (2004). WebLogo: A Sequence Logo Generator. Genome Res..

[223] Finn Robert D., Coggill Penelope, Eberhardt Ruth Y., Eddy Sean R., Mistry Jaina, Mitchell Alex L., Potter Simon C., Punta Marco, Qureshi Matloob, Sangrador-Vegas Amaia, Salazar Gustavo A., Tate John, Bateman Alex (2015). The Pfam protein families database: towards a more sustainable future. Nucleic Acids Research.

[224] Marchler-Bauer, A. *et al*. CDD: NCBI’s conserved domain database. *Nucleic Acids Res*. **43**, (2015). 10.1093/nar/gku1221 PMC4383992 25414356

[225] Tamura K (2011). MEGA5: molecular evolutionary genetics analysis using maximum likelihood, evolutionary distance, and maximum parsimony methods. Mol. Biol. Evol..

[226] Saitou N, Nei M (1987). The Neighbor-joining Method: A New Method for Reconstructing Phylogenetic Trees’. Mol. Biol. Evol..

[227] Jones D, Taylor W, Thornton J (1992). The Rapid Generation of Mutation Data Matrices From Protein Sequences. Comput. Appl. Biosci..

[228] Dai X, Zhao P (2011). X. psRNATarget: a plant small RNA target analysis server. Nucleic Acids Res..

[229] Dai, X., Z Zhuang & Zhao, P. X. psRNATarget: A Plant Small RNA Target Analysis Server (2017 update). *Unpublished* (2017). 10.1093/nar/gky316 PMC6030838 29718424

[230] Chen X (2004). A MicroRNA as a Translational Repressor of APETALA2 in Arabidopsis Flower Development. Science (80-.)..

[231] Kroll KW (2014). Quality control for RNA-Seq (QuaCRS): An integrated quality control pipeline. Cancer Inform..

[232] Davis MPA, van Dongen S, Abreu-Goodger C, Bartonicek N, Enright AJ (2013). Kraken: A set of tools for quality control and analysis of high-throughput sequence data. Methods.

[233] Bolger AM, Lohse M, Usadel B (2014). Trimmomatic: A flexible trimmer for Illumina sequence data. Bioinformatics.

[234] Dobin A (2013). STAR: Ultrafast universal RNA-seq aligner. Bioinformatics.

[235] Anders S, Pyl PT, Huber W (2015). HTSeq-A Python framework to work with high-throughput sequencing data. Bioinformatics.

[236] Robinson MD, McCarthy DJ, Smyth G (2009). K. edgeR: A Bioconductor package for differential expression analysis of digital gene expression data. Bioinformatics.

[237] Huber W (2015). Orchestrating high-throughput genomic analysis with Bioconductor. Curr Vasc Pharmacol.

[238] Benjamini Y, Hochberg Y (1995). Controlling the False Discovery Rate: A Practical and Powerful Approach to MultipleTesting. J. R. Stat. Soc..

[239] Friedländer MR, MacKowiak SD, Li N, Chen W, Rajewsky N (2012). MiRDeep2 accurately identifies known and hundreds of novel microRNA genes in seven animal clades. Nucleic Acids Res..

[240] Moisés, LE WMW T. Na *Enciclopédia de Bioestatística* (ed. Sons, JW) 1-3, 10.1002/0470011815.b2a15178 (2005).

