# Supplementary material for: New insights into tomato microRNAs
Source: Sci Rep. 2018 Oct 30;8:16069. doi: 10.1038/s41598-018-34202-3 (PMC6207730; doi:10.1038/s41598-018-34202-3)
Supplement: Supplementary file 1 — Supplementary Figures and Tables [file 41598_2018_34202_MOESM1_ESM.pdf]

New insights into tomato microRNAs

Thaís Cunha de Sousa Cardoso[1], Tamires Caixeta Alves[1], Carolina Milagres Caneschi, Douglas dos Reis Gomes Santana[1], Christiane Noronha Fernandes-Brum[2], Gabriel Lasmar Dos Reis[3], Matheus Martins Daude[4], Thales Henrique Cherubino Ribeiro[2], Miguel Maurício Díaz Gómez[1], André Almeida Lima[2], Luiz Antônio Augusto Gomes[3], Marcos de Souza Gomes[1], Peterson Elizandro Gandolfi[1], Laurence Rodrigues do Amaral[1], Antonio Chalfun-Júnior[2], Wilson Roberto Maluf[3], Matheus de Souza Gomes[1]*

[1]Laboratory of Bioinformatics and Molecular Analysis, Federal University of Uberlandia (UFU), Campus Patos de Minas, Patos de Minas, 38700-128, Brazil.
[2]Laboratory of Plant Molecular Physiology, Federal University of Lavras (UFLA), Lavras, 3037 - 37200-000, Brazil.
[3]Department of Agriculture, Federal University of Lavras (UFLA), Lavras, 37 - 37200-000, Brazil.
[4]Laboratory of Molecular Analysis, Federal University of Tocantins (UFT), Gurupi, 77402-970, Brazil.

* matheusgomes@ufu.br

Supplementary Figures

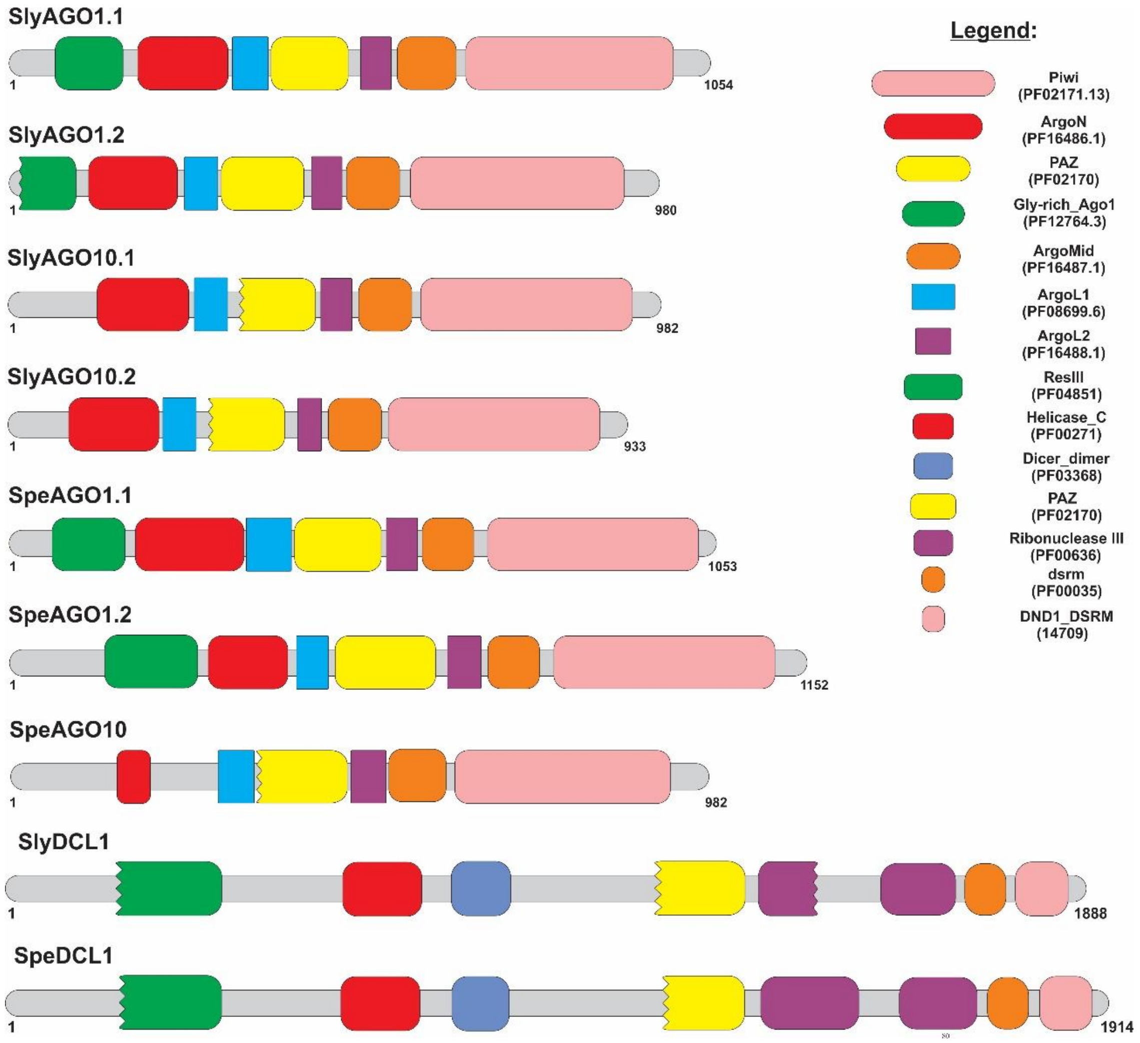

Supplementary Figure S1. Distribution of conserved domains in AGO and DCL putative proteins of *S. lycopersicum* and *S. pennellii*. The SlyAGO1.1/1.2 e SpeAGO1.1/1.2 proteins displayed the Gly-rich_Ago1, ArgoN, ArgoL1, PAZ, ArgoL2, ArgoMid and Piwi in positions 70-174, 185-328, 338-388, 394-520, 531-576, 587-661, 681-1000 / 18-100, 111-254, 264-314, 320-446, 457-502, 513-587, 607-926 e 69-173, 193-327, 337-387, 393-518, 529-575, 586-660, 680-999 / 160-272, 292-426, 436-486, 492-617, 685-759, 685-759, 779-1098, respectively. The same for SlyAGO10.1/10.2 and SpeAGO10 proteins which displayed the domains ArgoN, ArgoL1, PAZ, ArgoL2, ArgoMid and Piwi. The location these domains in SlyAGO10.1/10.2 and SpeAGO10 were at positions 127-270, 280-330, 342-462, 473-518, 529-606, 621-940 / 82-224, 234-284, 302-416, 429-472, 483-561, 575-891. The SlyDCL1 and SpeDCL1 proteins, only DCL involved in the processing of miRNAs, displayed the conserved domains ResIII (also known as DEAD-like), Helicase C, Dicer Dimer (also known as DUF283), PAZ, Ribonuclease IIIa (RNase IIIa), Ribonuclease IIIb (RNase IIIb), dsrm and DND1 DSRM, in 259-420, 656-773, 848-936, 1214-1349, 1386-1468, 1586-1699, 1726-1784, 1811-1883; e 256-420, 656-773, 848-936, 1214-1349, 1386-1534, 1612-1725, 1752-1810, 1837-1909 positions, respectively.

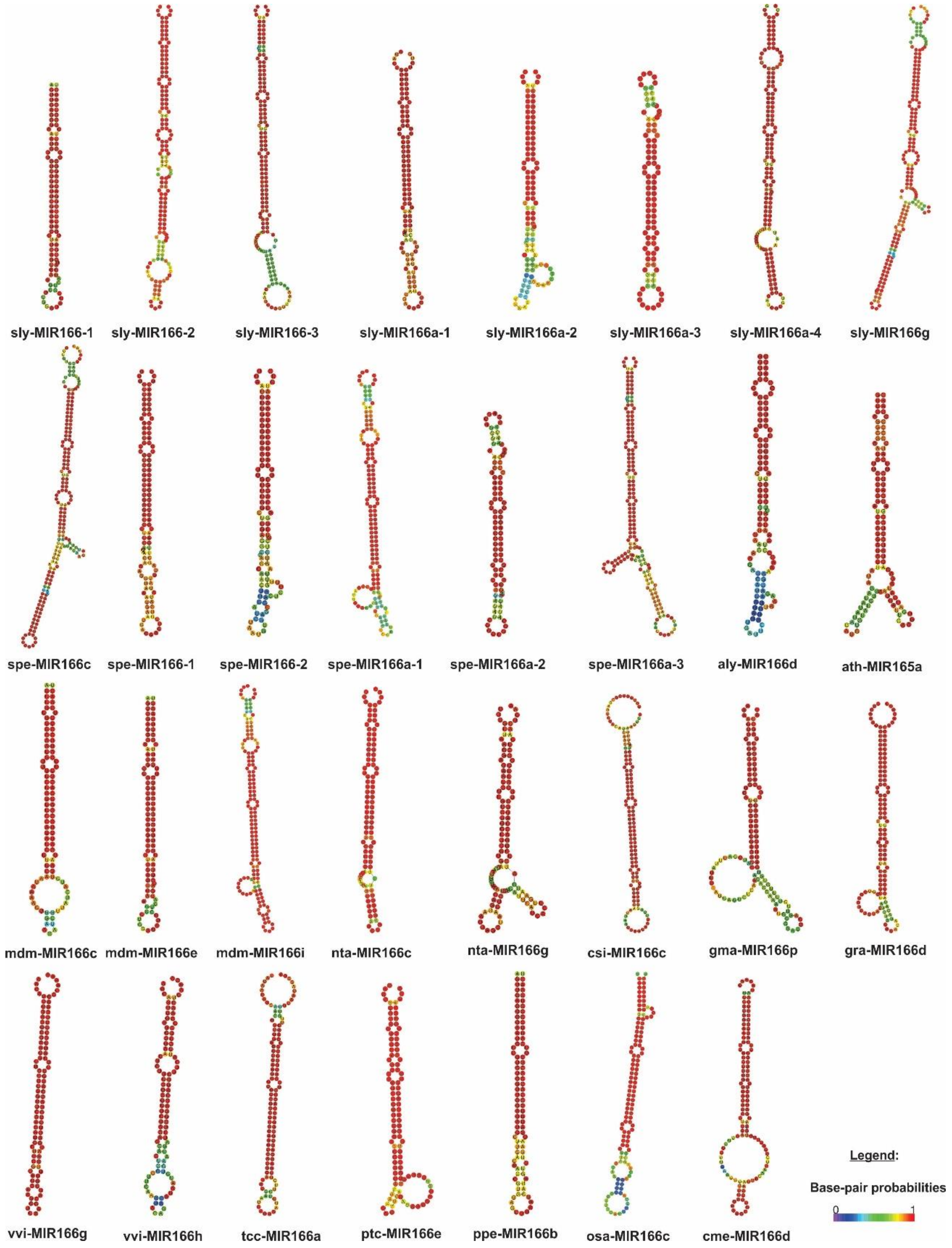

Supplementary Figure S2. Secondary structures of MIR165/166 family pre-miRNAs from *S. lycopersicum*, *S. pennellii* and their orthologs were draw by RNAfold.

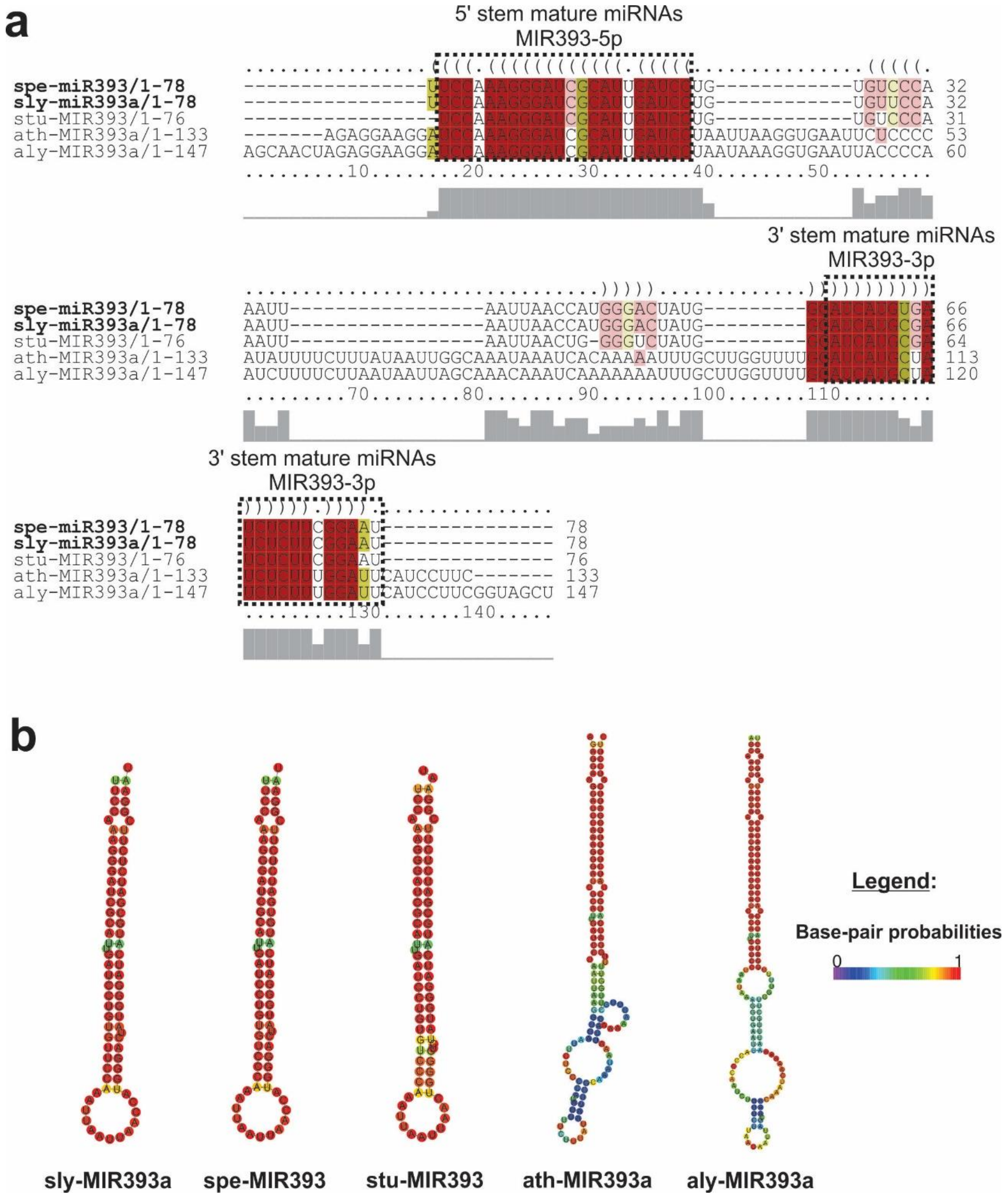

Supplementary Figure S3. (a) The high confidence of RNA alignments between MIR393 family of *S. lycopersicum* and *S. pennellii* to their orthologs were performed using RNAalifold. Mature miRNAs are shown as boxes. Brackets and colors identify matching residues in 5' and 3' stems of hairpin structures. Level of nucleotide identity is indicated below the alignment. **(b)** Secondary structures of MIR393 family from *S. lycopersicum, S. pennellii* and their orthologs were draw by RNAfold. Sly - *Solanum lycopersicum,* Spe - *Solanum pennellii,* Stu – *Solanum tuberosum,* Ath - *Arabidopsis thaliana,* Aly - *Arabidopsis lyrata.*

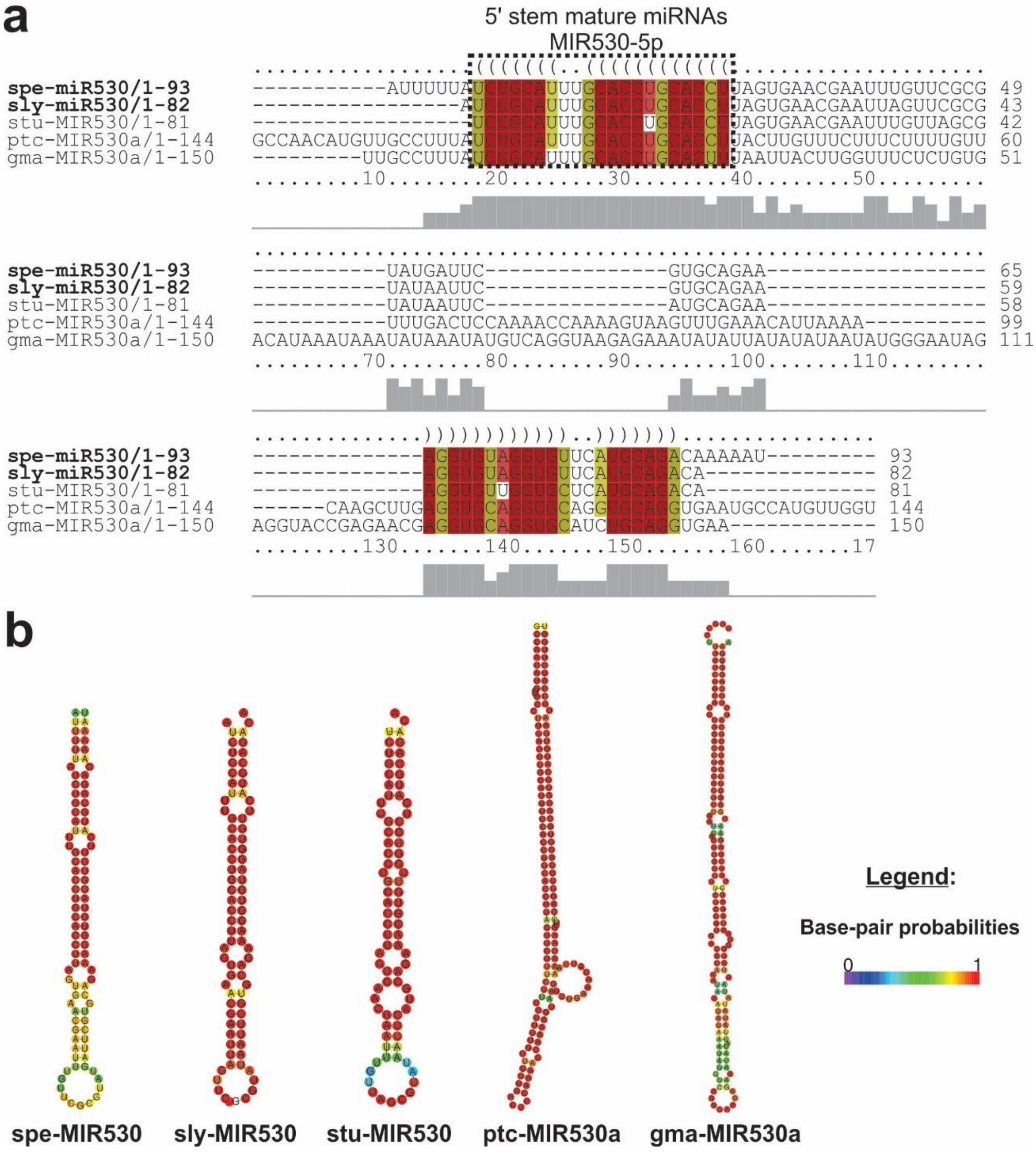

Supplementary Figure S4. (a) The high confidence of RNA alignments between MIR530 family of *S. lycopersicum* and *S. pennellii* to their orthologs were performed using RNAalifold. Mature miRNAs are shown as boxes. Brackets and colors identify matching residues in 5' stems of hairpin structures. Level of nucleotide identity is indicated below the alignment. **(b)** Secondary structures of MIR530 family from *S. lycopersicum, S. pennellii* and their orthologs were draw by RNAfold. Sly - *Solanum lycopersicum,* Spe - *Solanum pennellii,* Stu – *Solanum tuberosum,* Ptc - *Populus trichocarpa*, Gma - *Glycine max.*

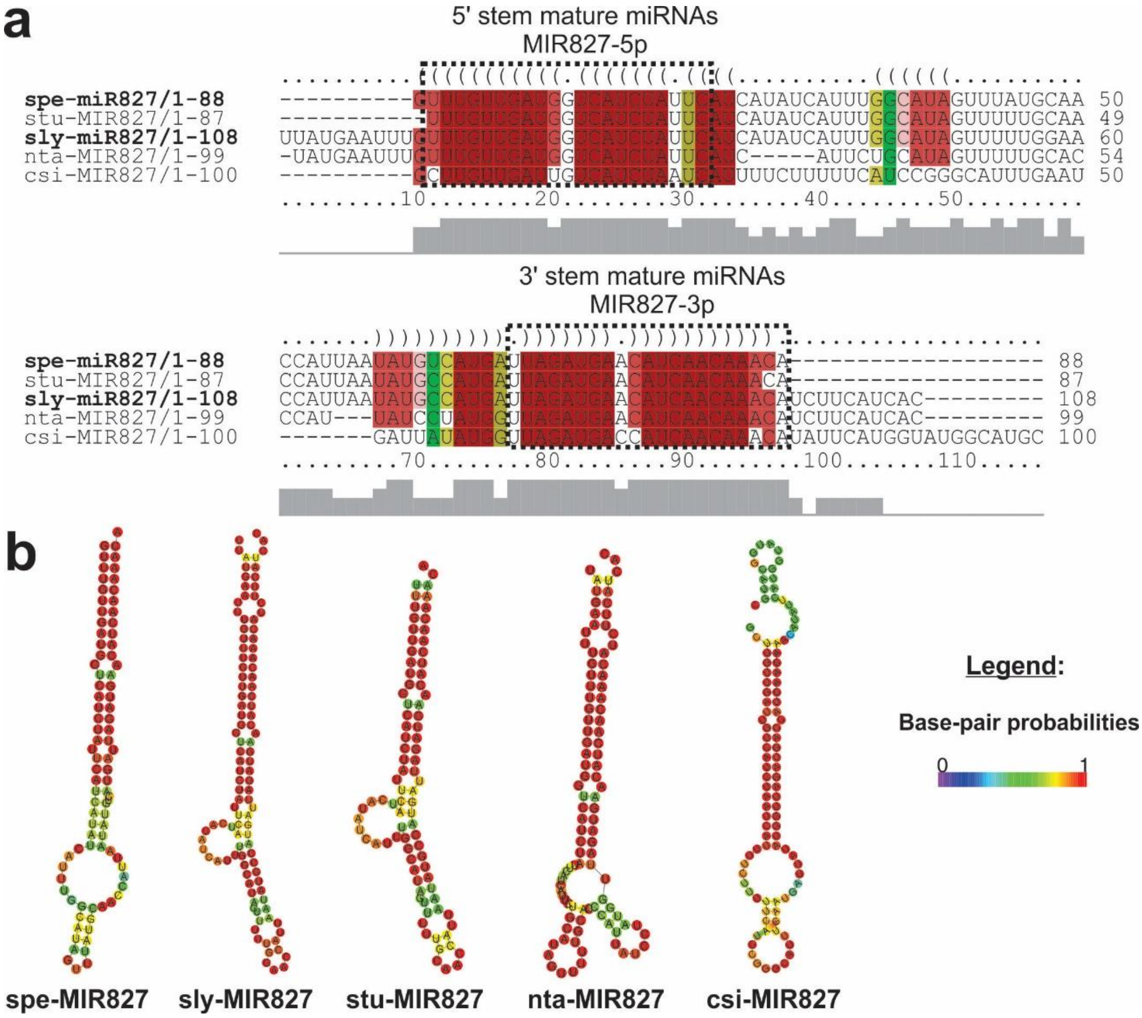

Supplementary Figure S5. (a) The high confidence of RNA alignments between MIR827 family of *S. lycopersicum* and *S. pennellii* to their orthologs were performed using RNAalifold. Mature miRNAs are shown as boxes. Brackets and colors identify matching residues in 5' and 3' stems of hairpin structures. Level of nucleotide identity is indicated below the alignment. **(b)** Secondary structures of MIR827 family from *S. lycopersicum, S. pennellii* and their orthologs were draw by RNAfold. Sly - *Solanum lycopersicum,* Spe - *Solanum pennellii,* Stu – *Solanum tuberosum,* Nta – *Nicotiana tabacum,* Csi - *Citrus sinensis*.

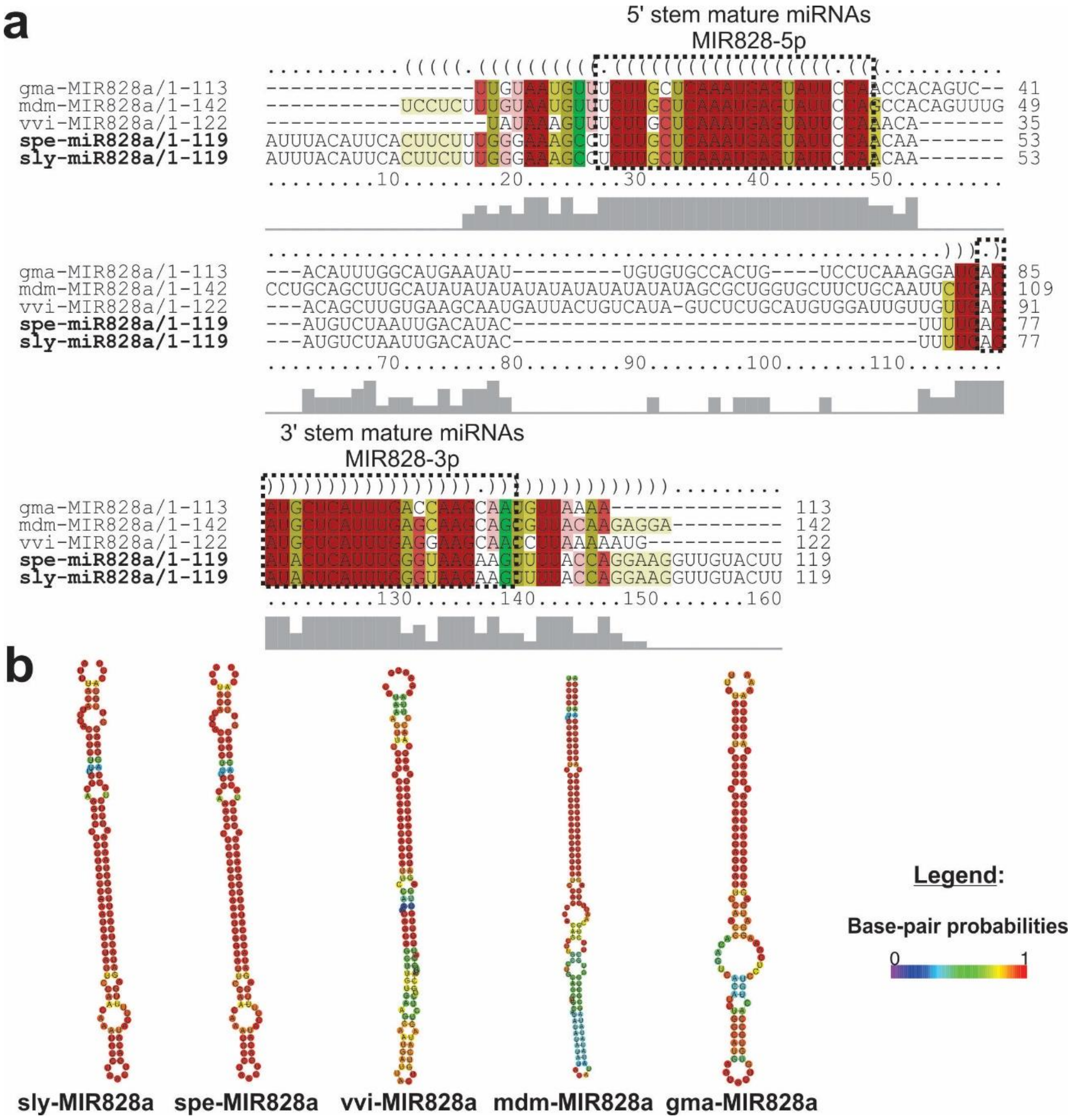

Supplementary Figure S6. (a) The high confidence of RNA alignments between MIR828 family of *S. lycopersicum* and *S. pennellii* to their orthologs were performed using RNAalifold. Mature miRNAs are shown as boxes. Brackets and colors identify matching residues in 5' and 3' stems of hairpin structures. Level of nucleotide identity is indicated below the alignment. **(b)** Secondary structures of MIR828 family from *S. lycopersicum, S. pennellii* and their orthologs were draw by RNAfold. Sly - *Solanum lycopersicum,* Spe - *Solanum pennellii,* Vvi – *Vitis vinifera*, Mdm - *Malus domestica*, Gma - *Glycine max*.

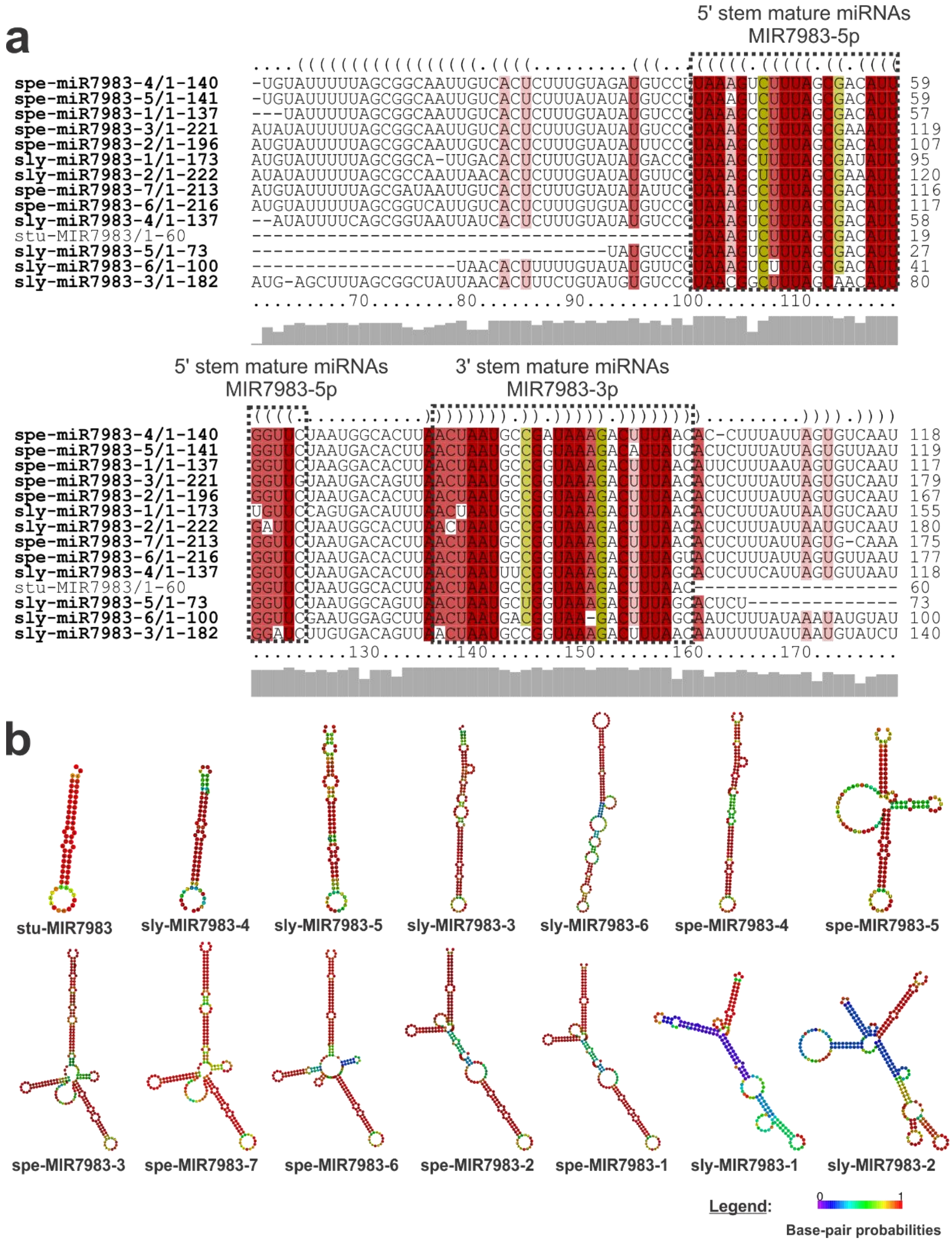

Supplementary Figure S7. (a) The high confidence of RNA alignments between MIR7983 family of *S. lycopersicum* and *S. pennellii* to their orthologs were performed using RNAalifold. Mature miRNAs are shown as boxes. Brackets and colors identify matching residues in 5’ and 3’ stems of hairpin structures. Level of nucleotide identity is indicated below the alignment. **(b)** Secondary structures of MIR7983 family from *S. lycopersicum, S. pennellii* and their orthologs were draw by RNAfold. Sly - *Solanum lycopersicum,* Spe - *Solanum pennellii,* Stu – *Solanum tuberosum*.

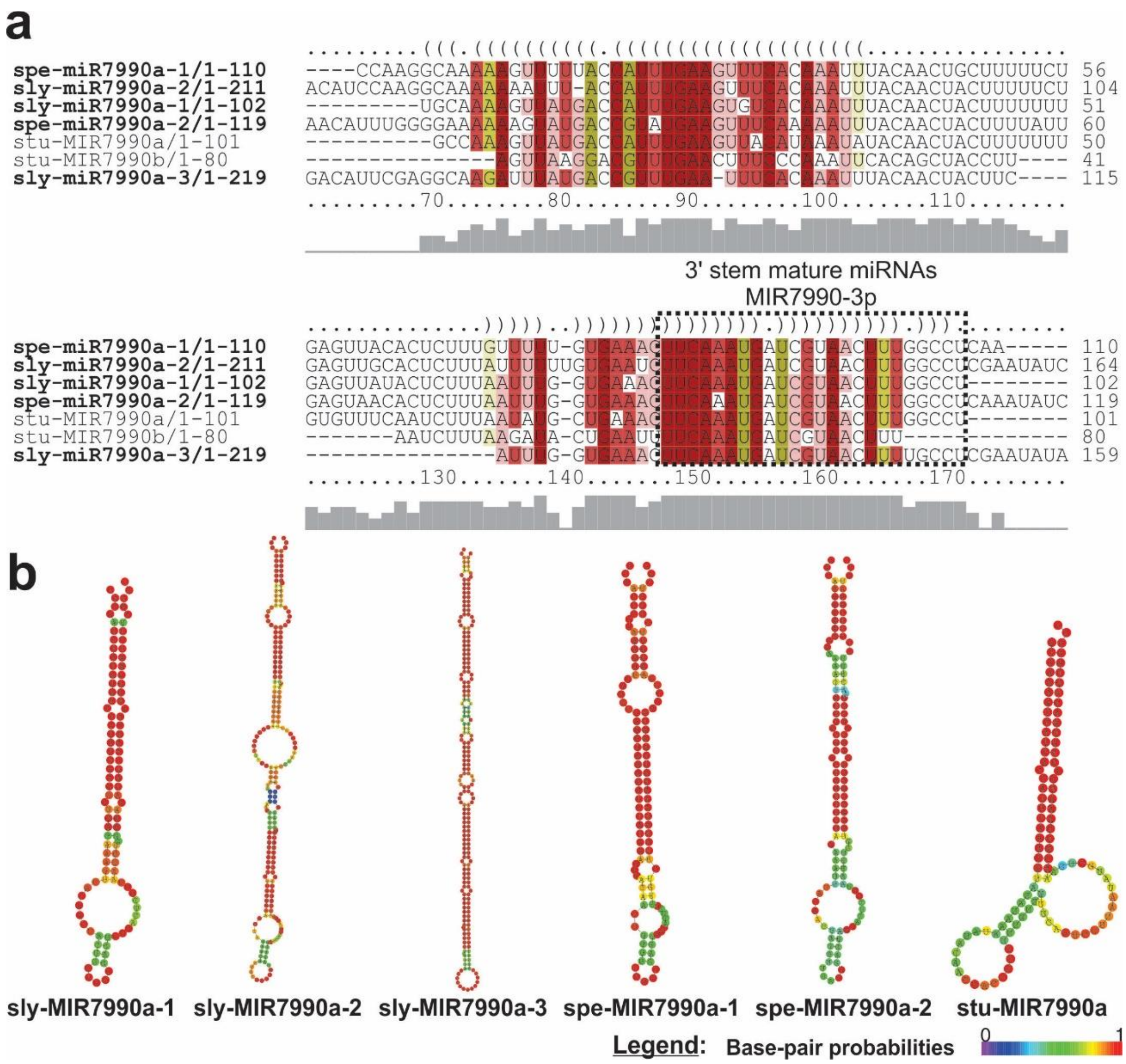

Supplementary Figure S8. (a) The high confidence of RNA alignments between MIR7990 family of *S. lycopersicum* and *S. pennellii* to their orthologs were performed using RNAalifold. Mature miRNAs are shown as boxes. Brackets and colors identify matching residues in 3' stems of hairpin structures. Level of nucleotide identity is indicated below the alignment. **(b)** Secondary structures of MIR7990 family from *S. lycopersicum, S. pennellii* and their orthologs were draw by RNAfold. Sly - *Solanum lycopersicum,* Spe - *Solanum pennellii,* Stu – *Solanum tuberosum*.

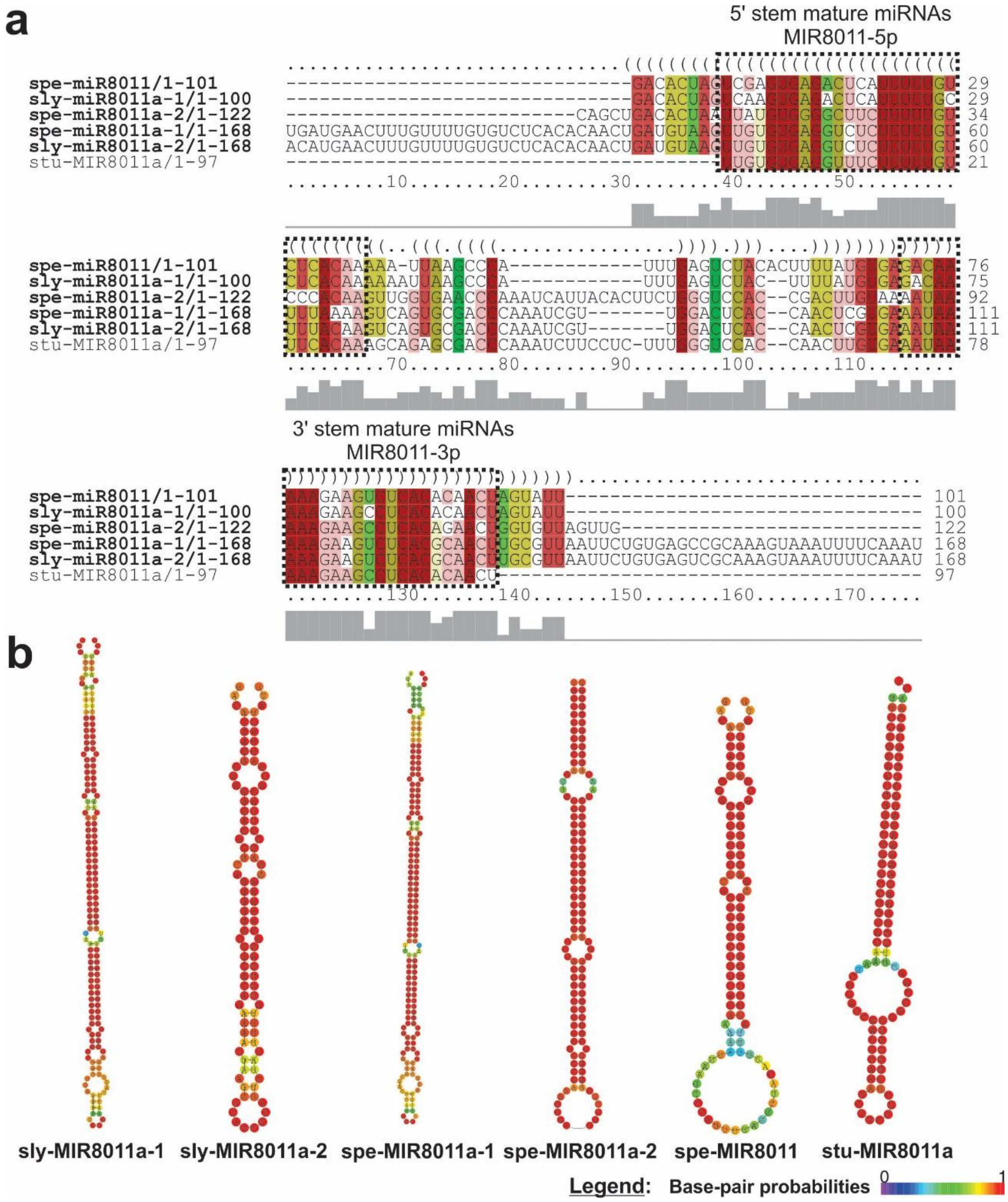

Supplementary Figure S9. (a) The high confidence of RNA alignments between MIR8011 family of *S. lycopersicum* and *S. pennellii* to their orthologs were performed using RNAalifold. Mature miRNAs are shown as boxes. Brackets and colors identify matching residues in 5' and 3' stems of hairpin structures. Level of nucleotide identity is indicated below the alignment. **(b)** Secondary structures of MIR8011 family from *S. lycopersicum, S. pennellii* and their orthologs were draw by RNAfold. Sly - *Solanum lycopersicum,* Spe - *Solanum pennellii,* Stu – *Solanum tuberosum*.

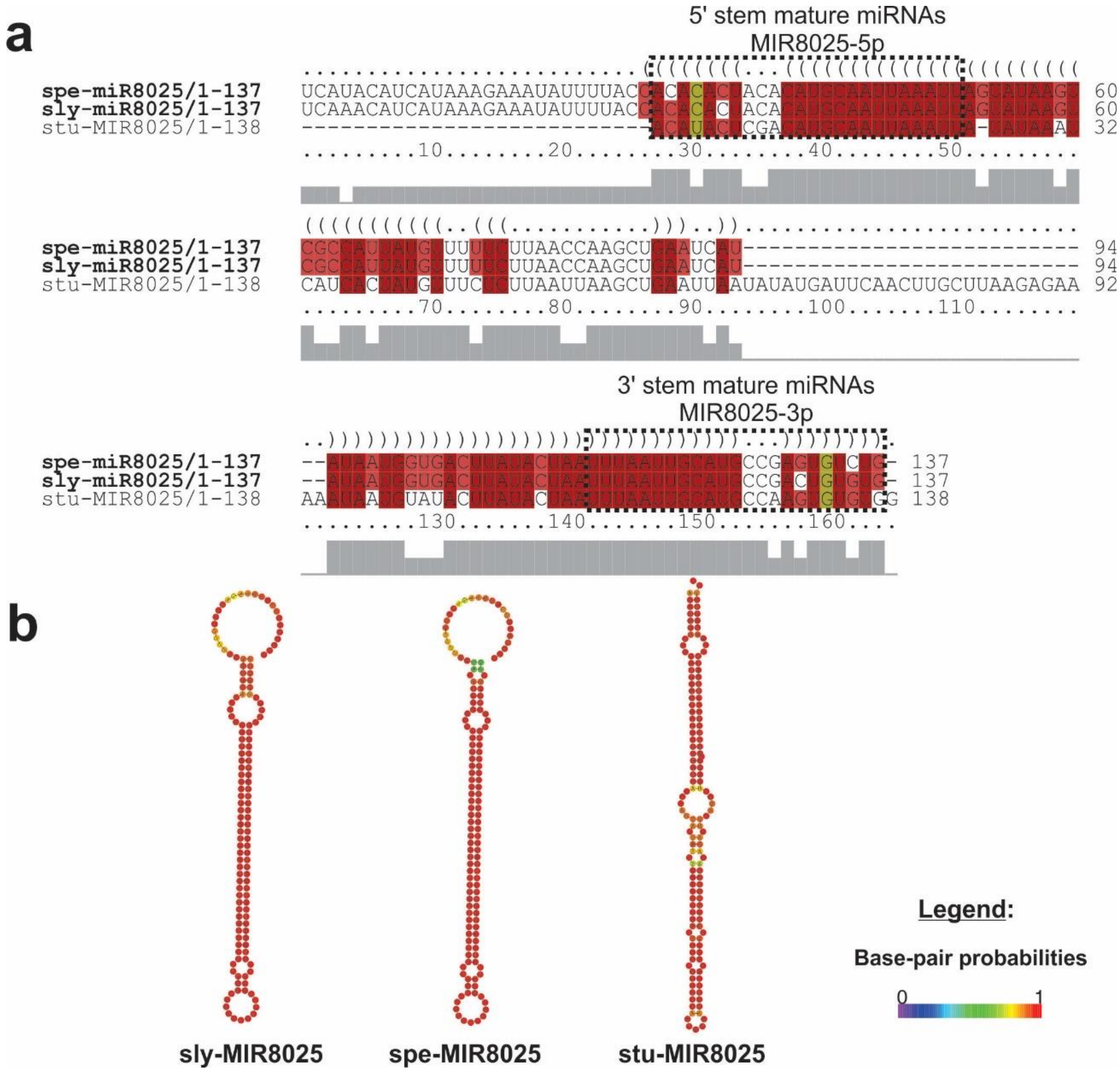

Supplementary Figure S10. (a) The high confidence of RNA alignments between MIR8025 family of *S. lycopersicum* and *S. pennellii* to their orthologs were performed using RNAalifold. Mature miRNAs are shown as boxes. Brackets and colors identify matching residues in 5' and 3' stems of hairpin structures. Level of nucleotide identity is indicated below the alignment. **(b)** Secondary structures of MIR8025 family from *S. lycopersicum, S. pennellii* and their orthologs were draw by RNAfold. Sly - *Solanum lycopersicum,* Spe - *Solanum pennellii,* Stu – *Solanum tuberosum*.

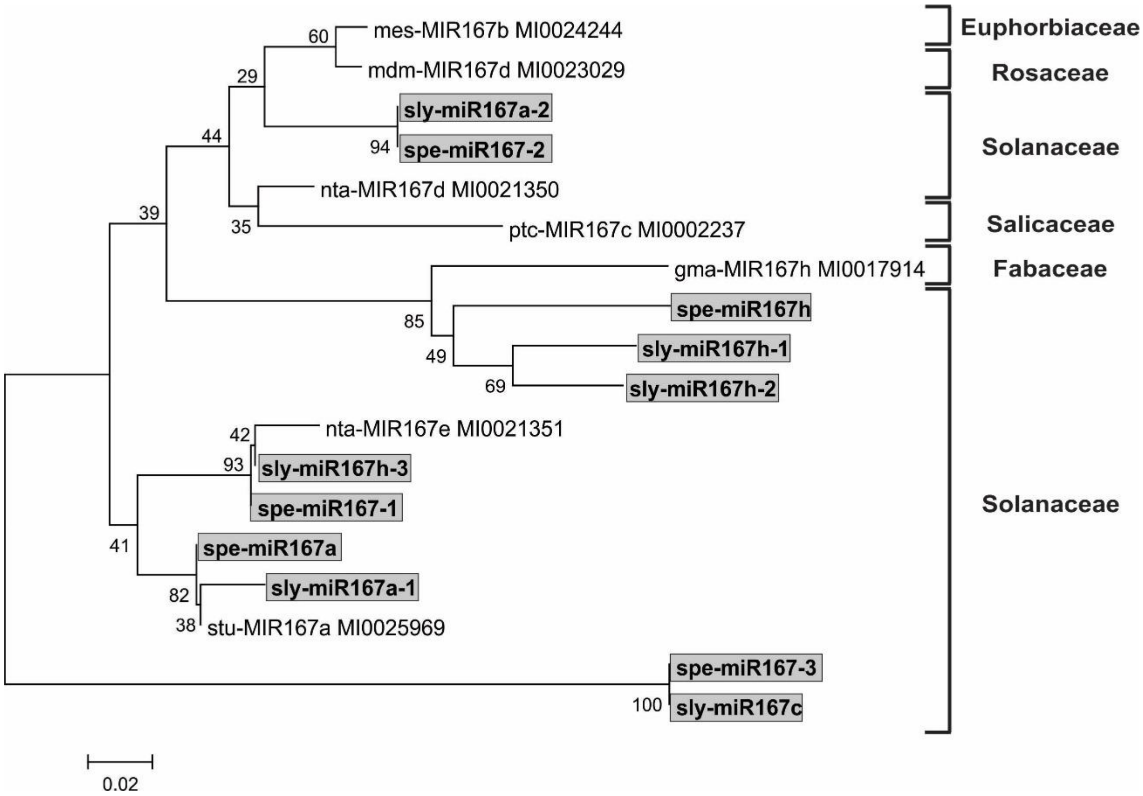

Supplementary Figure S11. Phylogenetic tree of MIR167 family identified in *S. lycopersicum, S. pennellii* and their orthologs.

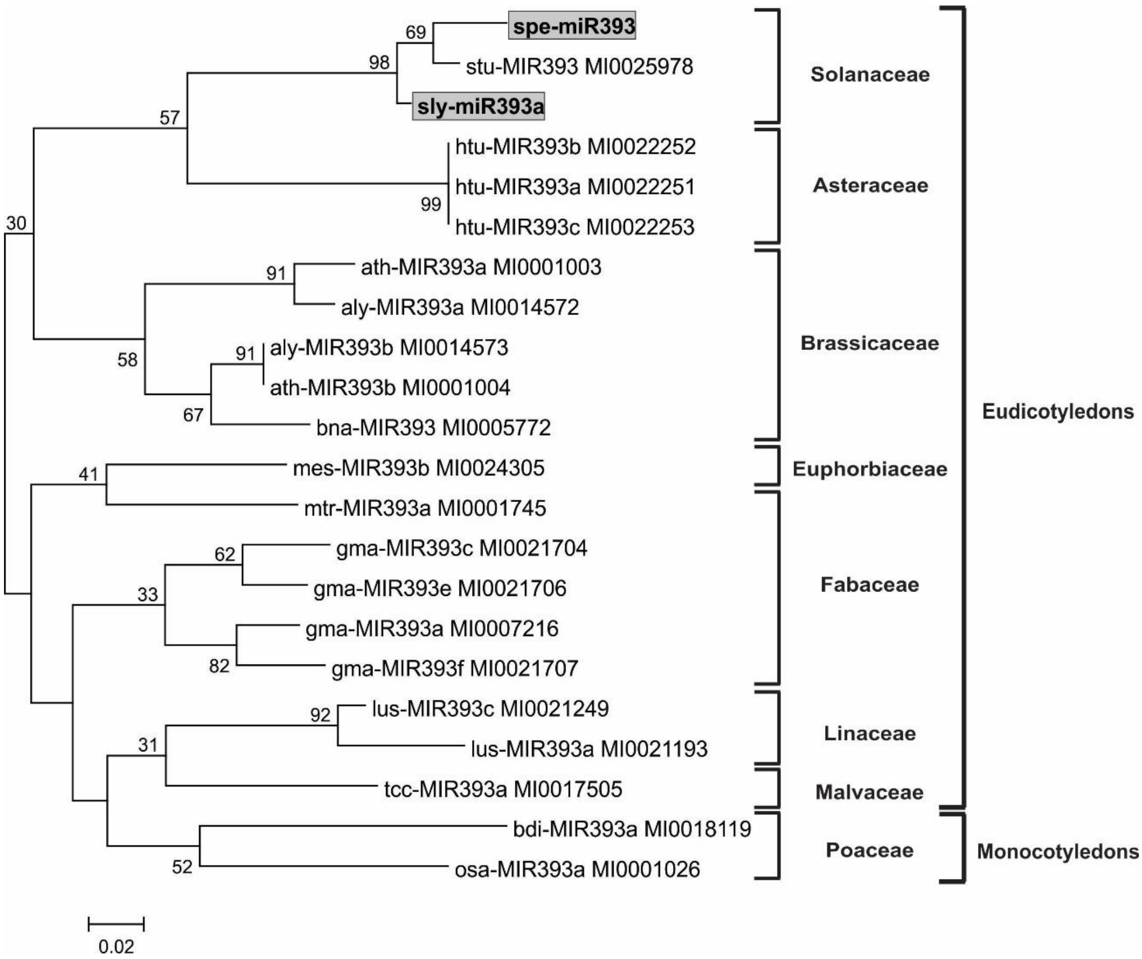

Supplementary Figure S12. Phylogenetic tree of MIR393 family identified in *S. lycopersicum, S. pennellii* and their orthologs.

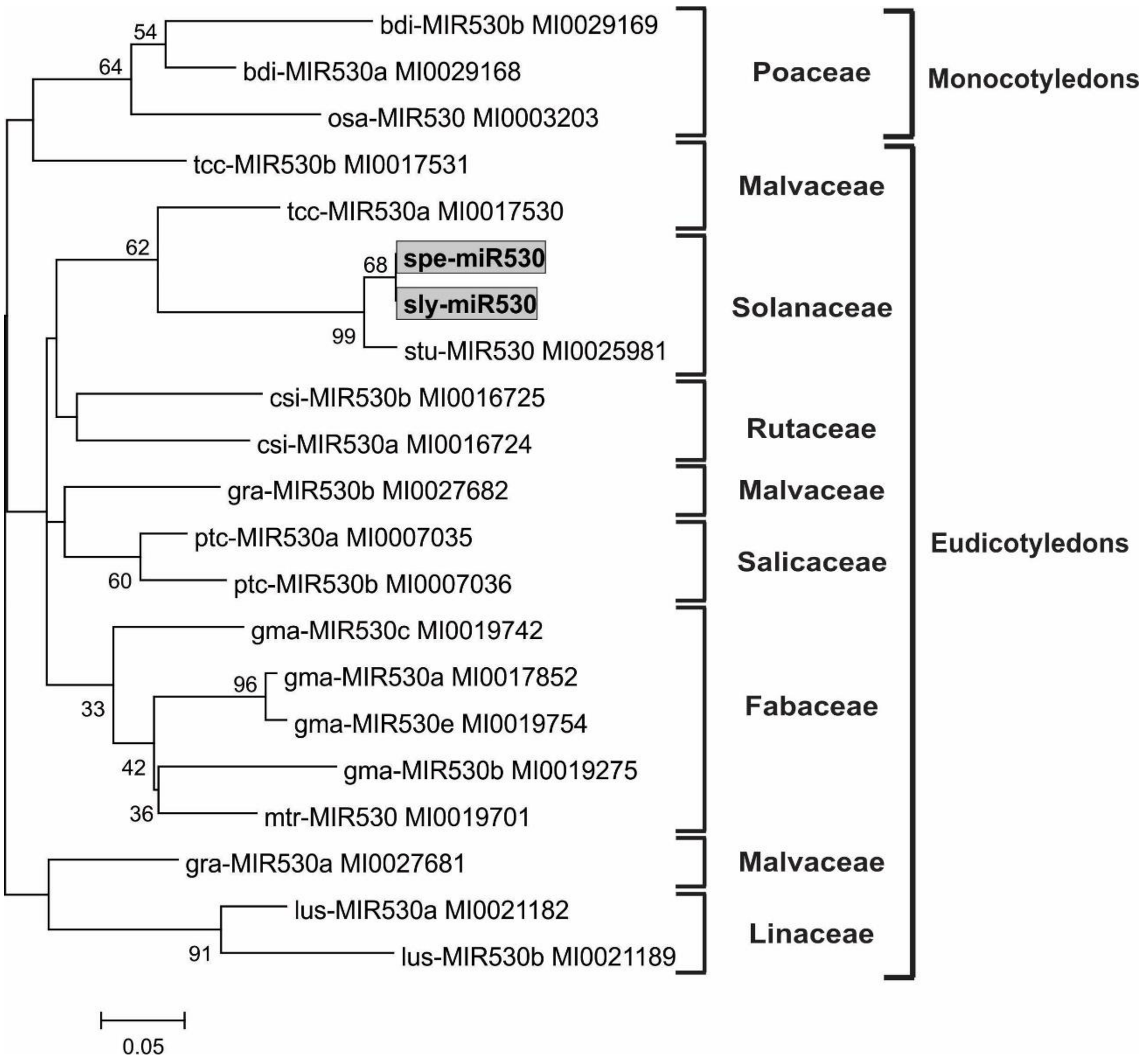

Supplementary Figure S13. Phylogenetic tree of MIR530 family identified in *S. lycopersicum, S. pennellii* and their orthologs.

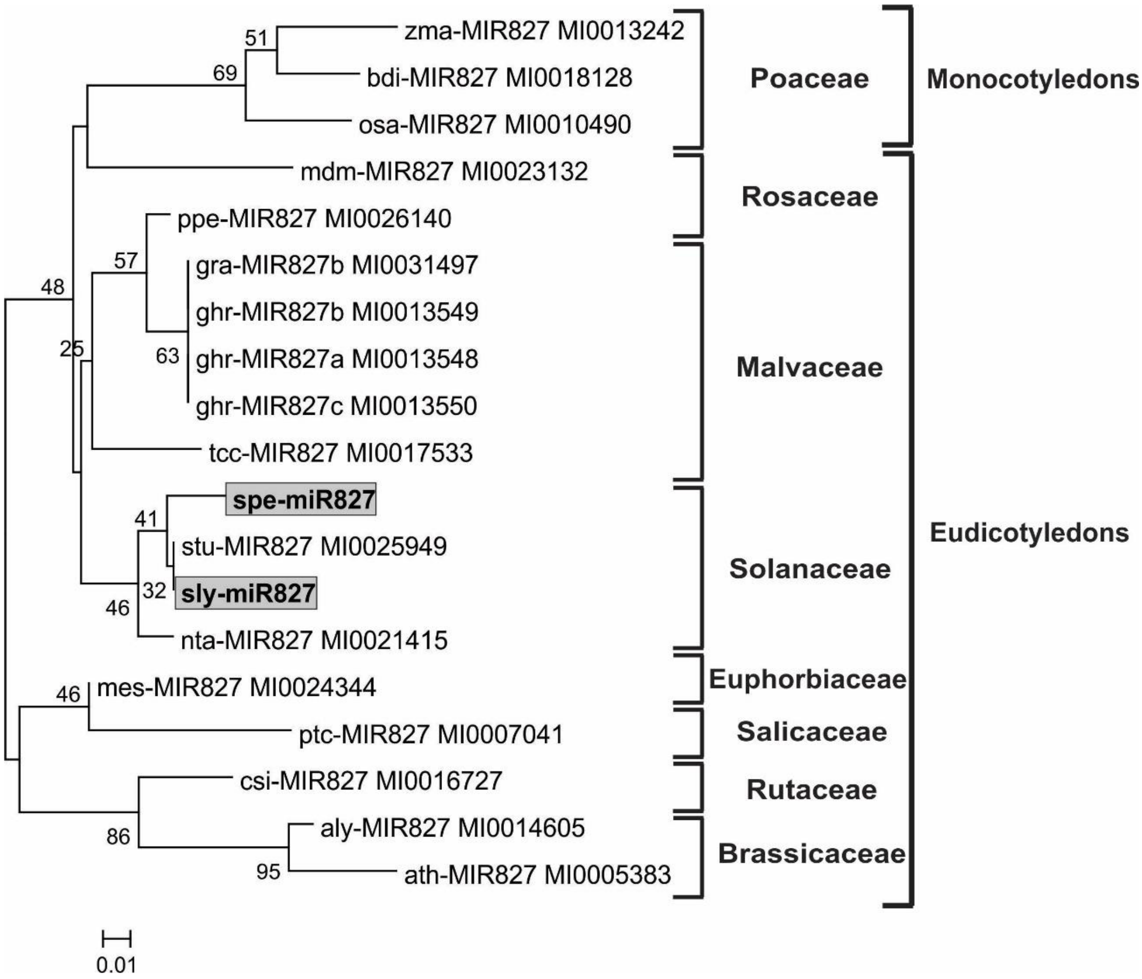

Supplementary Figure S14. Phylogenetic tree of MIR827 family identified in *S. lycopersicum, S. pennellii* and their orthologs.

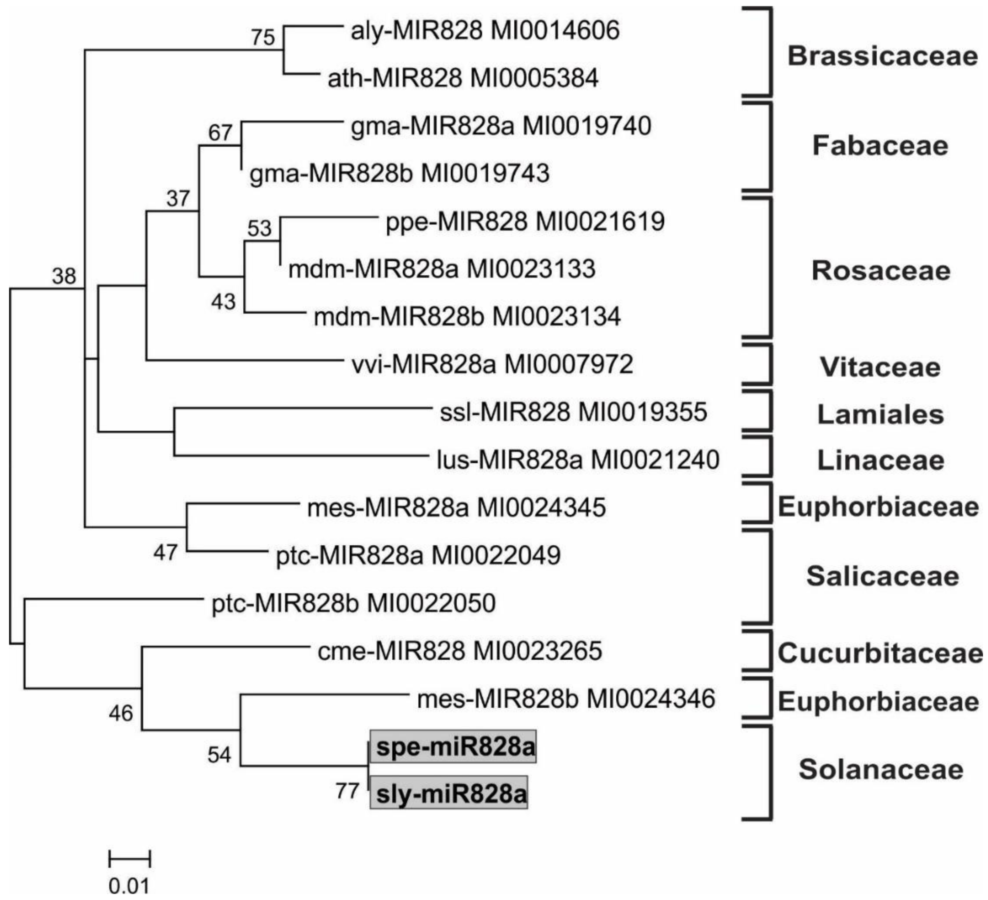

Supplementary Figure S15. Phylogenetic tree of MIR828 family identified in *S. lycopersicum, S. pennellii* and their orthologs.

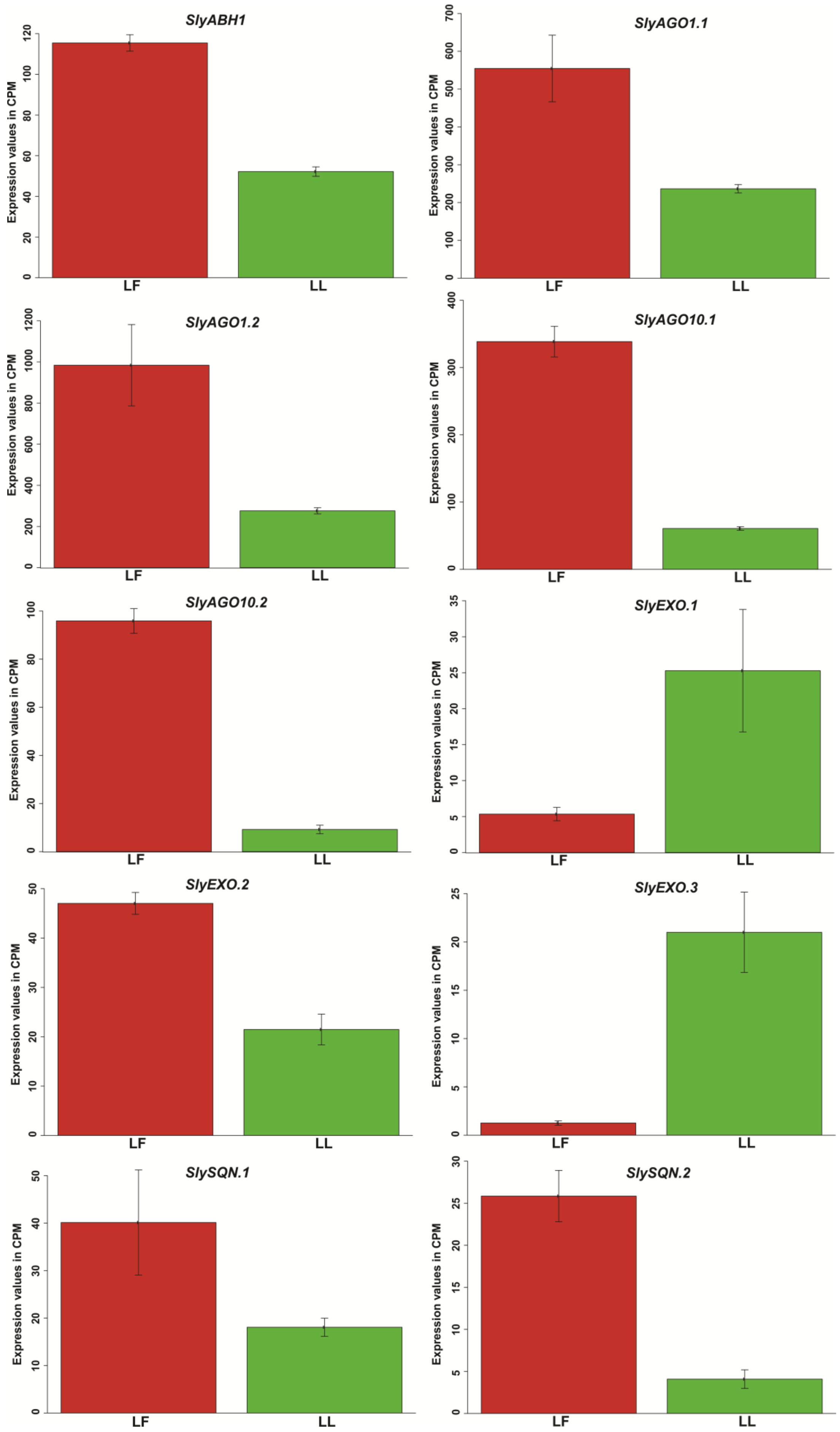

Supplementary Figure S16. Differential expression (DE) of the miRNAs pathway genes between *S. lycopersicum* flowers and leaves. LL (*S. lycopersicum* leaves in green), LF (*S. lycopersicum* flowers in red) and CPM (count per million).

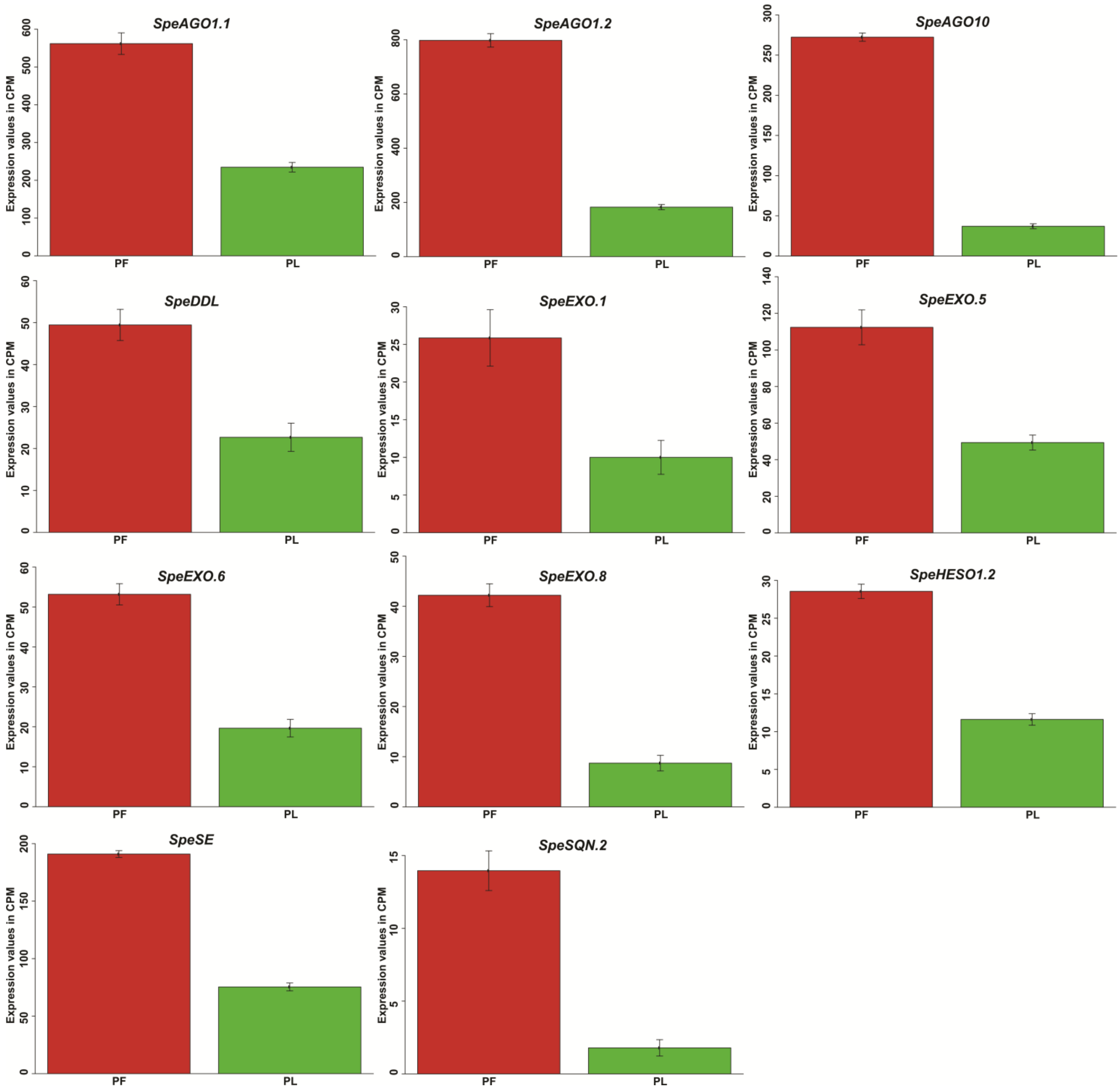

Supplementary Figure S17. Differential expression (DE) of the miRNAs pathway genes between *S. pennellii* flowers and leaves. PL (*S. pennellii* leaves in green) and PF (*S. pennellii* flowers in red) and CPM (count per million).

Supplementary Tables

Gene	ID NCBI of *S. lycopersicum*	Size (aa)	ID NCBI of *Arabidopsis thaliana*	Size (aa)	Blastp e-value: *A. thaliana* and *S. lycopersicum*	Ident	Genome	Chromosome	Database	Start position	End position	Strand	ID/Name
SlyABH1	XP_004248287	861	NP_565356.1	848	0.0	67%	ITAG3.0	Chr10	Sol Genomics	428279	445465	-	Solyc10g005530
SlyAGO1.1	NP_001266057.1	1054	NP_001185169.1	1050	0.0	78%	ITAG3.0	Chr06	Sol Genomics	44710986	44721105	-	Solyc06g072300
SlyAGO1.2	NP_001266261.2	1152	NP_001185169.1	1050	0.0	78%	ITAG3.0	Chr03	Sol Genomics	62062318	62069468	+	Solyc03g098280
SlyAGO10.1	XP_004247613.1	982	NP_001190464.1	988	0.0	81%	ITAG3.0	Chr09	Sol Genomics	68916353	68924564	-	Solyc09g082830
SlyAGO10.2	NP_001266268.1	939	NP_001190464.1	988	0.0	73%	ITAG3.0	Chr12	Sol Genomics	1199288	1214285	-	Solyc12g006790
SlyCBP20	XP_004233527.1	257	NP_001078705.1	257	$2e^{-128}$	75%	ITAG3.0	Chr02	Sol Genomics	34895133	34912181	-	Solyc02g062620
SlyCDC5	NP_001234729.2	987	NP_172448.1	844	0.0	72%	ITAG3.0	Chr04	Sol Genomics	3261426	3266792	+	Solyc04g009950
SlyCPL1	XP_004232844.1	954	NP_193898.3	967	0.0	58%	ITAG3.0	Chr02	Sol Genomics	43775820	43786429	-	Solyc02g078550
SlyDDL	XP_004248371.2	519	NP_188691.1	314	$9e^{-110}$	60%	ITAG3.0	Chr10	Sol Genomics	996419	1004741	-	Solyc10g006390
SlyDCL1	NP_001289827.1	1914	NP_171612.1	1909	0.0	72%	ITAG3.0	Chr10	Sol Genomics	111695	125349	+	Solyc10g005130
SlyEXO.1	XP_010320183.1	314	NP_192634.1	314	$2e^{-162}$	72%	ITAG3.0	Chr04	Sol Genomics	60523838	60524782	-	Solyc04g074450
SlyEXO.2	XP_010320182.1	312	NP_192634.1	314	$2e^{-160}$	72%	ITAG3.0	Chr04	Sol Genomics	60507541	60516683	+	Solyc04g074410
SlyEXO.3	XP_004237932.1	315	NP_192634.1	314	$4e^{-157}$	70%	ITAG3.0	Chr04	Sol Genomics	60520286	60521233	-	Solyc04g074440
SlyEXO.4	XP_004237930.1	309	NP_192634.1	314	$2e^{-114}$	55%	ITAG3.0	Chr04	Sol Genomics	60492687	60493616	-	Solyc04g074400
SlyHEN	XP_004233884.1	936	NP_001190782.1	942	0.0	49%	ITAG3.0	Chr02	Sol Genomics	40384527	40384937	-	Solyc02g070030

SlyHESO1.1	NP_001310843.1	453	NP_001318386.1	511	$4e^{-147}$	52%	ITAG3.0	Chr06	Sol Genomics	35528135	35547246	+	Solyc06g051790
SlyHESO1.2	XP_004250756.1	552	NP_001318386.1	511	$4e^{-38}$	32%	ITAG3.0	Chr11	Sol Genomics	32288016	32302403	-	Solyc11g044900
SlyHST	XP_004230145.1	1199	NP_187155.2	1202	0.0	65%	ITAG3.0	Chr01	Sol Genomics	88619826	88632083	+	Solyc01g098170
SlyHYL1	XP_004238005.1	433	NP_563850.1	419	$1e^{-76}$	61%	ITAG3.0	Chr04	Sol Genomics	61402144	61407360	+	Solyc04g076420
SlyNOT2.1	XP_010313541.1	628	NP_001077478.1	614	0.0	60%	ITAG3.0	Chr11	Sol Genomics	56388283	56396735	+	Solyc11g072940
SlyNOT2.2	XP_004241255.1	658	NP_001077478.1	614	0.0	65%	ITAG3.0	Chr06	Sol Genomics	47119921	47134159	+	Solyc06g075590
SlySE	XP_004228474.1	748	NP_565635.1	720	0.0	69%	ITAG3.0	Chr01	Sol Genomics	3028724	3042442	-	Solyc01g009090
SlySQN.1	NP_001316807.1	362	NP_565381.1	361	0.0	76%	ITAG3.0	Chr01	Sol Genomics	95581585	95586349	+	Solyc01g108340
SlySQN.2	XP_004232050.1	361	NP_565381.1	361	0.0	68%	ITAG3.0	Chr02	Sol Genomics	52648116	52656092	+	Solyc02g090480
SlySTA1	XP_004243341.1	1019	OAO98779.1	1029	0.0	75%	ITAG3.0	Chr07	Sol Genomics	62685798	62688857	+	Solyc07g054200
SlyTGH	XP_004236099.1	984	NP_001031926.1	900	0.0	54%	ITAG3.0	Chr03	Sol Genomics	65890464	65905320	+	Solyc03g114410
SlyVCS1.1	XP_004244016.1	1407	NP_187938.2	1344	0.0	55%	ITAG3.0	Chr07	Sol Genomics	67656674	67666715	+	Solyc07g065950
SlyVCS1.2	XP_004248289.1	1418	NP_187938.2	1344	0.0	53%	ITAG3.0	Chr10	Sol Genomics	703110	706856	+	Solyc10g005920
SlyXRN4	NP_001233774.1	978	NP_175851.1	947	0.0	63%	ITAG3.0	Chr04	Sol Genomics	39105433	39147113	-	Solyc04g049010

Supplementary Table S1. Identification of putative proteins involved in the miRNA pathway in *Solanum lycopersicum*. Identity (Ident)

Gene	ID NCBI of *S. pennellii*	Size (aa)	ID NCBI of *Arabidopsis thaliana*	Size (aa)	Blastp e-value: *A. thaliana* and *S. pennellii*	Ident	Genome	Chromosome	Database	Start position	End position	Strand	ID/Name
SpeABH1	XP_015055991.1	861	NP_565356.1	848	0.0	67%	Spenn_v2.0	Chr10	Sol Genomics	470562	487876	-	Sopen10g001510
SpeAGO1.1	XP_015079116.1	1053	NP_001185169.1	1050	0.0	78%	Spenn_v2.0	Chr06	Sol Genomics	55270253	55279596	-	Sopen06g028670
SpeAGO1.2	XP_015069669.1	1152	NP_001185169.1	1050	0.0	78%	Spenn_v2.0	Chr03	Sol Genomics	64779141	64793113	+	Sopen03g028990
SpeAGO10	XP_015088227.1	982	NP_001190464.1	988	0.0	81%	Spenn_v2.0	Chr09	Sol Genomics	80029833	80037384	-	Sopen09g031660
SpeCBP20	XP_015066864.1	145	NP_001078705.1	257	$8e^{-128}$	77%	Spenn_v2.0	Chr02	Sol Genomics	37046624	37052022	-	Sopen02g013650
SpeCDC5	XP_015073427.1	1002	NP_172448.1	844	0.0	72%	Spenn_v2.0	Chr04	Sol Genomics	3448797	3454313	+	Sopen04g005080
SpeCPL1	XP_015065799.1	954	NP_193898.3	967	0.0	58%	Spenn_v2.0	Chr02	Sol Genomics	46784394	46794934	-	Sopen02g023400
SpeDDL	XP_015056276.1	519	NP_188691.1	314	$4e^{-112}$	60%	Spenn_v2.0	Chr10	Sol Genomics	1070258	1078336	-	Sopen10g002340
SpeDCL1	XP_015055701.1	1914	NP_171612.1	1909	0.0	72%	Spenn_v2.0	Chr10	Sol Genomics	140256	153832	+	Sopen10g001150
SpeEXO.1	XP_015072851.1	314	NP_192634.1	314	$7e^{-154}$	72%	Spenn_v2.0	Chr04	Sol Genomics	71013460	71014602	-	Sopen04g029470
SpeEXO.2	XP_015072850.1	315	NP_192634.1	314	$8e^{-150}$	70%	Spenn_v2.0	Chr04	Sol Genomics	71008901	71009992	-	Sopen04g029460
SpeEXO.3	XP_015074412.1	301	NP_192634.1	314	$8e^{-145}$	67%	Spenn_v2.0	Chr01	Sol Genomics	104235554	104236833	+	Sopen01g047900
SpeEXO.4	XP_015072852.1	309	NP_192634.1	314	$1e^{-133}$	62%	Spenn_v2.0	Chr04	Sol Genomics	71016771	71028199	+	Sopen04g029480
SpeEXO.5	XP_015056292.1	344	NP_192634.1	314	$6e^{-173}$	72%	Spenn_v2.0	Chr10	Sol Genomics	74630529	74631994	-	Sopen10g027040
SpeEXO.6	XP_015067414.1	349	NP_192634.1	314	$2e^{-180}$	77%	Spenn_v2.0	Chr03	Sol Genomics	54987335	54988714	+	Sopen03g023370
SpeEXO.7	XP_015064271.1	338	NP_192634.1	314	$3e^{-130}$	64%	Spenn_v2.0	Chr02	Sol Genomics	42023523	42024843	-	Sopen02g017980

SpeEXO.8	XP_015077362.1	348	NP_192634.1	314	$5e^{-172}$	75%	Spenn_v2.0	Chr06	Sol Genomics	29018255	29019378	-	Sopen06g011130
SpeHEN	XP_015066899.1	936	NP_001190782.1	942	0.0	49%	Spenn_v2.0	Chr02	Sol Genomics	43139412	43149375	-	Sopen02g019190
SpeHESO1.1	XP_015079772.1	453	NP_001318386.1	511	$4e^{-148}$	53%	Spenn_v2.0	Chr06	Sol Genomics	44896865	44916062	+	Sopen06g017790
SpeHESO1.2	XP_015086026.1	611	NP_190161.2	682	$5e^{-107}$	47%	Spenn_v2.0	Chr09	Sol Genomics	82784741	82791899	+	Sopen09g034750
SpeHST	XP_015062207.1	1199	NP_187155.2	1202	0.0	65%	Spenn_v2.0	Chr01	Sol Genomics	990095019	99104849	+	Sopen01g041740
SpeHYL1	XP_015071372.1	406	NP_563850.1	419	$4e^{-78}$	62%	Spenn_v2.0	Chr04	Sol Genomics	71813898	71819068	+	Sopen04g030180
SpeNOT2.1	XP_015058689.1	628	NP_001077478.1	614	0.0	60%	Spenn_v2.0	Chr11	Sol Genomics	65966414	65974981	+,	Sopen11g030600
SpeNOT2.2	XP_015079397.1	658	NP_001077478.1	614	0.0	66%	Spenn_v2.0	Chr06	Sol Genomics	57856903	57867863	+	Sopen06g031990
SpeSE	XP_015060466.1	749	NP_565635.1	720	0.0	69%	Spenn_v2.0	Chr01	Sol Genomics	3437801	3451620	-	Sopen01g004580
SpeSQN.1	XP_015067254.1	362	NP_565381.1	361	0.0	77%	Spenn_v2.0	Chr01	Sol Genomics	106469253	106473850	+	Sopen01g050560
SpeSQN.2	XP_015067196.1	361	NP_565381.1	361	0.0	69%	Spenn_v2.0	Chr02	Sol Genomics	56319127	56325887	+	Sopen02g035160
SpeSTA1	XP_015082387.1	1019	OAO98779.1	1029	0.0	75%	Spenn_v2.0	Chr07	Sol Genomics	73824806	73829303	+	Sopen07g028130
SpeTGH	XP_015070455.1	984	NP_001031926.1	900	0.0	54%	Spenn_v2.0	Chr03	Sol Genomics	68747090	68761834	+	Sopen03g033510
SpeVCS.1	XP_015082036.1	1405	NP_187938.2	1344	0.0	54%	Spenn_v2.0	Chr07	Sol Genomics	78737896	78747556	+	Sopen07g034150
SpeVCS.2	XP_015055595.1	1418	NP_187938.2	1344	0.0	55%	Spenn_v2.0	Chr10	Sol Genomics	749356	761206	+	Sopen10g001880
SpeXRN4	XP_015073528.1	978	NP_175851.1	947	0.0	63%	Spenn_v2.0	Chr04	Sol Genomics	44928340	44965197	-	Sopen04g018450

Supplementary Table S2. Identification of putative proteins involved in the miRNA pathway in *Solanum pennellii*. Identity (Ident)

Characteristics	*Solanum pennellii*		*Solanum lycopersicum*		t-Test	
	**Mean**	**Median**	**Mean**	**Median**	***p-value***	**Conclusion**
**Size**	150.27	132.50	150.41	139.50	0.981>0.05	Same
**G**	20.00	19.64	19.96	19.84	0.889>0.05	Same
**A**	29.35	29.15	29.47	29.67	0.778>0.05	Same
**C**	18.11	17.74	18.11	17.48	0.993>0.05	Same
**U**	32.52	32.98	32.46	32.91	0.896>0.05	Same
**GC**	38.12	37.13	38.07	37.18	0.924>0.05	Same
**AU**	61.87	62.87	61.93	62.82	0.909>0.05	Same
**AU ratio**	1.14	1.12	1.13	1.12	0.740>0.05	Same
**GC ratio**	1.13	1.07	1.13	1.08	0.936>0.05	Same
**MFE**	-55.59	-52.00	-56.38	-55.80	0.613>0.05	Same
**MFEE**	-52.98	-49.35	-53.91	-51.70	0.532>0.05	Same
**Freq**	0.1040	0.0469	0.1181	0.0491	0.324>0.05	Same
**Div**	15.75	10.43	14.82	9.81	0.505>0.05	Same
**AMFE**	-39.12	-38.57	-39.42	-39.91	0.715>0.05	Same
**MFEI**	-1.027	-1.033	-1.040	-1.040	0.498>0.05	Same

Supplementary Table S10. Statistical analysis of the thermodynamic and structural characteristics of *S. lycopersicum* and *S. pennellii* pre-miRNAs (Size, G content, A content, C content, U content, GC content, AU content, AU ratio, GC ratio, Minimum Free Energy (MFE), Minimal Free Energy of the thermodynamic ensemble (MFEE), MFE structure Frequency in the ensemble (Freq), Ensemble Diversity (Div), Adjusted Minimum Free Energy (AMFE), Minimum Free Energy Index (MFEI)).

**Characteristics**	***Solanacea* family**		***Solanum lycopersicum***		**t-Test**	
	**Mean**	**Median**	**Mean**	**Median**	***p-value***	**Conclusion**
**MFE**	-52.89	-45.80	-56.48	-55.80	0.076>0.05	Same
**MFEE**	-51.32	-44.80	-53.91	-51.70	0.172>0.05	Same
	***Solanacea* family**		***Solanum pennellii***			
**MFE**	-52.89	-45.80	-55.59	-52.00	0.162>0.05	Same
**MFEE**	-51.32	-44.80	-52.98	-49.35	0.310>0.05	Same
	***Fabaceae* family**		***Solanum lycopersicum***			
**MFE**	-72.57	-54.50	-56.38	-55.80	0.000<0.05	Different
**MFEE**	-70.60	-52.30	-53.91	-51.70	0.000<0.05	Different
	***Fabaceae* family**		***Solanum pennellii***			
**MFE**	-72.57	-54.50	-55.59	-52.00	0.000<0.05	Different
**MFEE**	-70.60	-52.30	-52.98	-49.35	0.000<0.05	Different
	***Brassicaceae* family**		***Solanum lycopersicum***			
**MFE**	-69.76	-57.00	-56.38	-55.80	0.000<0.05	Different
**MFEE**	-67.45	-55.00	-53.91	-51.70	0.000<0.05	Different
	***Brassicaceae* family**		***Solanum pennellii***			
**MFE**	-69.76	-57.00	-55.59	-52.00	0.000<0.05	Different
**MFEE**	-67.45	-55.00	-52.98	-49.35	0.000<0.05	Different

Supplementary Table S11. Statistical analysis of the thermodynamic characteristics of *S. lycopersicum* and *S. pennellii* pre-miRNAs with the *Solanaceae*, *Fabaceae* and *Brassicaceae* pre-miRNAs (Minimum Free Energy (MFE) and Minimal Free Energy of the thermodynamic ensemble (MFEE)).

Gene	ID	Expression values in flowers (CPM)	Expression values in leaves (CPM)
SlyABH1	Solyc10g005530.3.1	115.444852	52.1872168
SlyAGO1.1	Solyc06g072300.3.1	554.549283	236.476941
SlyAGO1.2	Solyc03g098280.3.1	983.936678	276.205843
SlyAGO10.1	Solyc09g082830.3.1	338.57108	60.7240166
SlyAGO10.2	Solyc12g006790.2.1	95.8993628	9.21962841
SlyCBP20	Solyc02g062620.3.1	129.33665	91.3615657
SlyCDC5	Solyc04g009950.3.1	166.48385	116.105279
SlyCPL1	Solyc02g078550.3.1	60.6229746	48.663535
SlyDCL1	Solyc10g005130.3.1	91.7558091	104.242512
SlyDDL	Solyc10g006390.3.1	73.2100946	37.3722361
SlyEXO.1	Solyc04g074450.1.1	5.3392198	25.2812386
SlyEXO.2	Solyc04g074410.2.1	47.0192544	21.4652852
SlyEXO.3	Solyc04g074440.1.1	1.26156435	21.0011354
SlyEXO.4	Solyc04g074400.1.1	1.71196188	1.96351561
SlyHEN	Solyc02g070030.3.1	8.44937955	9.52887989
SlyHESO1.1	Solyc06g051790.3.1	23.0835192	17.3695524
SlyHESO1.2	Solyc11g044900.2.1	17.6155718	18.360006
SlyHST	Solyc01g098170.3.1	49.5060962	37.1582806
SlyHYL1	Solyc04g076420.3.1	39.4395615	25.6856802
SlyNOT2.1	Solyc11g072940.2.1	54.087307	44.9573852
SlyNOT2.2	Solyc06g075590.3.1	79.2242882	65.6660652
SlySE	Solyc01g009090.3.1	190.729155	100.339415
SlySQN.1	Solyc01g108340.3.1	40.1230857	18.0619814
SlySQN.2	Solyc02g090480.3.1	25.8472208	4.08469482
SlySTA1	Solyc07g054200.3.1	107.87164	77.08357
SlyTGH	Solyc03g114410.3.1	56.7897735	33.0774406
SlyVCS1.1	Solyc07g065950.3.1	167.794026	147.861092
SlyVCS1.2	Solyc10g005920.3.1	28.2916594	25.5557185
SlyXRN4	Solyc04g049010.3.1	54.6603746	33.1742515
SpeABH1	Sopen10g001510.1	74.17147916	41.82168983
SpeAGO1.1	Sopen06g028670.1	561.7262007	234.6132123
SpeAGO1.2	Sopen03g028990.1	797.8744787	182.5957222
SpeAGO10	Sopen09g031660.1	272.3394847	36.99650933
SpeCBP20	Sopen02g013650.1	14.01122266	13.14750259
SpeCPL1	Sopen02g023400.1	42.96757865	39.77289285
SpeDCL1	Sopen10g001150.1	79.92114549	86.06395606
SpeDDL	Sopen10g002340.1	49.45302454	22.66397733
SpeEXO.1	Sopen04g029470.1	25.86088596	10.0061042
SpeEXO.2	Sopen04g029460.1	0.454449558	0.00000000
SpeEXO.3	Sopen01g047900.1	52.23479504	42.49424417

SpeEXO.4	Sopen04g029480.1	9.531894255	10.19669835
SpeEXO.5	Sopen10g027040.1	112.3584832	49.36014175
SpeEXO.6	Sopen03g023370.1	53.16402678	19.64904854
SpeEXO.7	Sopen02g017980.1	2.368545132	1.402463911
SpeEXO.8	Sopen06g011130.1	42.19569417	8.716362738
SpeHEN	Sopen02g019190.1	29.98277927	22.95094378
SpeHESO1.1	Sopen06g017790.1	18.248202	26.6204945
SpeHESO1.2	Sopen09g034750.1	28.5515385	11.60838765
SpeHST	Sopen01g041740.1	3.966331996	3.452130078
SpeHYL1	Sopen04g030180.1	30.57804613	17.47057378
SpeNOT2.2	Sopen06g031990.1	0.50620271	1.353570116
SpeSE	Sopen01g004580.1	190.9284597	75.39198166
SpeSQN.1	Sopen01g050560.1	26.87909008	13.2713065
SpeSQN.2	Sopen02g035160.1	13.95862105	1.787305332
SpeSTA1	Sopen07g028130.1	109.6964154	68.49296034
SpeTGH	Sopen03g033510.1	37.88674519	34.73325799
SpeVCS.1	Sopen07g034150.1	202.3288705	118.2873192
SpeVCS.2	Sopen10g001880.1	129.9715733	140.5036149
SpeXRN4	Sopen04g018450.1	37.69853894	34.35767022

Supplementary Table S14. Expression profile values, showed in count per million (CPM), between flowers and leaves of the *Solanum lycopersicum* (Sly) and *Solanum pennellii* (Spe).

miRNA	miRNA sequence	RT primer	Forward	Reverse
**sly-miR166**	ucggaccaggcuucauucccc	GTCGTATCCAGTGCAGGGTCCGAGGTATTCGCACTGGATACGACGGGGAA	TTGTCGGACCAGGCTTCA	
**spe-miR166**	ucggaccaggcuucauuccuc	GTCGTATCCAGTGCAGGGTCCGAGGTATTCGCACTGGATACGACGAGGAA	TTGTCGGACCAGGCTTCA	
**sly-miR167**	ugaagcugccagcaugaucuaa	GTCGTATCCAGTGCAGGGTCCGAGGTATTCGCACTGGATACGACTTAGAT	CCTTGAAGCTGCCAGCATG	
**spe-miR167**	ugaagcugccagcaugaucua	GTCGTATCCAGTGCAGGGTCCGAGGTATTCGCACTGGATACGACTAGATC	CCTTGAAGCTGCCAGCAT	
**sly/spe-miR530**	ucugcauuugcaccugcaccu	GTCGTATCCAGTGCAGGGTCCGAGGTATTCGCACTGGATACGACAGGTGC	CGGTCTGCATTTGCACCT	
**sly/spe-miR7983**	acuaaugccgguaaagacuuuaac	GTCGTATCCAGTGCAGGGTCCGAGGTATTCGCACTGGATACGACGTTAAA	TCGACTAATGCCGGTAAAGAC	
**U6**			GGACATCCGATAAAATTGG	GATTTGTGCGTGTCATCCT
**Universal Primer**				GTGCAGGGTCCGAGGT
**5.8S**			GGGCGAGTCCAAAATCCAAT	GGTGTTTTCACGTCTTACCGT

Supplementary Table S16. Data sequence of the stem-loop RT primers designed according to Chen (2004).
